# MBNL1-mediated regulation of differentiation RNAs promotes myofibroblast transformation and the fibrotic response

**DOI:** 10.1038/ncomms10084

**Published:** 2015-12-16

**Authors:** Jennifer Davis, Nathan Salomonis, Natasha Ghearing, Suh-Chin J. Lin, Jennifer Q. Kwong, Apoorva Mohan, Maurice S. Swanson, Jeffery D. Molkentin

**Affiliations:** 1 Cincinnati Children's Hospital Medical Center, Department of Pediatrics, Cincinnati, Ohio 45229, USA; 2 Department of Molecular Genetics and Microbiology, Center for NeuroGenetics, University of Florida, College of Medicine, Gainesville, Florida 32610, USA; 3 Howard Hughes Medical Institute, Cincinnati Children's Hospital Medical Center, Cincinnati, Ohio Ohio 45229, USA

## Abstract

The differentiation of fibroblasts into myofibroblasts mediates tissue wound healing and fibrotic remodelling, although the molecular programme underlying this process remains poorly understood. Here we perform a genome-wide screen for genes that control myofibroblast transformation, and identify the RNA-binding protein muscleblind-like1 (MBNL1). MBNL1 overexpression promotes transformation of fibroblasts into myofibroblasts, whereas loss of *Mbnl1* abrogates transformation and impairs the fibrotic phase of wound healing in mouse models of myocardial infarction and dermal injury. Mechanistically, MBNL1 directly binds to and regulates a network of differentiation-specific and cytoskeletal/matrix-assembly transcripts to promote myofibroblast differentiation. One of these transcripts is the nodal transcriptional regulator serum response factor (SRF), whereas another is calcineurin Aβ. CRISPR-Cas9-mediated gene-editing of the MBNL1-binding site within the *Srf* 3′UTR impairs myofibroblast differentiation, whereas *in vivo* deletion of *Srf* in fibroblasts impairs wound healing and fibrosis. These data establish a new RNA-dependent paradigm for myofibroblast formation through MBNL1.

## Abstract (web summary)

Fibroblast-to-myofibroblast differentiation is crucial for wound healing and regeneration. Davis *et al*. describe a new regulatory mechanism underlying myofibroblast differentiation via the RNA-binding protein MBNL1, which promotes the maturation of certain mRNA transcripts that are integral nodes in fibroblast differentiation.

## Graphical abstract

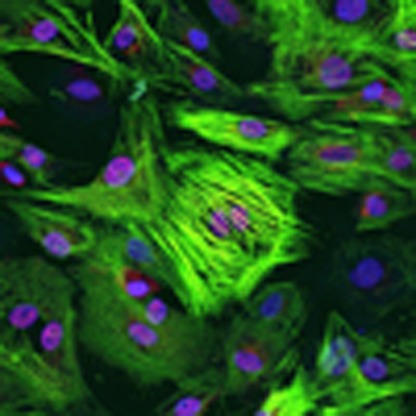

Most forms of cardiac injury and disease elicit a universal wound healing-like process similar to that described in the skin whereby extracellular matrix (ECM) is generated during a fibrotic response to modify the structural characteristics of the tissue^1^,^2^,^3^. After acute myocardial infarction (MI) injury to the heart, a wound-healing response is critical towards maintaining ventricular wall integrity, although in response to longstanding disease states the fibrotic response is pathological where it contributes to worsening cardiac performance and ultimately heart failure^4^,^5^,^6^,^7^,^8^,^9^. The fibrotic response and wound healing are regulated by the myofibroblast, a highly specialized cell type that arises from quiescent tissue-resident fibroblasts or other poorly differentiated epithelial/endothelial cells or select immune cells^2^,^3^. Myofibroblasts then secrete large quantities of ECM and other matricellular proteins within the area of injury, and thereafter bind to and contract this network to mediate tissue remodelling and wound closure^2^,^3^,^5^,^10^. The contractile activity of myofibroblasts is due to induction of genes encoding smooth muscle α-actin (αSMA) and embryonic smooth muscle myosin, among many others^1^,^2^,^3^,^5^.

A fundamental initiator of myofibroblast differentiation is the cytokine transforming growth factor β (TGFβ), which is part of a cytokine milieu that is generated within an injury environment. TGFβ initiates intracellular signalling through both canonical SMAD and non-canonical mitogen-activated protein kinase (MAPK)-p38 kinase signalling^4^,^5^. Although the myofibroblast and fibrosis literature are biased towards canonical TGFβ signalling, there is clearly an extensive network of other molecular regulators in play. These additional regulatory pathways include actin cytoskeleton stress-sensing through the GTPase Rho and downstream effectors such as myocardin-related transcription factors and serum response factor (SRF)^11^, as well as Ca^2+^ signalling mediated by calcineurin^4^. To generate a more comprehensive model of the entire gene network mediating myofibroblast differentiation, we employed a genome-wide gain-of-function screen in quiescent fibroblasts, which identified muscleblind-like1 (MBNL1).

MBNL1 is a highly conserved RNA-binding protein that modulates alternative splicing, alternative polyadenylation, mRNA stability and mRNA localization by directly binding to select transcripts^12^,^13^,^14^. The concept that MBNL1 has selective mRNA targets is consistent with its known role in regulating striated muscle differentiation^15^,^16^,^17^,^18^ and the transition from fetal to matured cardiac muscle^19^,^20^. MBNL1 was also recently shown to negatively regulate the pluripotency of embryonic stem cells by directing alternative splicing of select transcripts^21^,^22^. Indeed, the loss of MBNL1-mediated alternative splicing in skeletal muscle causes the inappropriate expression of fetal transcript variants, resulting in severe pathology reminiscent of myotonic dystrophy^16^,^23^.

Here, we demonstrate that MBNL1 is induced within the fibroblast during an injury response to bind and regulate a selective population of mRNAs that directly programme myofibroblast differentiation. MBNL1 regulated the maturation of the mRNA transcript for SRF and calcineurin Aβ, which increased their apparent activity and augmented myofibroblast differentiation. Indeed, deletion of the MBNL1-binding site in the 3′ untranslated (UTR) of the SRF transcript reduced the efficiency of myofibroblast differentiation.

## Results

### MBNL1 promotes myofibroblast differentiation

**Figure 1:**
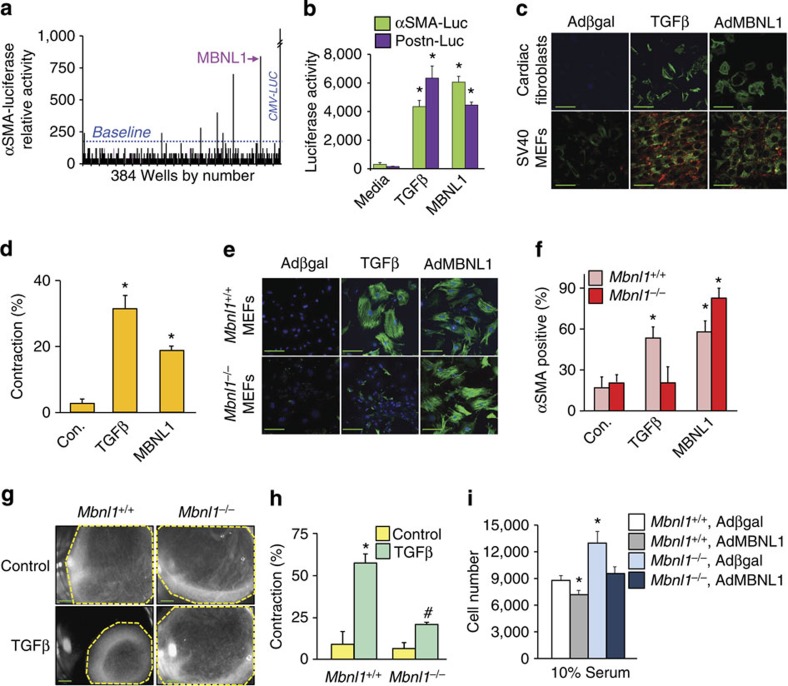
MBNL1 overexpression mediates myofibroblast transformation. (**a**) A schematic of myofibroblast differentiation measured by αSMA-luciferase activity from one 384-well plate of the Mammalian Genome Collection cDNA library, which identified MBNL1 as a potent inducer of the reporter. (**b**) αSMA-luciferase and periostin (*Postn*)-luciferase promoter activity in cultured cardiac fibroblasts transfected with a MBNL1 expression plasmid or treated with TGFβ. Average light units±s.e.m., *N*=3, analysis of variance (ANOVA) and Newman–Keuls pairwise analysis, *P<0.05 versus media control. (**c**) Immunofluorescence for αSMA^+^ stress fibres (green) in cardiac fibroblasts and SV40-transformed MEFs treated with TGFβ or infected with Adβgal (control) or AdMBNL1. SV40 MEFs were also stained with collagen 1 (red). (**d**) Quantification of collagen gel matrix contraction in cardiac fibroblasts treated with TGFβ, AdMBNL1 or Adβgal (Con.) 48 h later. Average contraction±s.e.m., *N*=3, ANOVA and Newman–Keuls pairwise analysis, *P<0.05 versus Con. (**e**,**f**) Immunofluorescent images and quantification of αSMA-positive stress fibres (green) in primary *Mbnl1*^*+/+*^ and *Mbnl1*^−/−^ MEFs with TGFβ treatment or AdMBNL1 infection. Controls were infected with Adβgal. Blue is staining for nuclei (Topro-3 Iodide). Bars represent the average number of fibroblasts with αSMA^+^ stress fibres relative to the total number of nuclei±s.e.m., *N*⩾330, ANOVA and Newman–Keuls pairwise analysis, *P<0.05 versus Adβgal (Con.). (**g**,**h**) Photographs and quantification of contraction of collagen matrices seeded with *Mbnl1*^*+/+*^ and *Mbnl1*^−/−^ MEFs with or without 48 h of TGFβ treatment. Average contraction±s.e.m., *N*=3, ANOVA and Newman–Keuls pairwise analysis, *P<0.05 versus no treatment control. (**i**) Proliferation rates in *Mbnl1*^*+/+*^ and *Mbnl1*^−/−^ MEFs measured by colorimetric MTT assay 48 h after AdMBNL1 or Adβgal (control) infection. Average cell number±s.e.m., *N*=3, ANOVA and Newman–Keuls pairwise analysis, *P<0.05 versus *Mbnl1*^*+/+*^, Adβgal. All scale bars, 50 μm.

To identify novel mediators of myofibroblast differentiation, the Mammalian Genome Collection cDNA library was screened in quiescent SV40-transformed mouse embryonic fibroblasts (MEFs) under serum-reduced conditions with an αSMA promoter-containing luciferase reporter plasmid as a transcriptional readout for myofibroblast transformation (Fig. 1a). Baseline activity was measured in fibroblasts that were transfected with αSMA-luc and an empty plasmid (control), and all values were compared with this basal level of promoter activity. Out of 18,400 cDNAs, less than 1% produced αSMA promoter induction greater or equal to TGFβ stimulation, with one of the highest scoring candidates encoding the gene product MBNL1 (Fig. 1a,b). Another genetic marker of the myofibroblast is periostin (*Postn* gene), which was also induced at the promoter level by MBNL1 (Fig. 1b). To functionally confirm these results, MBNL1 was overexpressed using a recombinant adenovirus (AdMBNL1) in serum-reduced rat cardiac fibroblasts and SV40 transformed MEFs. MBNL1 overexpression or treatment with TGFβ induced 50–60% of the fibroblasts to develop αSMA-positive stress fibres, a marker for myofibroblast transformation, compared with very low levels of background αSMA-expressing fibroblasts using a control β-galactosidase expressing adenovirus (Adβgal; Fig. 1c). MBNL1-expressing cardiac fibroblasts were also able to contract collagen gel matrices similar to TGFβ treatment, demonstrating the acquisition of a contractile myofibroblast phenotype (Fig. 1d).

To determine if MBNL1 is required for fibroblast to myofibroblast transformation, primary MEFs isolated from *Mbnl1*^−/−^ and wild-type (*Mbnl1*^*+/+*^) mice were treated with TGFβ and examined for αSMA stress fibre expression (Fig. 1e,f) and contractile function (Fig. 1g,h). Remarkably, *Mbnl1*^−/−^ MEFs were unable to form αSMA stress fibres or contract a collagen matrix in response to TGFβ (Fig. 1e–h). Reconstituting MBNL1 back into *Mbnl1*^−/−^ MEFs by adenoviral gene transfer restored the ability of these cells to again form myofibroblasts (Fig. 1e,f). Consistent with the interplay between differentiation and proliferation, *Mbnl1*^−/−^ MEFs had a higher rate of proliferation in comparison to wild type, whereas overexpression of MBNL1 limited the proliferation capacity of both *Mbnl1*^*+/+*^ and *Mbnl1*^−/−^ MEFs because of the promotion of their differentiated state (Fig. 1i).

### MBNL1-mediated myofibroblast formation in wound healing

**Figure 2:**
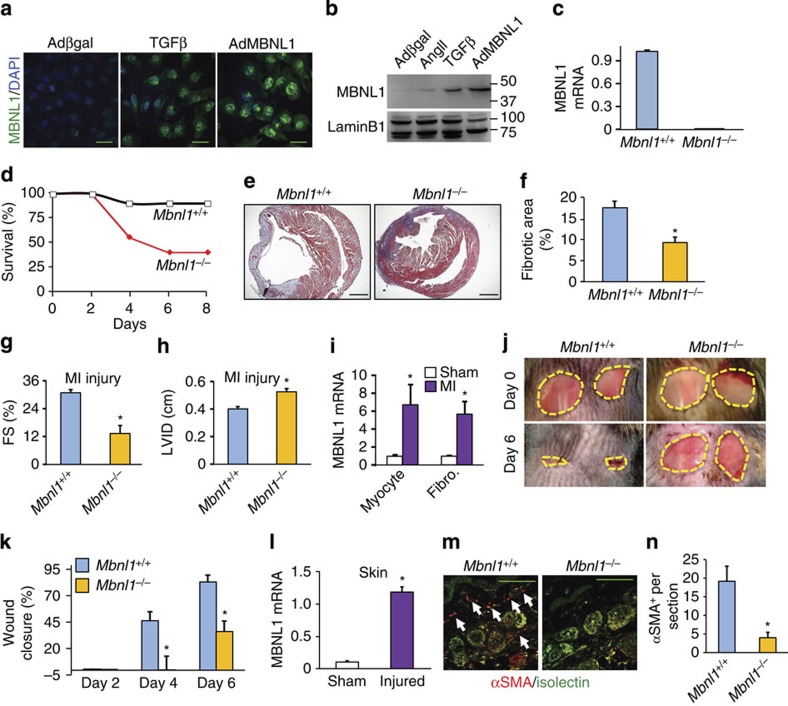
Deletion of *Mbnl1* abrogates the fibrotic response and wound healing *in vivo.* (**a**) Immunofluorescence for MBNL1 (green) and nuclei (blue) in Adβgal (control), TGFβ-treated and AdMBNL1-transduced cardiac fibroblasts. Scale bar, 50 μm. (**b**) Western blot for the nuclear levels of MBNL1 in cardiac fibroblasts treated with angiotensin II (AngII), TGFβ and AdMBNL1. LaminB1 is a nuclear loading control. Molecular weight sizing in kDa is shown on the right of the blots. The panel here is referenced in Supplementary Fig. 1. (**c**) MBNL1 mRNA levels in *Mbnl1*^*+/+*^ or *Mbnl1*^−/−^ cardiac fibroblasts. (**d**) Survival rates in *Mbnl1*^*+/+*^ and *Mbnl1*^−/−^ mice after myocardial infarction (MI) injury, *N*=15. (**e**,**f**) Photographs and quantification of the area of fibrosis (blue) in transverse histological sections of infarcted *Mbnl1*^*+/+*^ and *Mbnl1*^−/−^ hearts stained with Masson's trichrome. Average fibrotic area±s.e.m., *N*⩾6, *t*-test, *P<0.05 versus *Mbnl1*^*+/+*^. Scale bar, 1 mm. (**g**,**h**) Echocardiography in MI-injured *Mbnl1*^*+/+*^ and *Mbnl1*^−/−^ mice for ventricular fractional shortening (FS%) and left ventricle inner diameter (LVID). Data are averages±s.e.m., *N*⩾6, *t*-test, *P<0.05 versus *Mbnl1*^*+/+*^. (**i**) MBNL1 mRNA from isolated adult cardiomyocytes or fibroblasts (Fibro.) from sham or MI-injured mouse hearts. Data are averages±s.e.m., *N*=3 mice, *t*-test, *P<0.05 versus Sham. (**j**) Photographs of two dorsal skin punch biopsies on adult *Mbnl1*^*+/+*^ and *Mbnl*^−/−^ mice at days 0 and 6 of injury. The distance across the biopsy at day 0 is 6 mm, to provide scaling of the photograph. (**k**) Quantification of dermal would closure over time measured relative to initial wound size. Average closure±s.e.m., *N*⩾7, analysis of variance and Newman–Keuls pairwise analysis, *P<0.05 versus *Mbnl1*^*+/+*^. (**l**) Average MBNL1 mRNA levels in injured skin normalized to Gapdh expression±s.e.m., *N*=3, *t*-test, *P<0.05 versus sham. (**m**,**n**) Immunofluorescent staining and quantification of myofibroblasts in the border zone of biopsied skin 4 days after wounding. Myofibroblasts are αSMA (red) positive and negative for the epithelia marker isolectin (green). Data represent the average number of αSMA^+^/isolectin^−^ cells per section±s.e.m., four sections were measured per wound, *N*⩾7 mice per group, *t*-test, *P<0.05 versus *Mbnl1*^*+/+*^. Scale bar, 250 μm.

The observation that MBNL1 was necessary for myofibroblast differentiation in cultured fibroblasts suggested that this factor might underlie the fibrotic response and wound healing *in vivo*. Immunofluorescent imaging demonstrated that quiescent cardiac fibroblasts express minimal amounts of MBNL1 but that TGFβ treatment significantly increased expression, which was predominantly within the nucleus, similar to the immunofluorescent imaging of AdMBNL1-infected and overexpressing fibroblasts (Fig. 2a). Western blot analysis for MBNL1 also showed strong induction of expression in fibroblasts treated with either TGFβ or a second pro-fibrotic stimulus, angiotensin II (AngII), relative to Adβgal-infected control cultures (Fig. 2b). Full-length gel file of the data in Fig. 2b is also available for inspection (Supplementary Fig. 1).

To directly examine wound healing *in vivo*, we employed a mouse model of MI injury, which induces a fulminant fibrotic response to preserve cardiac ventricular wall integrity. As might be predicted by the *in vitro* results, MI-injured *Mbnl1*^−/−^ mice, which lacked translatable MBNL1 mRNA (Fig. 2c) as previously shown^24^,^25^, had a high rate of mortality because of ventricular wall rupture relative to wild-type littermates (Fig. 2d). Moreover, surviving MI-injured *Mbnl1*^−/−^ mice had a significant reduction in fibrotic scarring as determined by Masson's trichrome staining (Fig. 2e,f), as well as a reduction in ventricular performance and chamber dilation measured by echocardiography (Fig. 2g,h). MI injury also caused induction of MBNL1 mRNA expression in both isolated cardiomyocytes and fibroblasts from these hearts, relative to sham mice (Fig. 2i). Collectively, these data suggest that MBNL1 is a critical factor underlying the acute wound-healing process, as loss of this gene product renders mice more susceptible to ventricular wall rupture and greater cardiomyopathy in surviving mice, consistent with impaired myofibroblast differentiation.

We also employed a dermal injury model in the mouse to address whether MBNL1 might underlie a more universal wound-healing response through its ability to regulate myofibroblast differentiation. *Mbnl1*^−/−^ and *Mbnl1*^*+/+*^ mice were given two adjacent 6 mm dermal punch biopsies, and wound size was analysed over a period of 6 days. Wild-type mice showed a progressive closure to ∼80% by the end of 6 days, whereas *Mbnl1*^−/−^ mice had a significant delay in wound healing, achieving only 40% closure (Fig. 2j,k). At the midpoint of the acute wound-healing response, we found that MBNL1 expression was strongly upregulated in the border zone of the injured skin (Fig. 2l). The border zone was also analysed for the presence of myofibroblasts using immunofluorescent imaging of αSMA (red) and a marker for endothelial cells (green), which showed that *Mbnl1*^−/−^ mice had negligible numbers of myofibroblasts relative to their wild-type littermates (Fig. 2m,n). These results suggest that MBNL1-mediated differentiation is vital to heart and dermal wound healing through myofibroblast formation.

### Fibroblast-specific MBNL1 overexpression induces fibrosis

**Figure 3:**
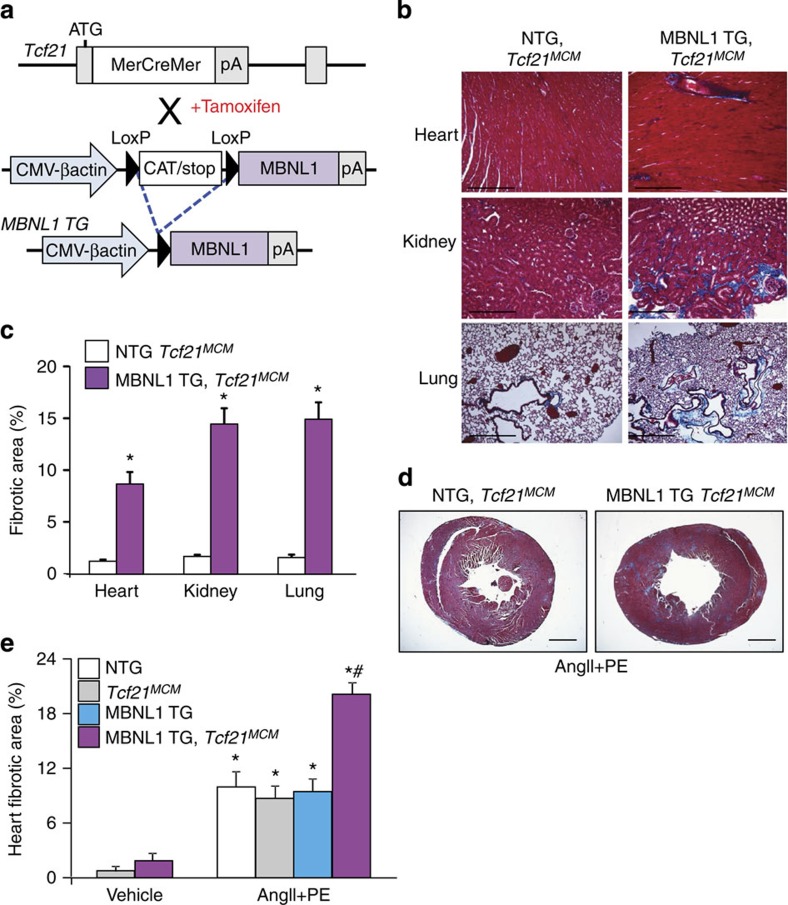
Overexpression of MBNL1 *in vivo* induces fibrosis. (**a**) A schematic depicting two mouse lines: (top) the *Tcf21* locus containing a tamoxifen-regulated MerCreMer (MCM) cDNA and (bottom) a transgene to inducibly express MBNL1 after Cre-mediated excision of a LoxP-CAT-Stop cassette driven by the CMV-βactin promoter. Tamoxifen induces recombination only in cells with *Tcf21* allele expression, which marks quiescent fibroblasts in the heart and many other organs. (**b**,**c**) Masson's trichrome-stained histological pictures and quantification of the area of fibrosis (blue) in the heart, kidney and lung from the indicated mice after 4 months of continuous tamoxifen treatment. Average fibrotic area±s.e.m., *N*=5, *t*-test, *P<0.05 versus NTG, *Tcf21*^*MCM*^. Scale bar, 150 μm. (**d**,**e**) Photographs and quantification of the area of fibrosis (blue) in Masson's trichrome-stained histological heart sections from mice induced with 2 weeks of tamoxifen and then implanted with osmotic mini-pumps that chronically delivered AngII/PE (432 μg kg^−1^ per day/100 mg kg^−1^ per day) for 2 weeks. Average fibrotic area±s.e.m., *N*⩾7, analysis of variance and Newman–Keuls pairwise analysis, *P<0.05 versus vehicle; ^#^*P*<0.05 versus NTG AngII/PE. Scale bar, 500 μm.

Levels of MBNL1 correlate with the degree to which this factor mediates mRNA maturation and alternative splicing *in vivo*^14^,^26^. To specifically address if MBNL1 underlies a myofibroblast differentiation phenotype *in vivo*, we engineered a conditional transgenic (TG) mouse model in which expression of MBNL1 can be increased by Cre-dependent recombination (Fig. 3a). In cultured dermal fibroblasts isolated from conditional MBNL1 TGs, we observed that this LoxP-dependent MBNL1 transgene was effectively induced after adenoviral Cre (AdCre) infection (Supplementary Fig. 2a). AdCre-infected MBNL1 TG dermal fibroblasts also became strongly positive for αSMA stress fibres, whereas fibroblasts infected with the control Adβgal were negative (Supplementary Fig. 2b).

To achieve MBNL1 expression in quiescent fibroblasts *in vivo*, conditional MBNL1 TG mice were mated to mice containing a tamoxifen inducible MerCreMer (MCM) cassette inserted within the *Tcf21* locus (*Tcf21*^*MCM*^, ref. 27). Tamoxifen was given at weaning (Fig. 3a) and 1-month later cardiac fibroblasts were isolated from MBNL1 TG-*Tcf21*^*MCM*^ hearts to determine the level of MBNL1 overexpression (Supplementary Fig. 2c,d). With four additional months of continuous tamoxifen treatment (to prevent a proliferation selection bias), MBNL1 TG-*Tcf21*^*MCM*^ mice developed perivascular fibrosis in the heart, as well as interstitial fibrosis in the kidneys and lung (Fig. 3b). At this time point the heart, kidney and lung were ∼8%, ∼14% and ∼15% fibrotic because of MBNL1 overexpression within the fibroblasts of these organs, respectively (Fig. 3b,c). In comparison, non-TG *Tcf21*^*MCM*^ mice (NTG-*Tcf21*^*MCM*^) had minimal fibrotic staining. These results indicate that induction of MBNL1 expression in quiescent tissue-resident fibroblasts induces a fibrotic response likely by initiating myofibroblast differentiation that mimics the injured state.

Although fibroblast-specific expression of MBNL1 elicited a fibrotic response in multiple tissues that progressed with age, we hypothesized that this response was likely muted given a lack of cytokine and stress-dependent co-stimuli. To examine this hypothesis, MBNL1 TG-*Tcf21*^*MCM*^ and NTG littermates were given 2 weeks of tamoxifen and then subjected to a chronic fibrotic stimulus in which a mixture of AngII and phenylephrine (PE) were delivered by osmotic mini-pump for 2 weeks. Immediately after pump removal, the hearts were excised and fibrosis was determined by Masson's trichrome staining (Fig. 3d,e). The fibrotic area of MBNL1 TG-*Tcf21*^*MCM*^ hearts was doubled in comparison to control littermates that were also treated with AngII/PE (Fig. 3d,e), providing evidence that the overexpression of MBNL1 facilitates fibroblast differentiation and tissue fibrosis.

### MBNL1 regulates RNAs associated with differentiation

To elucidate the potential mechanism whereby MBNL1 programmes myofibroblast differentiation, we analysed the expression of polyadenylated transcripts by total genome RNA sequencing (RNAseq) from *Mbnl1*^*+/+*^ and *Mbnl1*^−/−^ MEFs that were infected with AdMBNL1, Adβgal (negative control) or treated with TGFβ (positive control for myofibroblast differentiation; Supplementary Fig. 3a). Similar to TGFβ treatment of *Mbnl1*^*+/+*^ MEFs, adenoviral overexpression of MBNL1 produced a fivefold increase in MBNL1 expression without impacting expression of MBNL2 or MBNL3, as well as induced expression of key fibroblast-differentiation-specific genes (Supplementary Fig. 3b,c). The mRNAseq analysis revealed that *Mbnl1*^−/−^ MEFs had a profile of reduced differentiation-related genes, independent of TGFβ treatment (Supplementary Fig. 3a). Consistent with the functional implications of these observations in *Mbnl1*^−/−^ MEFs, MBNL1 overexpression in wild-type MEFs induced or increased expression of genes underlying differentiated organ function, organ morphogenesis, Cdc42 GTPase activity, ECM regulation and many other functional categories suggestive of enhanced cellular differentiation (Supplementary Fig. 3a). We were surprised to observe that only a few upregulated gene clusters were shared by both TGFβ-treated and AdMBNL1-infected MEFs, suggesting that MBNL1 and TGFβ treatment rely on a small set of similar mechanisms for enhancing a profile of differentiation-specific gene expression in fibroblasts (Supplementary Fig. 3a).

**Figure 4:**
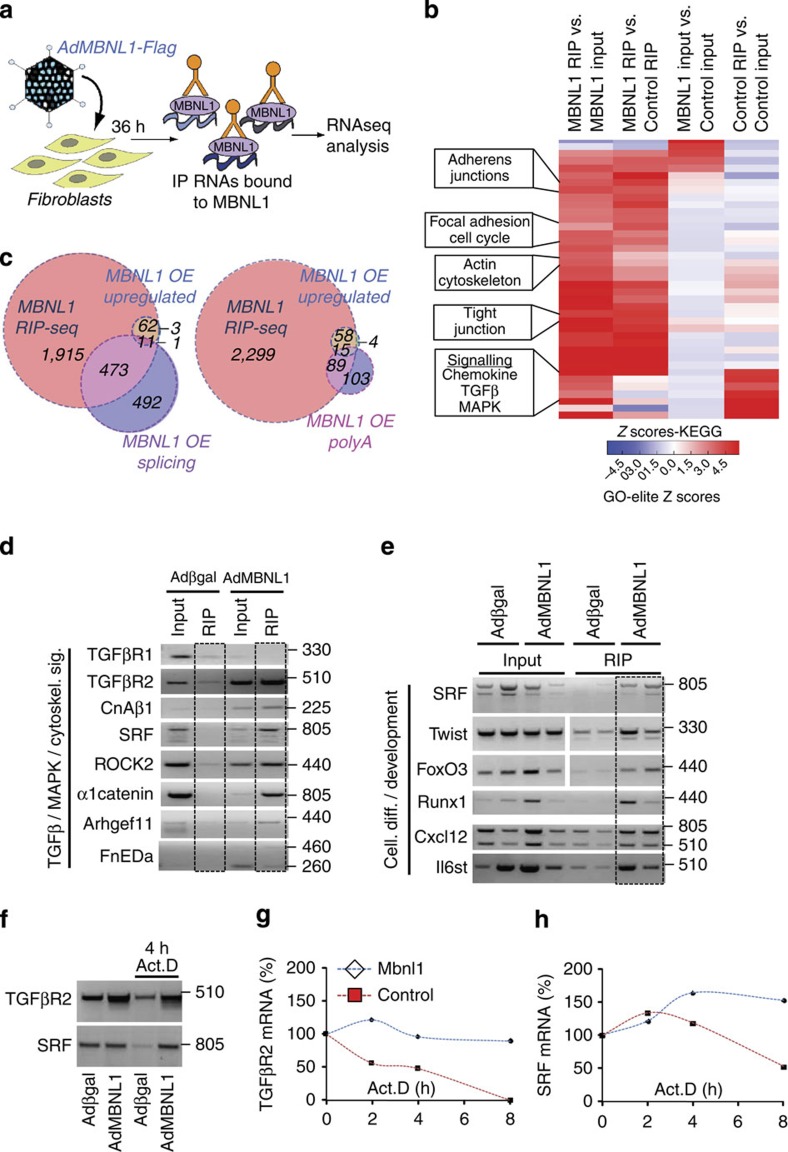
Analysis of MBNL1-bound RNA profiles and expression changes. (**a**) Schematic of RNA immunoprecipitation (RIP) method used to detect all transcripts bound by MBNL1 in activated fibroblasts. (**b**) Heat map derived from the RIP assay depicting enrichment of transcripts bound to MBNL1 with MBNL1 overexpression and control conditions from both the inputs and immunoprecipitated samples. Here the transcripts were clustered into significant KEGG pathways. Four of these pathways are further developed in Supplementary Figs 4–7 using Wikipathways. (**c**) Left, Venn diagram illustrating the overlap between MBNL1-bound RNAs (MBNL1 RIP-Seq), RNAs upregulated in the input from the MBNL1 overexpression condition (MBNL1 OE upregulated) and alternatively spliced RNAs in the input from the MBNL1 overexpression condition (MBNL1 OE splicing). Right, Venn diagram illustrating the overlap between MBNL1-bound RNAs (MBNL1 RIP-Seq), RNAs upregulated in the input from the MBNL1 overexpression condition (MBNL1 OE upregulated) and transcripts with alternative polyadenylation detected in the input from the MBNL1 overexpression condition (MBNL1 OE PolyA). Genes per each subsection are listed in Supplementary Data 1. (**d**,**e**) RT–PCR validation of several transcripts detected in RIP-RNAseq assays that were associated with TGFβ-MAPK-cytoskeletal signalling (cytoskel. Sig.) and cellular differentiation (Cell. diff.) and development KEGG pathways. (**f**) RT–PCR of TGFβR2 and SRF expression from fibroblasts with or without AdMBNL1 infection and with or without actinomycin-D (Act.D) treatment to assess transcript stability. Adβgal infection was used as a control. (**g**,**h**) Same assay as in **f** but over 8 h with 2 h increments. *N*=3 experiments.

To identify RNAs that are directly regulated by MBNL1, RNA immunoprecipitation (RIP) assays were performed as illustrated in Fig. 4a. Rat cardiac fibroblasts were infected with Adβgal (control) or a Flag epitope-tagged AdMBNL1 construct (AdMBNL1-Flag) and Flag antibody resin was used to immunoprecipitate all RNAs that bound to MBNL1, followed by RNAseq analysis. RNA targets with FPKM (fragments per kb of exon per million fragments) values ⩾ 1 in the MBNL1 RIP were selected and ranked by fold enrichment relative to control RIP FPKM scores. In this grouping, there were 2,461 transcripts that bound to MBNL1 that were enriched by 3- to 600-fold and several of them were previously reported as MBNL1-regulatory targets, such as MBNL1, Ptbp1, Fosl2, Ddx6, Pdlim5, Nfix, Hdac5, Usp14, Cnbp, Papola, Dnajb6, Fn1, Bgn, Gtpbp4, Itga6, Ato2a, Fnip1 and CD47 (refs 14, 21, 22, 28). These ∼2,500 transcripts were also subjected to the Kyoto Encyclopedia of Genes and Genomes (KEGG) pathway analysis that identified chemokine signalling, TGFβ signalling, MAPK signalling, actin cytoskeleton, focal adhesion, adheren junctions and cell cycle regulation (Fig. 4b). Several of these pathway clusters including TGFβ signalling (Supplementary Fig. 4), MAPK signalling (Supplementary Fig. 5), actin cytoskeleton regulation (Supplementary Fig. 6) and focal adhesions (Supplementary Fig. 7) have been previously identified as dominant activators of myofibroblast differentiation and each had an enriched number of mRNAs that immunoprecipitated with MBNL1.

In comparing MBNL1-bound transcripts (MBNL1 Rip-Seq) to upregulated genes from the input (MBNL1 OE Upregulated), only 77 genes were overexpressed, but remarkably all except three of those genes are bound by MBNL1 (Fig. 4c and Supplementary Data 1). We also analysed the MBNL1 RIP for alternative splice variants and found 965 genes in the MBNL1 input (MBNL1 OE Alternative Splicing) but only ∼20% of bound MBNL1 transcripts were alternatively spliced (MBNL1 Rip-Seq, Fig. 4c, left panel, and Supplementary Data 1). Given MBNL1's role in binding 3′ UTRs^17^ and regulating alternative polyadenylation sites^26^ the overlap between MBNL1-bound transcripts and those with alternative polyadenylation was also examined (Fig. 4c, right panel, and Supplementary Data 1). Out of 192 transcripts that had alternative polyadenylation sites in the MBNL1 input (MBNL1 OE upregulated Alternative PolyA), MBNL1 bound half of them. Interestingly 8% of the genes with alternative polyadenylation were also upregulated and all bound to MBNL1 (Fig. 4c, right panel) suggesting a potential transcript stability component to MBNL1-dependent transcript maturation^17^,^26^. Bioinformatics analysis was used to compare the MBNL1-bound targets from our RIPseq analysis against published MBNL1-binding sites by CLIPseq, which was filtered by binding site location (3′UTR, intronic or exonic, Supplementary Table 1). This analysis showed that ∼17% of the MBNL1 messages (with known binding sites) are intronic, whereas 40% are in exons and 35% are in the 3′UTRs indicating that there is a higher probability of MBNL1 binding to spliced RNAs rather than being a splicing effector.

### Validation of MBNL1 target RNAs in myofibroblasts

The RIP data identified mRNA targets corresponding to several signalling nodes that positively influence myofibroblast differentiation (Fig. 4b and Supplementary Figs 4-7). We used reverse transcriptase–PCR (RT–PCR) to validate individual transcripts from these nodal pathway regulators, such as calcineurin Aβ (CnAβ), SRF and the TGFβ receptor 2 (TGFβR2; Fig. 4d and Supplementary Table 2). TGFβ receptor 1 (TGFβR1) was not regulated by MBNL1 in our RIP or RNAseq analysis and that was reconfirmed by RT–PCR (Fig. 4d and Supplementary Table 2). In addition, MBNL1 immunoprecipitated several mRNAs that were identified by RNAseq involved in actin cytoskeletal assembly and focal adhesions including Rho-associated coiled–coil containing protein kinase 2 (Rock2), catenin-cadherin-associated protein-alpha 1 (α1catenin) and several Rho guanine nucleotide exchange factors (Arhgef11 *shown here*) as well as SRF, which were all reconfirmed here (Fig. 4d and Supplementary Table 2). These observations are also consistent with previous work demonstrating that Rho-signalling and the cytoskeleton are major pathways affected by MBNL1-mediated polyadenylation^26^. MBNL1 can also directly bind fibronectin (*Fn1*) as well as the splice variant (FnEDa) characteristic of myofibroblast transformation^14^, which was recapitulated here (Fig. 4d and Supplementary Table 2). In performing a cross analysis of our RIPseq data with published CLIPseq data (Supplementary Table 1), we observed a conserved binding site in fibronectin's 3′UTR suggesting that MBNL1 likely modulates all fibronectin message variants equally.

MBNL1 RIP target RNAs were also analysed by Gene Ontology biological networks and there was a clear enrichment in differentiation and developmental processes similar to the total RNAseq gene expression profile described earlier. These processes included vasculature development, regulation of development, cardiovascular system development, regulation of cell migration, tube development and epithelial-to-mesenchymal transition, in addition to strong enrichment in signatures of late mesoderm-associated genes. These MBNL1-bound transcripts included SRF, Twist1, FoxO3, Runx1, Cxcl12 and Il6st, which were all validated and quantified by RT–PCR here (Fig. 4e and Supplementary Table 2).

We have previously shown the requirement for TGFβR2, SRF and CnAβ in fibroblast to myofibroblast differentiation^4^, although here we investigated the regulation of these transcripts directly by MBNL1. CnAβ is alternatively spliced to a known variant, CnAβ1 (Fig. 4d), that was previously shown to be constitutively active^29^. TGFβR2 and SRF transcripts were both bound by MBNL1 and upregulated (Fig. 4d), which we hypothesized was due to increased message stability. To test this hypothesis directly, cardiac fibroblasts were treated for 4 h with the transcription-inhibitor actinomycin-D (Act.D), and then RT–PCR was performed to measure transcript decay relative to untreated fibroblasts (Fig. 4f). In fibroblasts infected with the Adβgal control, Act.D caused both TGFβR2 and SRF transcript levels to decay (Fig. 4f). By contrast, TGFβR2 and SRF transcript levels were maintained in fibroblasts overexpressing MBNL1 with Act.D, suggesting that MBNL1 was indeed stabilizing these transcripts (Fig. 4f). Indeed, an Act.D time-course experiment over 8 h again showed that both TGFβR2 and SRF transcripts decayed under control conditions but that MBNL1 overexpression maintained these levels (Fig. 4g,h). Together, these data demonstrate that MBNL1-mediated RNA stabilization can selectively regulate transcripts that have a pro-differentiation function in myofibroblasts.

### MBNL1 controls CnAβ mRNA in myofibroblast differentiation

**Figure 5:**
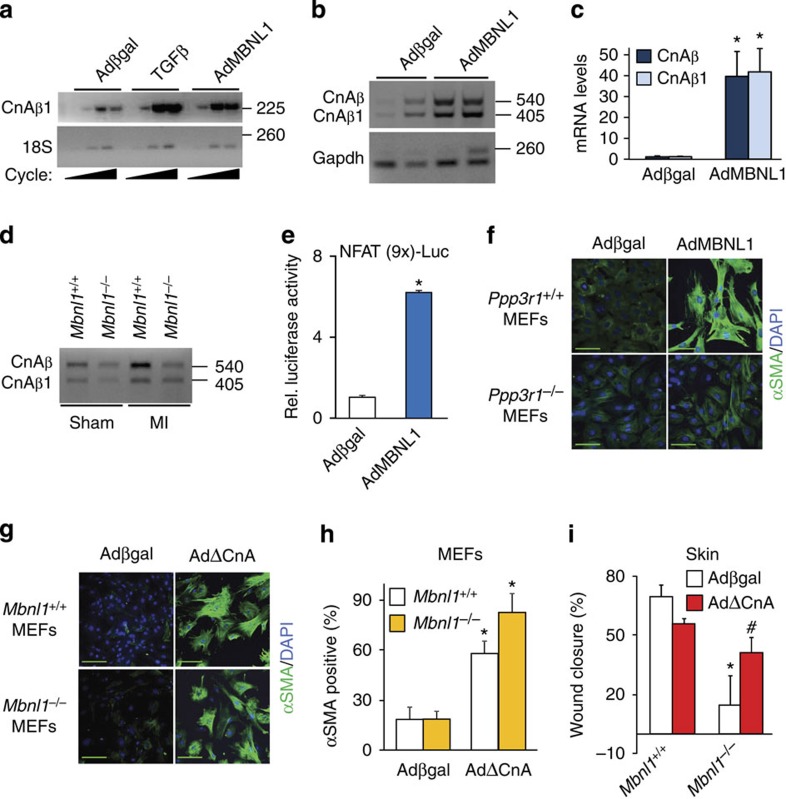
MBNL1-regulated calcineurin Aβ mRNA levels underlies myofibroblast differentiation. (**a**) RT–PCR of a calcineurin Aβ splice variant 1 (CnAβ1) from Adβgal (control), TGFβ-treated and AdMBNL1-infected fibroblasts. 18S RNA expression was used for normalization. (**b**,**c**) RT–PCR gel and quantification of the two major calcineurin Aβ-spliced isoforms, CnAβ and CnAβ1, from Adβgal- or AdMBNL1-infected fibroblasts, with Gapdh as a processing and loading control. *N*=3 experiments, *t*-test, *P<0.05 versus Adβgal. (**d**) RT–PCR analysis of the same two calcineurin Aβ spliced isoforms from fibroblasts of hearts subjected to sham or MI injury in *Mbnl1*^*+/+*^ and *Mbnl1*^−/−^ mice. *N*=3 experiments. (**e**) NFAT-luciferase reporter activity in Adβgal- and AdMBNL1-infected fibroblasts. Average luciferase activity is expressed relative (Rel.) to Adβgal (control), *N*=3, *t*-test, *P<0.05 versus Adβgal. (**f**) Immunofluorescence for αSMA (green) stress fibres and nuclei (blue) in *Ppp3r1*^+/+^ and *Ppp3r1*^−/−^ (calcineurin B1 protein) primary MEFs infected with Adβgal or AdMBNL1. (**g**,**h**) Immunofluorescent staining and quantification of αSMA (green) stress fibres and nuclei (blue) in *Mbnl1*^*+/+*^ or *Mbnl*^−/−^ MEFs infected with Adβgal or constitutively active calcineurin (AdΔCnA). Data represent the average number of fibroblasts with αSMA^+^ stress fibres per total nuclei±s.e.m., *N*⩾375, analysis of variance (ANOVA) and Newman–Keuls pairwise analysis, *P<0.05 versus Adβgal. (**i**) Quantification of skin wound closure at 6 days after 6 mm biopsy injury in *Mbnl1*^+/+^ and *Mbnl1*^−/−^ mice treated with either Adβgal or AdΔCnA directly applied throughout the wound. Average closure±s.e.m., *N*⩾14 mice per group, ANOVA and Newman–Keuls pairwise analysis, *P<0.05 versus Adβgal *Mbnl1*^*+/+*^; ^#^*P*<0.05 versus Adβgal *Mbnl1*^−/−^. All scale bars, 50 μm.

As discussed above, RNAseq profiling and RIP identified CnAβ and an alternatively spliced variant CnAβ1 as MBNL1 targets in differentiated fibroblasts, suggesting that calcineurin might be a prominent mechanism for MBNL1-mediated myofibroblast differentiation. Cardiac fibroblasts treated with TGFβ or infected with AdMBNL1 have increased expression of the constitutively active spliced CnAβ1 isoform by RT–PCR (Fig. 5a), but also a nearly equivalent increase in mRNA levels of the native CnAβ spliced form (Fig. 5b,c). This increase in CnAβ mRNA-spliced forms was also observed in cardiac fibroblasts taken from wild-type mouse hearts after MI injury, but interestingly, this increase did not occur from *Mbnl1*^−/−^ mice after MI (Fig. 5d). As another read-out for calcineurin activity, we investigated nuclear factor of activated T cells (NFAT) transcriptional responsiveness in MBNL1 overexpressing versus control fibroblasts (Adβgal), which showed a sixfold induction with AdMBNL1 (Fig. 5e). Moreover, AdMBNL1 infection in *Ppp3r1* (calcineurin B1) null MEFs, which are devoid of all calcineurin function^30^, were unable to induce myofibroblast differentiation (Fig. 5f). In a converse experiment, overexpression of a constitutively active calcineurin mutant (AdΔCnA^31^) in *Mbnl1*^−/−^ MEFs transformed ∼70% of the fibroblast population into myofibroblasts similar to the level of conversion achieved in *Mbnl1*^*+/+*^ MEFs, demonstrating that calcineurin functions downstream of MBNL1 to promote myofibroblast differentiation (Fig. 5g,h). Finally, we also examined this hypothesis *in vivo* by examining if activated calcineurin can rescue the dysfunctional wound healing observed in *Mbnl1*^−/−^ mice (Fig. 2j,k). Here the adenovirus carrying ΔCnA was topically applied to one of two skin wounds, whereas the contralateral wound received an innocuous Adβgal control adenovirus, immediately after the biopsy^4^. Six days later, AdΔCnA treatment significantly accelerated the rate of wound closure in *Mbnl1*^−/−^ mice, whereas the Adβgal-treated wounds still had defective closure rates (Fig. 5i). Collectively, these data demonstrate that maturation of calcineurin mRNAs by MBNL1 is at least one key mechanism for myofibroblast differentiation.

### MBNL1 regulates SRF mRNA in myofibroblast differentiation

**Figure 6:**
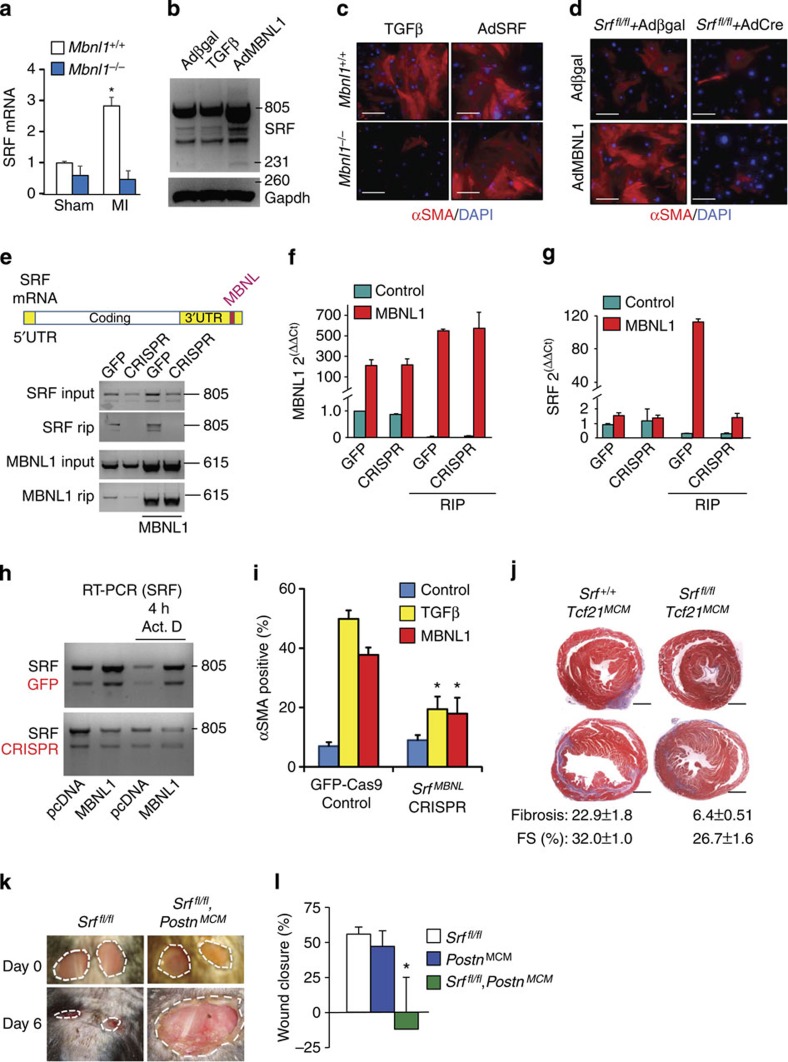
MBNL1-regulated SRF mRNA stability underlies myofibroblast differentiation. (**a**) RT–PCR for SRF mRNA from fibroblasts of hearts subjected to sham or MI injury in *Mbnl1*^*+/+*^ and *Mbnl1*^−/−^ mice. *N*=3 hearts and results are±s.e.m., analysis of variance (ANOVA) and Newman–Keuls pairwise analysis, *P<0.05 versus Sham. (**b**) RT–PCR of SRF and known splice variants in Adβgal (control), TGFβ-treated and AdMBNL1-infected fibroblasts. Gapdh gene expression was a control. (**c**) Immunofluorescence for αSMA (red) stress fibres and nuclei (blue) in *Mbnl1*^*+/+*^ or *Mbnl1*^−/−^ MEFs treated with TGFβ or infected with AdSRF. (**d**) Immunofluorescence for αSMA (red) and nuclei (blue) in *Srf*^*fl/fl*^*+*Adβgal or *Srf*^*fl/fl*^*+*AdCre dermal fibroblasts infected with either Adβgal (control) or AdMBNL1. (**e**) Top, diagram of SRF mRNA showing the mutated MBNL-binding site in the 3′UTR region engineered into a stable fibroblast cell line. Below, RIP assay for MBNL1 binding to either the SRF or MBNL1 mRNA from cell lines that are control (GFP) or CRISPR-mediated deletion of the MBNL1-binding site in SRF. An MBNL1-encoding plasmid was also transfected into cells used to make lanes 3 and 4. (**f**,**g**) RT–PCR validation of SRF and MBNL1 transcript expression visualized in **e**. The data represent the average expression relative to GFP (control) input ± s.e.m., *N*=3 independent experiments. (**h**) RT–PCR for SRF mRNA from the same cell lines described in **e**. Cells were either transfected with a control (pcDNA) vector or one expressing MBNL1 in the presence or absence of actinomycin-D (Act. D) for 4 h. (**i**) Quantification of αSMA expression to indicate myofibroblast differentiation in control (GFP-Cas9) or the *Srf*^*MBNL*^ CRISPR-deleted fibroblasts, with either TGFβ stimulation or MBNL1 overexpression. Data represent the average number of fibroblasts with αSMA^+^ stress fibres relative to the total number of nuclei±s.e.m., *N*⩾800, ANOVA and Newman–Keuls pairwise analysis, *P<0.05 versus the GFP-Cas9 control line. (**j**) Heart histological cross-sections stained with Masson's trichrome to show fibrosis (blue) from the indicated mice 10 days after a ‘small' MI injury. The fibrosis percentage and ventricular fractional shortening (FS) percentage is shown from the two groups of mice, *N*=6–7 in each group. Scale bar, 1 mm. (**k**) Photographs of two skin punch biopsies made on the backs of the indicated adult mice at days 0 and 6 after injury. Photograph scaling can be inferred from day 0, as the biopsy is 6 mm across. (**l**) Quantification of dermal would closure over time measured relative to initial wound size. Average closure±s.e.m., *N*=10, ANOVA and Newman–Keuls pairwise analysis, *P<0.05 versus *Postn*^*MCM*^ or *Srf*^*fl/fl*^. All scale bars unless otherwise noted=50 μm.

The RNAseq profiling and RIP also identified SRF as an MBNL1 target. Remarkably, SRF mRNA levels are induced in cardiac fibroblasts from wild-type mice after MI injury, but not in *Mbnl1*^−/−^ mice, suggesting that MBNL1 can regulate SRF mRNA during a disease insult (Fig. 6a). Indeed, AdMBNL1 infection in cardiac fibroblasts increased the expression of all known SRF splice variants as determined by RT–PCR (Fig. 6b). Previous work has defined SRF as a nodal regulator of myofibroblast differentiation that receives upstream signalling information from Rho and the actin cytoskeleton, as well as non-canonical TGFβ signalling through p38 (refs 4, 32, 33, 34). Thus, we examined if enhanced SRF expression could rescue the impaired capacity of *Mbnl1*^−/−^ MEFs to differentiate. As shown in Fig. 1e, *Mbnl1*^−/−^ MEFs are refractory to TGFβ-induced myofibroblast differentiation, but this defect was rescued to wild-type levels by Adenovirus encoding SRF (AdSRF) infection (Fig. 6c). In the converse experiment, MEFs devoid of SRF (*Srf*^fl/fl^+AdCre) were refractory to MBNL1-mediated myofibroblast differentiation when compared with MEFs replete with SRF (*Srf*^fl/fl^+ Adβgal), suggesting that SRF is also a critical downstream mediator of MBNL1-directed differentiation, and that the ability of MBNL1 to augment SRF transcript stability is another critical mechanism for myofibroblast differentiation regulated through MBNL1 (Fig. 6d).

Recent evidence has suggested that 60% of MBNL1 binding is in a transcript's 3′UTR^17^ where it can regulate transcript stability and alternative polyadenylation^26^. To probe more deeply into the mechanism whereby MBNL1 regulates myofibroblast differentiation through SRF, we used clustered regularly interspaced short palindromic (CRISPR) technology to generate a fibroblast cell line in which the 3′ UTR encoding region of the *Srf* gene was mutated so that the transcript no longer bound MBNL1. RBPmap software was first used (see Methods) to map potential MBNL1-binding sites in the 3′UTR of SRF, followed by CRISPR-Cas9 gene editing to delete putative sites (*Srf*^*MBNL*^ CRISPR). A control cell line that contains an innocuous green fluorescent protein (GFP) cassette with Cas9 was also used for comparison. Of the predicted binding sites, five of them had sequences consistent with those identified as preferred MBNL1 targets^35^. Therefore, we chose to individually mutate each of these sites and found that only one worked exceptional well (Fig. 6e, upper diagram). Remarkably, RIP analysis from the CRISPR mutated fibroblast cell line, transfected with MBNL1, showed a loss in MBNL1 binding to the SRF transcript when the critical 3′UTR MBNL-binding site in the *Srf* gene was mutated, compared with a Cas9-GFP control cell line that had a wild-type *Srf* gene (Fig. 6e–g, bottom panel). As a control, MBNL1 binding to its own mRNA (this is known to occur) was not changed in these same samples. Mutation of this MBNL1-binding site in the *Srf* 3′UTR also reduced SRF mRNA levels at baseline (see SRF input lanes, Fig. 6e), and because this result is consistent with a trend towards reduced SRF mRNA in *Mbnl1*^−/−^ cardiac fibroblasts (Fig. 6a), we interpret this reduction to reflect an autonomous role for MBNL1 in regulating SRF mRNA stability and not some other destabilizing effect of the mutation. In an independent experiment, SRF expression again showed a reduction in the *Srf*^*MBNL*^ CRISPR cell line at baseline when compared GFP-Cas9 control cell line at time 0 (Fig. 6h). After 4 h of inhibiting transcription with Act.D, MBNL1 stabilized the SRF transcript in control fibroblasts but not in *Srf*^*MBNL*^ CRISPR mutant cell line with MBNL1 overexpression, suggesting that disruption of the MBNL1-binding site in SRF's 3′UTR prevented MBNL1-mediated stabilization of this transcript (Fig. 6h). As a functional readout, ablating this MBNL1-binding site in the endogenous *Srf* gene reduced both MBNL1- and TGFβ-mediated myofibroblast transformation of this cell line by approximately half (*Srf*^*MBNL*^ CRISPR+MBNL1 or TGFβ) when compared with control fibroblasts (GFP-Cas9; Fig. 6i).

To examine the potential importance of SRF in mediating the cardiac fibrotic response after MI injury or with skin wounding *in vivo*, we used *Srf-LoxP*-targeted mice crossed with either the *Tcf21*^*MCM*^ or the *Postn*^*MCM*^ knock-in alleles. Deletion of *Srf* from cardiac fibroblasts with *Tcf21*^*MCM*^ resulted in 100% lethality following our standard MI injury model, because of ventricular wall rupture from a lack of proper fibrosis and scar formation. Hence, we employed a modified MI procedure in which the left coronary artery was ligated further down the heart resulting in smaller infarctions that did not rupture as frequently in the *Srf*^*Tcf21-MCM*^ mice (Fig. 6j). Under these conditions, *Srf*^*Tcf21-MCM*^ mice showed almost a fourfold reduction in scar size and ensuing fibrosis over 10 days compared with control mice, as well as a significantly worse profile of ventricular fractional shortening (Fig. 6j). Because TCF21 is not expressed in skin fibroblasts, we switched to a *Postn*^*MCM*^ knock-in allele-containing mouse, which expresses the same tamoxifen inducible Cre protein (MerCreMer, MCM). Loss of *Srf* from activated skin fibroblasts not only prevented wound healing over 6 days, but also it caused wound rupture and expansion (Fig. 6k,l). Thus, SRF activity in fibroblasts is critical for wound healing and fibrosis *in vivo*.

## Disscussion

**Figure 7:**
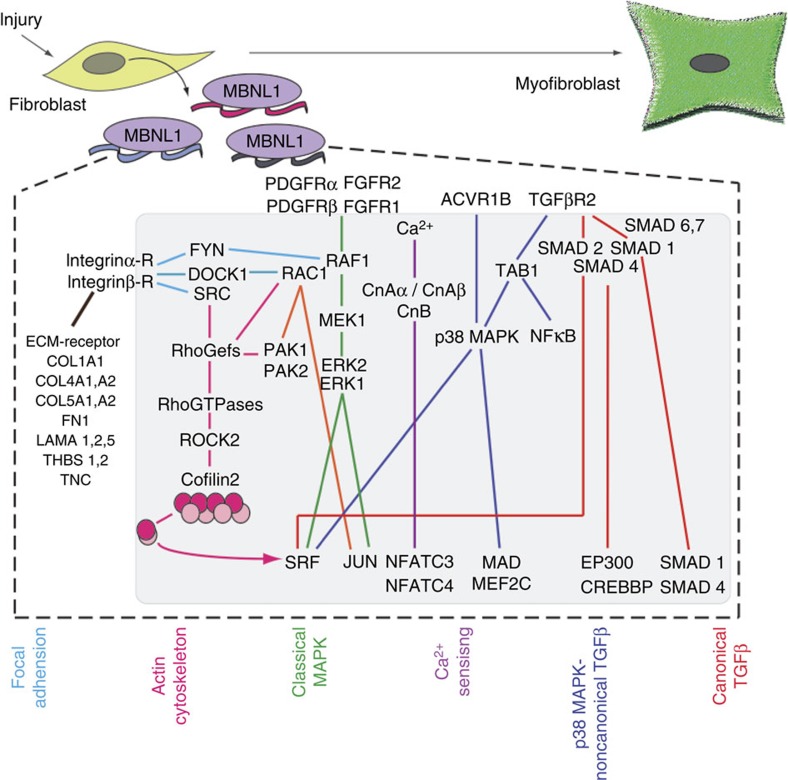
MBNL-regulated signalling pathways underlying myofibroblast differentiation. Based on the RIP data presented earlier, all of the depicted genes are direct MBNL1 targets and are also nodal factors within multiple pathways that underlie myofibroblast differentiation signalling. The following are the full names for the depicted factors: Integrin α/β-R (integrinα/β receptor family); COL1A1 (collagen type 1,α1); COL4A1 and A2 (collagen type 4,α1 and α2), COL5A1,A2 (collagen type 5, α1 and α2); FN1 (fibronectin 1); LAMA 1,2,5 (laminin α1, α2 and α5); THBS1,2 (thrombospondin 1 and 2), TNC (tenascin C), FYN (Src family kinase); DOCK1 (dedicator of cytokinesis 1); SRC (SRC Proto-oncogene); PDGFRα/β (platelet-derived growth factor receptor α and β); FGFR1/2 (fibroblast growth factor receptor 1 and 2); RAF1 (Raf1 Proto-oncogene, MAP3K); MEK1 (mitogen-activated protein kinase kinase 1); ERK1/2 (extracellular signal-regulated kinase 1 and 2); JUN (Jun Proto oncogene); CnAα/CnAβ (calcineurin Aα and Aβ); CnB (calcineurin B); NFATC3/C4 (nuclear factor of activated T cells 3 and 4); ACVR1B (activin A receptor 1B); p38 MAPK (mitogen-activated protein kinase 14); MAD (MAX dimerization protein 1); MEF2C (myocyte enhancer factor 2C); TAB1 (TGFβ-activated kinase binding protein 1); NFκB (nuclear factor of κ light polypeptide gene enhancer in B-cells); EP300 (E1A-binding protein p300); CREBBP (CREB-binding protein).

The results of this study identified a new regulatory mechanism underlying myofibroblast differentiation/mesenchymal transition, whereby MBNL1 promotes the maturation of selective transcripts that serve as integral nodes in the molecular pathways orchestrating myofibroblast differentiation and wound healing. MBNL1 expression, which is normally low in uninjured tissue and quiescent fibroblasts, becomes induced in fibroblasts in response to signals from the injured environment. With upregulation, MBNL1 then binds thousands of transcripts, most of which are easily parsed into selected signalling pathways that are consistent with myofibroblast differentiation, or differentiation in general. These pathways include canonical TGFβ signalling, non-canonical TGFβ signalling through p38 MAPK, classical MAPK signalling, Ca^2+^-calcineurin-NFAT signalling, actin cytoskeletal regulation (focused on Rho GTPase-ROCK-SRF) and focal adhesion regulation (Fig. 7). Importantly, Fig. 7 only shows genes from the discussed pathways that are specifically bound by MBNL1 in our RIPseq data.

As a direct mechanism, we determined that MBNL1 could bind the target RNAs CnAβ and SRF in augmenting myofibroblast differentiation. Indeed, we have previously shown that calcineurin activity is required for myofibroblast differentiation and for *in vivo* cardiac and dermal wound healing^4^. MBNL1 also increased the expression of the transcription factor SRF, a primary initiator and nodal regulator of myofibroblast gene expression^4^,^32^,^33^,^34^. Mechanistically, we were able to show that MBNL1 directly bound to the SRF transcript and that site-directed mutagenesis of this binding site within a fibroblast cell line reduced the ability of MBNL1 to augment endogenous SRF expression and subsequent myofibroblast differentiation.

Bioinformatic analysis of MBNL1-bound transcripts also revealed a clustering of genes associated with the ECM, focal adhesions, collagens, fibronectin, integrins and other differentiation-regulating pathways. Although not directly tested, one could envision that greater translation of these transcripts would be paramount to scar formation and retaining tissue integrity during wound healing. There is a growing body of evidence implicating MBNL1 as a master regulator of cellular differentiation through its ability to regulate select RNA transcripts during developmental transitions in skeletal and cardiac muscle^18^,^19^,^20^,^36^, pluripotency of embryonic stem cells^21^,^22^ and now fibroblast to myofibroblast transformation. Specifically, during cardiac development, MBNL1 promotes alternative splicing that is critical for facilitating fetal to adult isoform transitions for several Ca^2+^ handling, contractile and ECM proteins^20^,^37^. These protein isoform transitions are vital for enhancing the differentiated state of the myocyte towards accommodating the increased workload demands of the adult heart^20^. Similarly, in skeletal muscle, several known MBNL1 targets encode for critical structural and contractile proteins, and when MBNL1 function is lost many of those messages are inappropriately spliced or degraded resulting in muscle pathology and myotonic dystrophy^16^,^18^,^23^,^37^.

Although MBNL1 functions as a global regulator of differentiation in general, our observations in myofibroblast transformation suggest that it works with those pre-existing transcripts already determined by tissue-specific commitment factors that act above the level of MBNL1. For example, in cardiac muscle transcription factors such as GATA4 and Nkx2.5 specify the cardiac lineage and the start of differentiation-specific gene expression, which is then more globally integrated and enhanced by MBNL1 (ref. 19). Similar paradigms exist in skeletal muscle through the myogenic regulators of the MyoD family that determine cellular commitment and the initiation of differentiation, but that are then enhanced by MBNL1 to promote more complete differentiation for adult tissue function^24^,^38^,^39^.

Until now, the role of RNA-binding proteins in regulating the fibrotic phase of the wound-healing response through myofibroblast differentiation has not been considered, but it represents a mechanism for inducing a rapid and global response through simultaneously enhancing the activity of several nodal signalling components (Fig. 7). Certainly, the data presented here demonstrates that MBNL1's regulation of the transcriptome is an integral mechanism for how a fibroblast responds rapidly to injury by transitioning to the highly differentiated cell type known as the myofibroblast, which has the new features of both a contractile muscle cell and secretory fibroblast. As MBNL1 simultaneously regulates multiple arms of the myofibroblast differentiation signalling network and would be amenable to current RNA-based therapeutic strategies, it represents a novel target for either accelerating the acute wound-healing response and/or limiting the chronic phase that results in fibrosis and scarring during disease.

## Methods

### Mouse models, dermal wound healing and MI injury

*Mbnl1*^−/−^ (sv129 congenic strain^16^) and *Tcf21*^*MCM*^ (C57BL/6-sv129) mice^27^ were previously described. *Postn*^*MCM*^ (C57BL/6-sv129) mice were generated by inserting the MerCreMer cDNA cassette into the first coding exon of the *Postn* gene by standard homologous recombination in embryonic stem cells, followed by generation of germline-targeted mice. *Srf-LoxP, CD-1 background*-targeted mice were described previously^40^. MBNL1 TG mice (C57BL6) were engineered by cloning the human MBNL1 cDNA downstream variant 1 cDNA (Accession number BC043493) into a LoxP-flanked chloramphenicol (CAT)-polyA stop sequence cassette (Fig. 3a). Cre expression from the inducible *Tcf21*^*MCM*^ knock-in allele was activated by feeding the mice tamoxifen citrate chow (400 mg kg^−1^, Harlan Laboratories) for at least 2–4 weeks unless otherwise stated. For the dermal wound-healing model, fully anaesthetized mice received two dorsal 6 mm excision punch biopsies, and the wounds were subsequently assessed for size and health at the time of injury for up to 6 days^4^. For some experiments, the mice were biopsied and AdΔCnA was topically applied to one of the wounds and Adβgal was applied to the contralateral wound as a control. All animal experimentation included both sexes. Myofibroblasts were identified in wounded skin by antigen retrieval and staining with a combination of αSMA antibody (1:500, mouse monoclonal, Clone 1A4, Sigma) and isolectin-B4 (Vector Biolabs) for epithelial cell identification^4^. For MI, the left coronary artery of mice was permanently ligated^31^,^41^, and M-mode echocardiography was used to assess ventricular geometry and function 1 week after surgery. All experimentation on mice followed the appropriate analgesic and anaesthesia guidelines and was approved by the Institutional Animal Care and Use Committee of Cincinnati Children's Hospital Medical Center. The ages and numbers of mice used are stated in the results or figure legends.

### Cell culture, differentiation and proliferation assays

Cardiac fibroblasts were isolated from ventricular tissue that was obtained from P1-P2 neonatal rats. The ventricles were enzymatic dissociated with 0.05% pancreatin and 84 U ml^−1^ of collagenase. Fibroblasts were isolated by allowing the total cell mix from the hearts to settle by gravity for 10 min. The supernatant was centrifuged for 5 min at 1,000*g*, resuspended in DMEM with 10% fetal bovine serum (FBS), and then plated on gelatin-coated dishes for primary cell culture. A minimum of three passages was used to achieve a pure fibroblast population^42^. The simian virus 40 (SV40)-transformed *Mbnl1*^*+/+*^*, Mbnl*^−/−^ (ref. 26), LoxP-targeted *Srf (Srf*^*fl/fl*^ (ref. 40)), *Ppp3r1*^*+/+*^ and *Ppp3r1*^−/−^ (ref. 30) MEFs were all previously described.

Primary dermal fibroblast were isolated by enzymatic digestion of mouse skin explants^4^. Briefly, mice were anaesthetized, the skin shaved and ∼40 × 15 mm^2^ section of the dorsal skin was dissected free down to the muscle layer. The skin explant was digested in 0.25% trypsin overnight at 4 °C. The skin was then minced and digested for 2–4 h in collagenase type I at 37 °C. The skin was triturated and resuspended in DMEM with 10% FBS. Cell lines were not tested for mycoplasma.

Myofibroblast transformation was induced using recombinant porcine TGFβ (10 ng ml^−1^, R&D System), AngII (100 nM, Sigma) or adenoviral gene transfer and analysed 48 h later by immunocytochemistry for αSMA stress fibres (1:1,000, mouse monoclonal antibody, Clone 1A4, Sigma) and gene expression as described previously^4^. Here fibroblasts were fixed overnight at 4 °C in 4% paraformaldehyde, washed in PBS and blocked in PBS containing 0.5% Triton X-100 and 10% normal goat serum. Primary and secondary antibodies were diluted in PBS containing 0.5% Triton X-100 and 2% normal goat serum, and fibroblasts were incubated with primary antibody for 1.5 h at room temperature.

Fibroblast contractile activity was also assessed by collagen contraction assays in which 40,000 fibroblasts are seeded into a collagen gel matrix and contraction of the gel was measured 48 h later^4^. MTT (3-(4,5-dimethyllthiazol)-2,5-diphenyl tetrasodium bromide) colorimetric assay (Millipore) was used to quantify the proliferative capacity of *Mbnl1*^*+/+*^ and *Mbnl1*^−/−^ MEFs^4^. For the MTT assay, a standard curve is generated by seeding wells with known amounts of fibroblasts and measuring the corresponding optical density (550 nm). Linear regression is then performed on the standard curve and the equation is used to estimate the cell number in each of the experimental wells. In all assays, quiescent fibroblasts are those plated and grown in culture in the absence of treatment with any agonist or gene transfer reagents. Fibroblasts were maintained in DMEM with 10% FBS and passaged at 70% confluence. Twenty-four hours before all experimentation the serum was reduced to 2% to lower their intrinsic activation profile. MBNL1-contraining or an empty pcDNA (control) plasmid were transfected into the stable cell lines to induce myofibroblast differentiation and measure SRF transcript stability.

### Adenoviral gene transfer

For the MBNL1 adenoviral vector, a murine MBNL1 cDNA was cloned into the pShuttle vector with and without a 3′ Flag epitope, and transfected into HEK293 cells using the AdEasy adenoviral production kit (Agilent Technologies). The recombinant adenovirus was plaque purified, expanded and cesium chloride gradients were used to purify and concentrate the virus^43^. Typically, fibroblasts were infected with adenovirus (100 multiplicity of infection) diluted in DMEM with 2% FBS, incubated overnight and then the media were exchanged. Some experiments and adenoviruses were previously published, including: ΔCnA, βgal, GFP, Cre recombinase, SRF and NFAT(9X)-luciferase reporter^4^,^31^,^43^,^44^.

### Genome-wide gain-of-function screen and luciferase assays

The Mammalian Genome Collection library, which contains ∼18,400 human and rodent cDNAs, was seeded with one clone per well, 40 ng each, in a 384-well format (Scripps Institute, Florida, Cell-based Screening Core). This library was co-transfected (Mirus, Trans-IT) with an equal amount of αSMA-luciferase reporter construct into SV40-transformed MEFs and luciferase activity was assessed 2 days after transfection^4^. Clones were selected if they induced a luciferase signal greater than or equal to that of recombinant TGFβ. A periostin (*Postn*)-luciferase promoter reporter was also used to confirm activity^4^. Here ∼2,200 kb of the *Postn* promoter was cloned upstream of luciferase (pGL3, Promega). This construct was co-transfected (Mirus, Trans-IT) into fibroblasts with either an MBNL1 or control (pcDNA) plasmid and treated with vehicle in media or recombinant TGFβ (10 ng ml^−1^). Luciferase activity was measured 48 h post transfection.

### Reverse transcriptase–PCR

For fibroblast experiments, RNA was isolated and prepared with an RNAeasy kit (Qiagen), and for skin wounds, RNA was prepared using *TRIzol* extraction. Total RNA was reverse transcribed using Superscript III first-strand synthesis kit (Invitrogen). The primer sequences used for all PCR reactions are as follows: IL6st: 5′- CAGATCGAGCAGAATGTGTATGGAATC-3′ , 5′- ATTGGATGTCCGACTTCAGCC-3′ ; Runx1: 5′- CGAGATTCAACGACCTCAGGTT-3′ , 5′- ACAGGAGGCGAGTAGGTGAA-3′ ; Cxcl12: 5′- CCAACGTCAAACATCTGAAAATCCTCA-3′ , 5′- GTGGAAGGTTGCTACTCCCC-3′ ; FnEDa: 5′- CCCACCGTGGAGTATGTGG-3′ , 5′- AGCCCTGACACAATCACGGA-3′ ; Rock2: 5′- ATGCAGATTCACTTGTAGGAACCTACAG-3′ , 5′- TGTTCCAGTTCCTCCTTGGA-3′ ; SRF: 5′- GGGAAACCAAGGACACACTGA-3′ , 5′- ATAAGTGGTGCCGTCCCTTG-3′ ; α1Catenin: 5′- CGACTTCACCCGAGGCAAA-3′ , 5′- CCCCACAGTAGGGCTAAAGG-3′ ; Arhgef11: 5′- CACGCTACGGGGAACTGTTT-3′ , 5′- CTGTCTTCCAGGGCGATGTC-3′ ; Twist1: 5′- GTCGCTGAACGAGGCATTTG-3′ , 5′- GACATCTAGGTCTCCGGCCT-3′ ; FoxO3: 5′- TCATCTGTTTGTACCCGTCGAA-3′ , 5′- CTCTTGCTCTCTCCTCTCGC-3′ ; TGFβR1: 5′- TGGCAGAGCTGTGAGGCCTTGA-3′ , 5′- CCCTAAGGGCTGCCGGTCGT-3′ ; TGFβR2: 5′- CAGGGGTGCTCCACT-3′ , 5′- CCAACAGCGGGCAGGTGGGA-3′ ; CnAβ: 5′- GATGTAGGTTCAGCTGCAGCC-3′ ; 5′- GTCCCGTGGTTCTCAGTGGTA-3′ ; CnAβ1: 5′- GATGTAGGTTCAGCTGCAGCC-3′ , 5′- CCAGTCGATCTGAGGCACAGC-3′ ; MBNL1: 5′- TAGTGTCACACCAATTCGGGACACAAA-3′ , 5′- CCCTTGATGTAATCCATGCAGACAGTGA-3′ ; Gapdh: 5′- GACATGCCGCCTGGAGAAAC-3′ , 5′- AGCCCAGGATGCCCTTTAGT -3′ (rat); Gapdh: 5′- ATGTGCCGGACCTTGGAAG-3′ , 5′- CCTCGGGTTAGCTGAGAGATCA-3′ (mouse).

### RIP, RNAseq and bioinformatics

To determine the mRNAs that directly bind to MBNL1, a Flag-epitope-tagged MBNL1 was adenovirally overexpressed in rat cardiac fibroblasts. Fibroblast lysates were collected 36 h later and then incubated with Flag-M2 affinity resin (Sigma) overnight at 4 °C. The resin was washed and RNA extracted using a Magna RIP kit (Millipore) and phenol-chloroform precipitation. Eluted RNAs were detected by RNAseq analysis using paired-end 100 nucleotide (nt) sequencing and 40 million reads for input-RNAs and RIP-RNAs. FASTQ sequence files were aligned to the rat genome version 5 using TopHat2 with *de novo* junction discovery. Alternative splicing, alternative polyadenylation and differential gene expression analyses along with all visualized pathways, heat maps and Venn diagrams were obtained using AltAnalyze version 2.0.9 (ref. 45), and the Ensembl 77/UCSC mRNA rn5 database or mouse genome version mm9 depending on the experiment, filtering on genes with an FPKM>1. Alternative splicing events were identified using a reciprocal isoform percent spliced difference of at least 25% and putative alternative polyadenylation using a splicing-index difference >2 or ASPIRE *δI* value >0.2, occurring in the last exon of the gene. For all RNAseq experiments, pathways and ontology enrichment analyses were performed with ToppGene^46^ and GO-Elite^47^. All heat maps from AltAnalyze were created using the HOPACH package via a connection to R^48^. Candidate mRNAs were also validated using real-time PCR with 400 ng of first-strand cDNA.

### CRISPR–Cas9 gene editing in *Srf*

CRISPR-Cas9 technology was used to mutate predicted MBNL-binding sites in the *Srf* gene of NIH 3T3 fibroblasts (American Type Culture Collection). Stable cell lines for the mutant *Srf* (*Srf*^*MBNL*^) and a GFP-Cas9n control were created using puromycin selection (1.0 μg ml^−1^). MBNL1-binding sites in SRF's 3′ UTR were predicted using RBPmap software that further ranks the motif based on local clustering of sites and its conservation^35^. The SRF motif used for this study was a neighbouring pair of MBNL1-binding sites shown in green text as follows: 5′- UGCUUACCAACGUG*UGC*UGUG -3′. We created a CRISPR-mediated deletion, which excised both sites using the following guide and protospacer-associated motif (PAM) sequences: 5′- CAACGTGTGCTGTGTGATTG-3′ and TGG. A reverse sequence left shifted by nine base pairs was used to make the other deletion on the sense strand. The single guide RNA (sgRNA) was delivered using the pX462 expression plasmid that contains both the D10A mutant Cas9 nickase and a puromyocin selection cassette. For clarity, the CRISPR-targeted *Srf* sequence is illustrated below with the location of the two sgRNAs (blue brackets), the PAM (red text), and the mutated MBNL1-binding sites (green text).


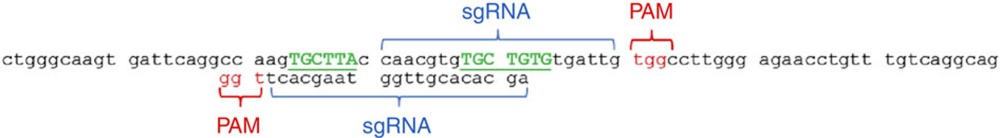


The deletion was confirmed by sequencing across the deletion using genomic DNA obtained from the CRISPR cell line and the new sequence was as follows:


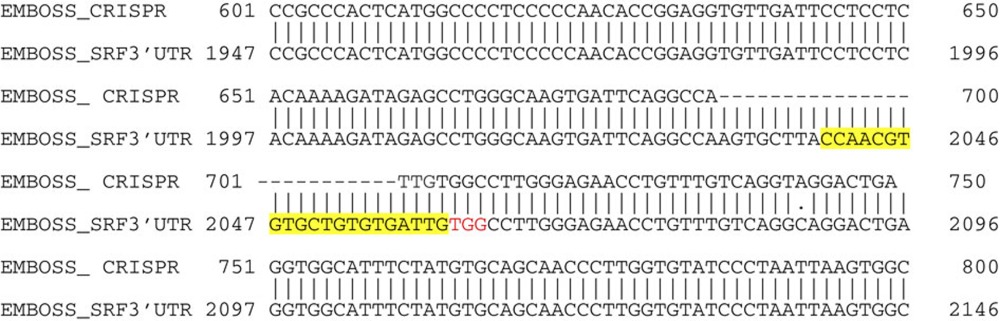


Analysis of the excised region by miRanda software indicated there was no disruption of known microRNA target sites. For orientation, the three bases of the PAM sequence is shown in red again, and the guide sequence is shown in yellow.

### Act.-D transcriptional pulse-chase experiment

Neonatal cardiac fibroblasts or *Srf*^*MBNL*^ CRISPR-mutated fibroblasts were infected or transduced with MBNL1 for 36 h at which time transcription was inhibited with 2 μg ml^−1^ Act.D (Sigma), and RNA was isolated 0, 2, 4 and 8 h later and analysed by real-time PCR.

### Statistics

To determine statistical significance, unpaired *t*-tests were used to compare between two groups, and a one-way analysis of variance was used for multivariate analysis with subsequent pairwise comparisons made using Newman–Keuls tests (Prism software). The mean±the standard error of the mean (s.e.m.) is reported in all figures unless otherwise noted, statistical significance was set a *P*<0.05 and tests for normality were not performed. *In vitro* experiments were repeated three times and animals from multiple litters were used for *in vivo* studies to ensure repeatability. Sample size for animal experiments was determined based on previously reported standards in the field for achieving statistical power, no samples were excluded from the analysis and experiments were not randomized. Data were collected and analysed blinded for both *in vitro* and *in vivo* studies.

## Additional information

**Accession Codes:** RNA-Seq data have been deposited in GEO (Gene Expression Ominbus) of NCBI under accession code GSE74185.

**How to cite this article:** Davis, J. *et al*. MBNL1-mediated regulation of differentiation RNAs promotes myofibroblast transformation and the fibrotic response. *Nat. Commun.* 6:10084 doi: 10.1038/ncomms10084 (2015).

## Supplementary Material

Supplementary Information Supplementary Figures 1-7 and Supplementary Tables 1-2

Supplementary Data 1 A list of MBNL1-bound RNAs (MBNL1 RIP-Seq) as categorized by whether they were alternative spliced, alternatively polyadenylated, or bound with undetermined regulation. Also listed here are RNAs that were alternatively spliced or polyadenylated secondarily in the AdMBNL1 input but not targeted by MBNL1. Transcripts validated by real-time PCR are represented by blue text. Refers to Figure 4.
