# Supplementary material for: MBNL1-mediated regulation of differentiation RNAs promotes myofibroblast transformation and the fibrotic response
Source: Nat Commun. 2015 Dec 16;6:10084. doi: 10.1038/ncomms10084 (PMC4703843; doi:10.1038/ncomms10084)
Supplement: Supplementary Information — Supplementary Figures 1-7 and Supplementary Tables 1-2 [file ncomms10084-s1.pdf]

Supplementary Figure 1. Full length images of the agarose gels and Western blots presented in the manuscript.

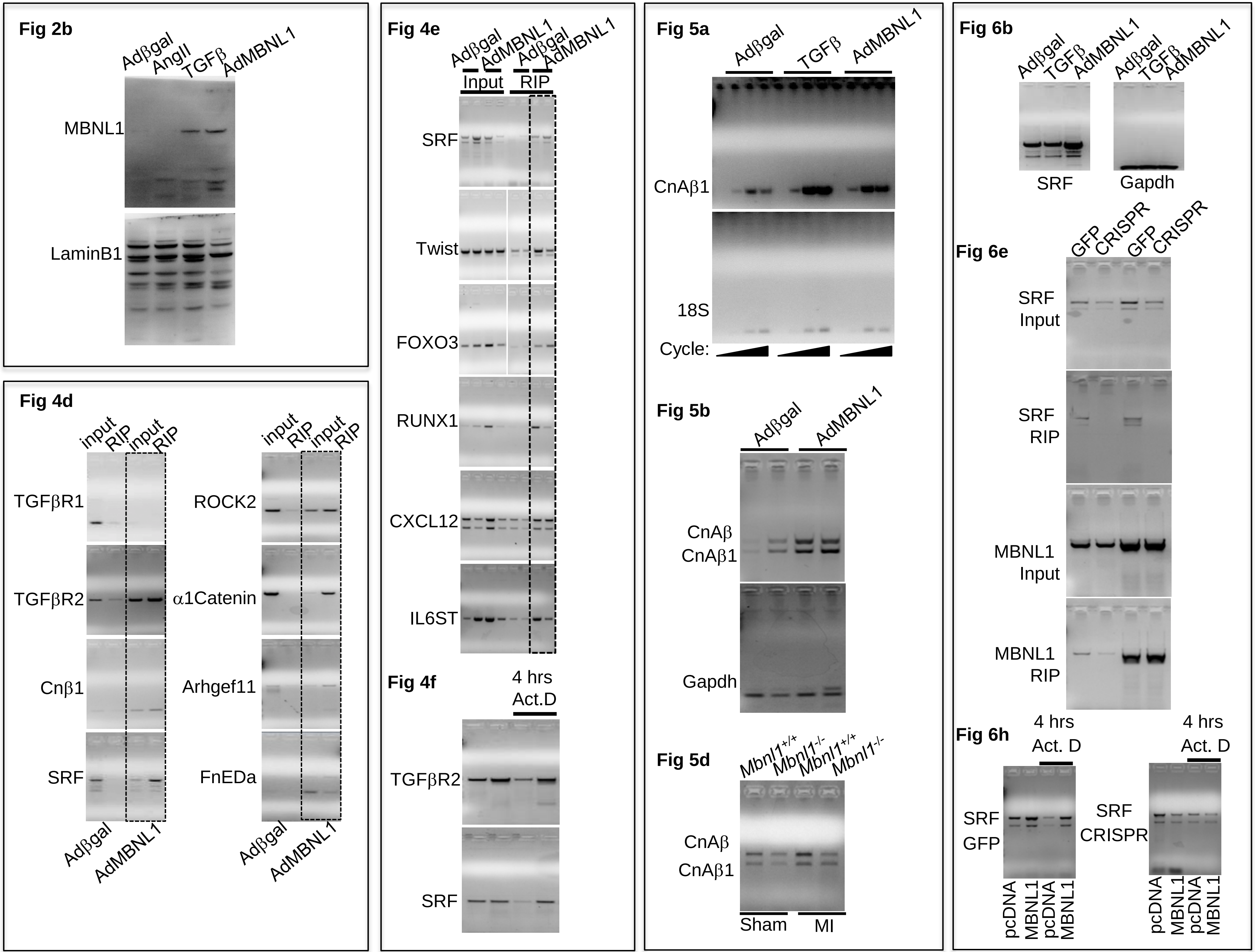

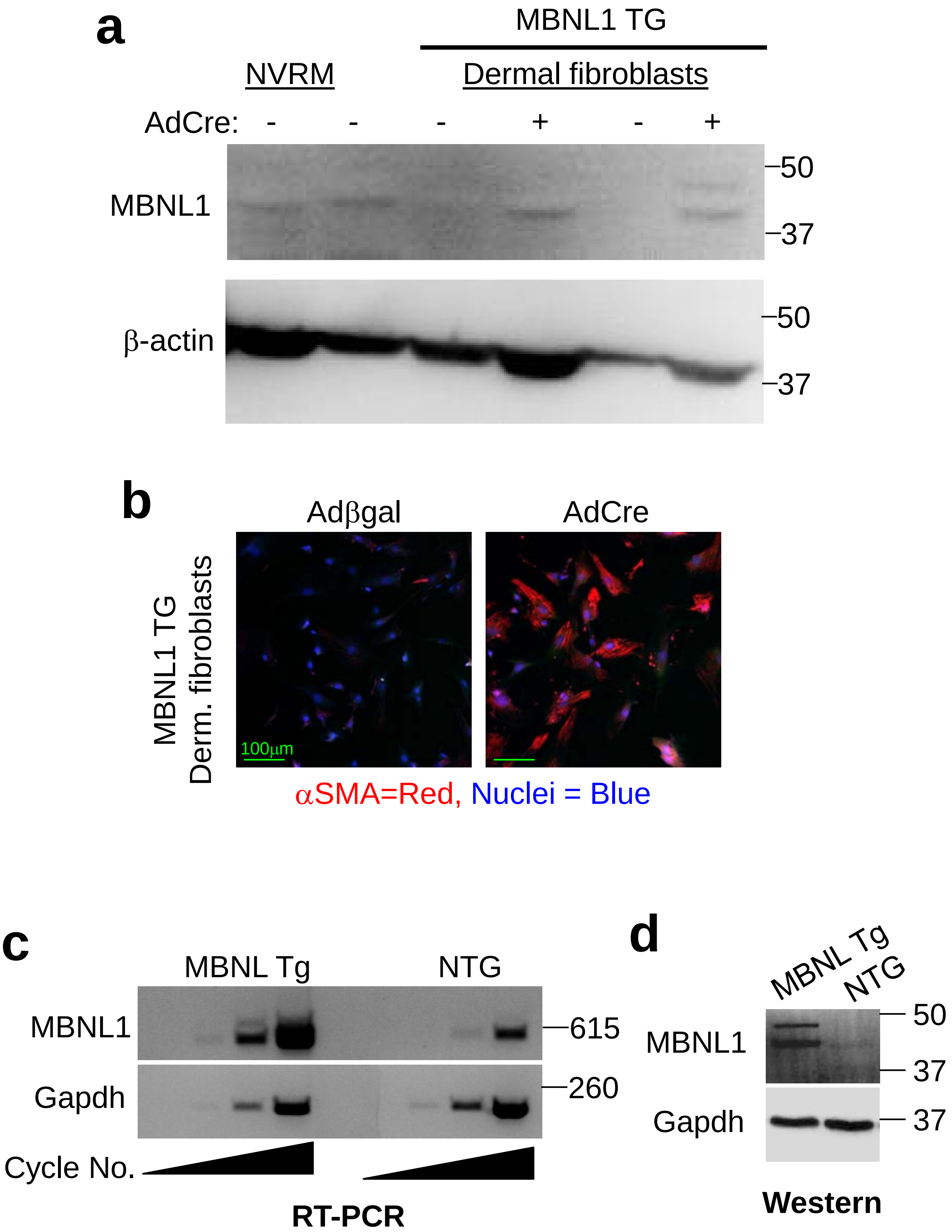

Supplementary Figure 2. Analysis of MBNL1 expression in fibroblasts derived from MBNL1 transgenic mice. (**a**) Western blot analysis of MBNL1 expression in primary dermal fibroblasts isolated from LoxP-dependent MBNL1 TG mice adenovirally infected with either AdCre or Adβgal (-). Neonatal rat cardiac myocytes (NVRM) were used as a positive control for MBNL1 expression. β-actin was used as a loading control. (**b**) Immunofluorescent staining of αSMA-positive (red) stress fibers in primary dermal fibroblasts isolated from LoxP-dependent MBNL1 TG mice adenovirally infected with either AdCre or Adβgal. Scale bar = 30 μm. (**c**) RT-PCR analysis of MBNL1 mRNA in fibroblasts isolated from the hearts of MBNL1 TG-*Tcf21*^{MCM} and NTG-*Tcf21*^{MCM} mice. Gapdh was used as a control. (**d**) Western blot analysis of MBNL1 expression in fibroblasts isolated from the hearts of MBNL1 TG-*Tcf21*^{MCM} and NTG-*Tcf21*^{MCM} mice. Gapdh was used as a loading control. Molecular weight markings are shown on the right of the blots in panels a and d, while DNA size in base pairs is marked on the right of panels in c. Refers to Figure 3

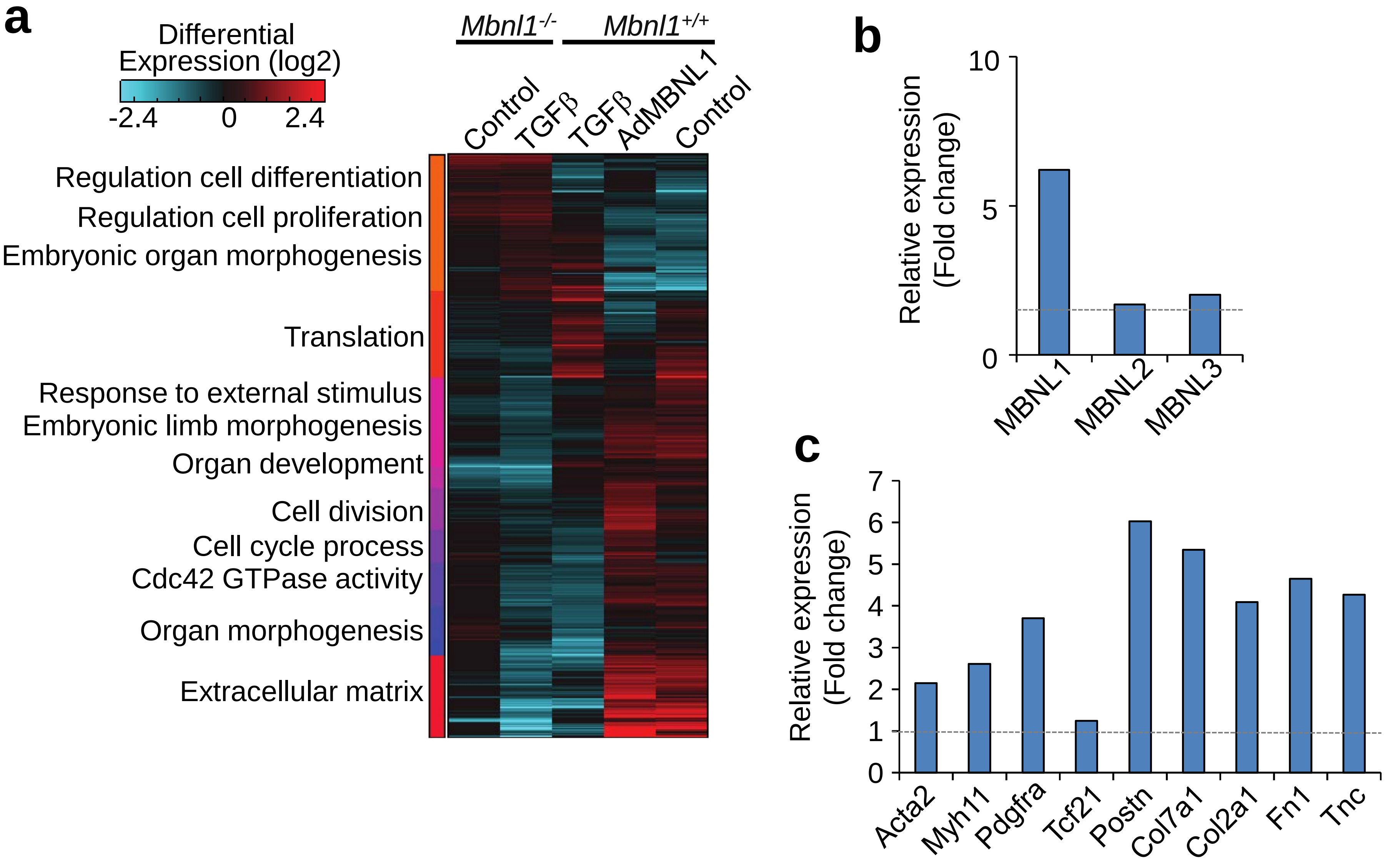

Supplementary Figure 3. MBNL1 transduced MEFs have the genetic profile of a fully matured myofibroblast. (**a**) Heat map depicting differential gene expression clusters generated from RNAseq in *Mbnl1*$^{-/-}$ and *Mbnl1*$^{+/+}$ MEFs with and without TGFβ or AdMBNL1 infection. The list on the left corresponds to changed gene categories in the heat map. (**b**) RNA expression levels of the MBNL gene family (MBNL1-3) in MEFs infected with AdMBNL1. (**c**) Key myofibroblast genes in MEFs infected with AdMBNL1, expressed relative to control, as determined by RNAseq analysis. Refers to Figure 4.

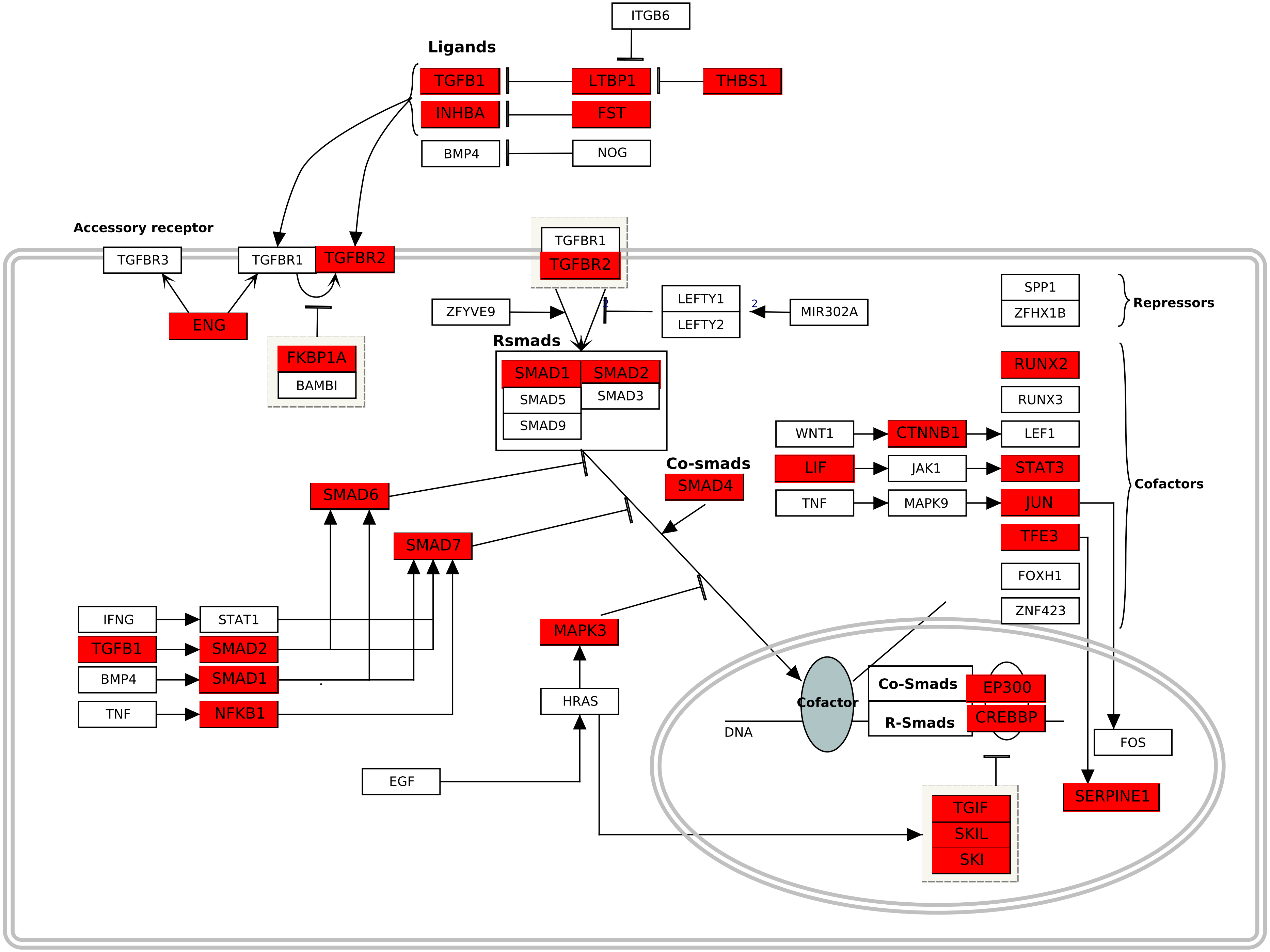

Supplementary Figure 4. Wikipathway for TGFβ signaling (WP560). Transcripts that immunoprecipitated with MBNL1 are colored in red. Refers to Figure 4

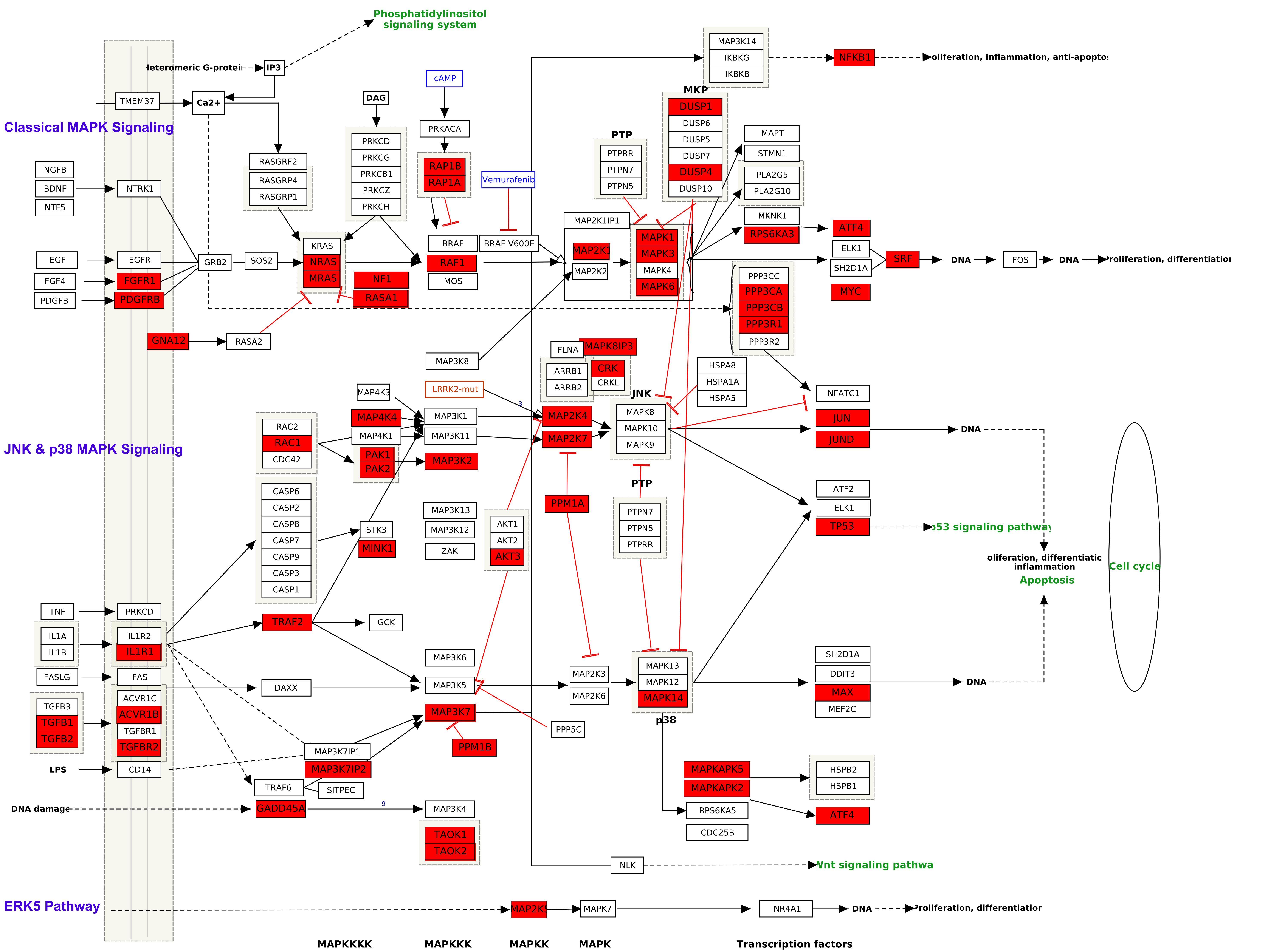

Supplementary Figure 5. Wikipathway for MAPK signaling (WP382). Transcripts that immunoprecipitated with MBNL1 are colored in red. Refers to Figure 4

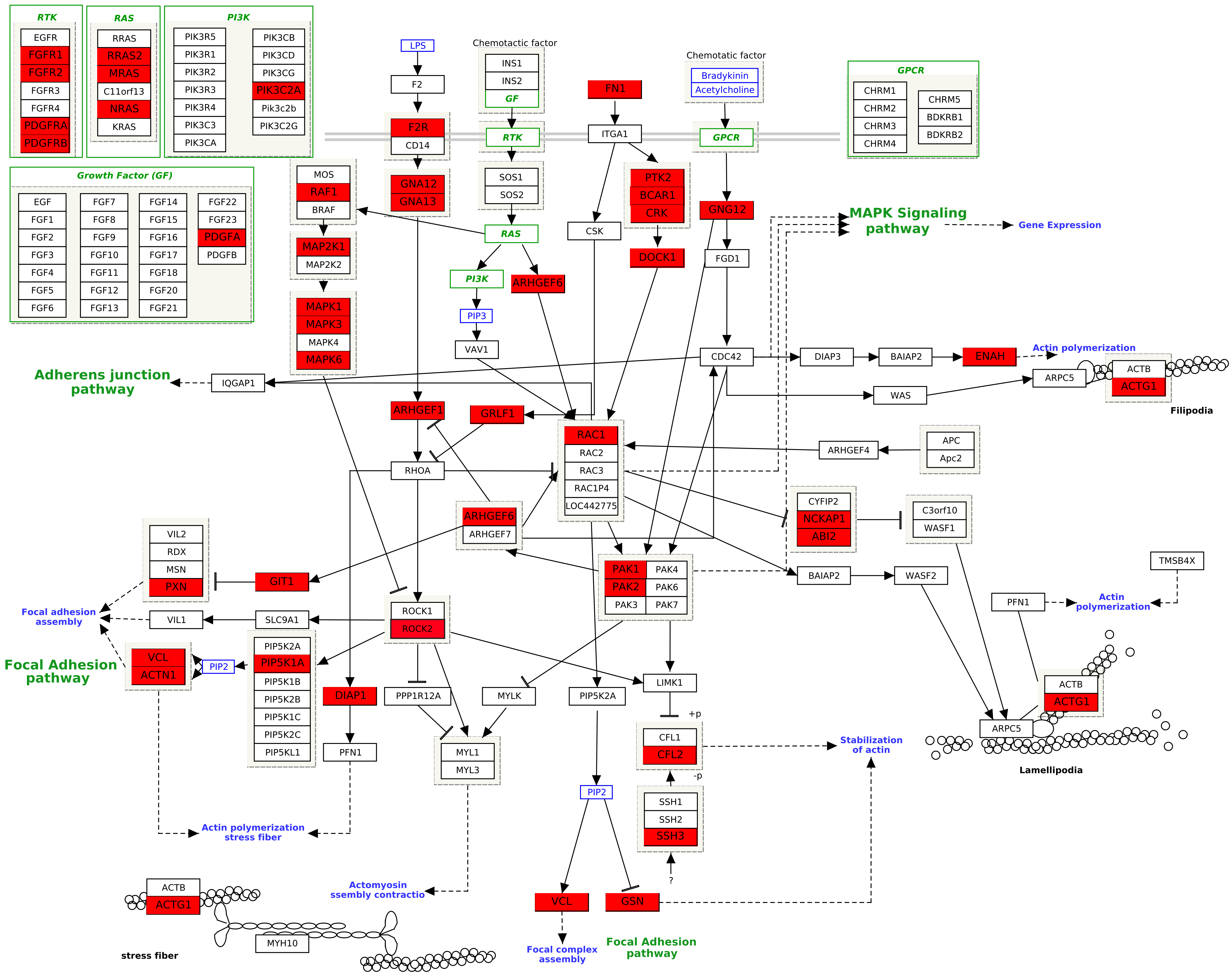

Supplementary FIgure 6 Wikipathway for the regulation of the actin cytoskeleton (WP51). Transcripts that immunoprecipitated with MBNL1 are colored in red. Refers to Figure 4

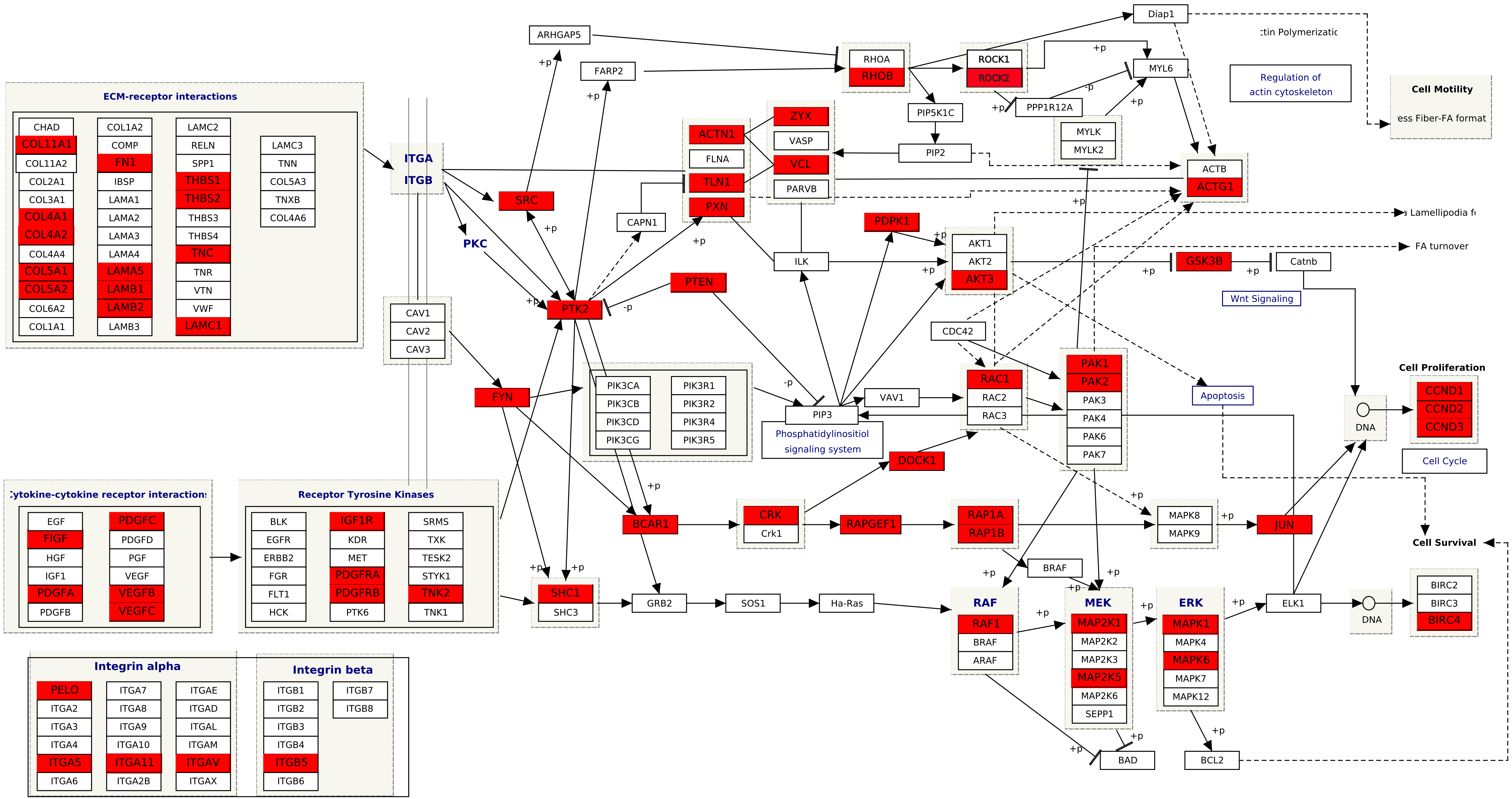

Supplementary FIgure 7 Wikipathway for focal adhesion signaling (WP360). Transcripts that immunoprecipitated with MBNL1 are colored in red. Refers to Figure 4

Supplementary Table 1. MBNL1 RIP targets with identified or predicted conserved binding sites, Related to Figure 4.

A list of MBNL1-bound RNAs (MBNL1 RIP-Seq) categorized by predicted 3'UTR conserved binding sites, CLIP-seq identified 3'UTR binding sites, CLIP-seq identified intronic binding sites, and CLIP-seq identified exonic binding sites. Blue text indicates targets confirmed in Figure 4

RIP Targets with Conserved 3' UTR Binding Sites (Human Sequence)	RIP Targets with Identified 3'UTR Binding Sites (CLIP-Seq)	RIP Targets with Identified Intronic Binding Sites (CLIP-Seq)	RIP Targets with Identified Extronic Binding Sites (CLIP-Seq)
ABHD2	Abi1	Ankrd13a	Acap2
ACVR1	Actc1	Atp2a2	Akt3
ADAMTS1	Actr2	Capza1	Atf6
ADM	Adam10	Dst	Cxadr
AGO2	Aes	Dynll2	Dst
AGTR1	Aff4	Eif5	Enah
ALDH3A2	Akap2	Evl	Lamp2
ALKBH5	Alkbh5	Ext1	Lats2
ANG	Ammecr1l	Fam168a	Luc7l2
ANTXR2	Aplp2	Galnt2	Msi2
AP3S2	Arhgef11	Grb14	Nfia
ARL6IP5	Arih1	Mef2a	Nptn
ATP6AP2	Atp1b1	Pigf	Pdlim5
AXL	Atp1b3	Plekhb2	Plcg1
B4GALT5	Atp2a2	Rmnd5a	Ppp1cb
BCKDHA	Azin1	Sestd1	Rab24
BGN	Bcl7b	Slc39a14	Rcn2
BMP6	Bmpr1a	Snx12	Sdcbp
BMPR2	Cacna2d1	Spata5	Snx12
BNIP3	Camk2d	Tial1	Tnrc18
BUB3	Cbfb	Trak1	Trak2
C1R	Cbx6	Zmiz1	Usp9x
C1S	Cds2		Wapal
C2	Celf1		Yap1
C3	Celf2		Sod2
CADM1	Cggbp1		
CARHSP1	Cited2		

CBFB	Clic4
CCL2	Cmpk1
CCL7	Col4a2
CD151	Crip2
CD200	Csnk1a1
CD55	Ctnnb1
CD59	Dcaf8
CDH2	Degs1
CDKN1A	Dr1
CEBPB	Dync1li2
CEBPD	Dynll2
CELF1	Edem1
CHI3L1	Ehd4
CHPT1	Eif2ak1
CHST11	Eif4e
CITED2	Epc2
CLU	Epdr1
CNPPD1	Fhl2
COL12A1	Fstl1
COL16A1	Gnb1
COL4A1	Golph3
COL4A2	Grk5
COL8A1	Hif1a
COMMD3	Hipk1
CPE	Hipk3
CRIM1	Hnrnpul2
CRLF1	Il6st
CSF1	Inpp5a
CST3	Ivns1abp
CTSH	Klhdc10
CXCL12	Laptm4a
CXCL16	Lman2
CXCL6	Lrrc58
CYR61	Lsm14a
DCN	Mapk1
DDOST	Mapk14
DES	Mat2b
DPAGT1	Max
DPY19L1	Mbnl1
DUSP1	Mbnl2
EDN1	Msi2

EGLN1	Mtpn
EMILIN1	Myadm
ENG	Nampt
ENO2	Ndel1
ENPP2	Nedd4
EPHB4	Nf1
ERAP1	Nfat5
ERO1L	Nploc4
ESYT2	Nudt3
F2R	Ociad1
FAM129B	Otud4
FAM171A1	Oxct1
FBLN1	Pafah1b2
FBXL5	Paip2b
FIGF	Pcmt1
FLRT2	Pdrg1
FMOD	Pls3
FN1	Ppp2r5c
FST	Ppp3cb
FURIN	Ppp3r1
FZD1	Prkacb
GAA	Prkar1a
GABARAPL1	Psap
GABARAPL2	Rab1
GADD45G	Rab10
GALK2	Rab11b
GALNT2	Rab18
GDA	Rab2a
GFPT2	Rap1b
GJA5	Rbfox2
GLB1L	Rbm39
GLIPR1	Rbpms
GLUL	Rftn1
GNS	Rhoq
GPM6B	Rmnd5a
GPR125	Rtn4
GPR176	Serinc3
GREM1	Setd7
GSDMD	Ski
GSTM5	Snx18
GYS1	Snx3

H3F3B	Socs5
H3F3C	Spcs3
HIGD1A	Sqstm1
HM13	Stk24
HMOX1	Strn3
HP	Tbl1xr1
HPRT1	Tead1
HPX	Tial1
HS6ST1	Tmem106b
HSPB8	Tmtc1
ICAM1	Tmx4
IGF1R	Tomm70a
IGFBP3	Tsc22d1
IGFBP7	Tsn
IL1R1	Ttc39b
IL1RL1	Tuba4a
IL4R	Ubqln2
IL6ST	Usp14
IMPAD1	Vamp3
IRF2BP2	Vcl
ISG15	Vezf1
ITGA5	Wbp2
ITM2C	Wnk1
JAM3	Ywhag
JUNB	Zbtb44
KHDRBS1	Zfp106
KLHL24	
LAGE3	
LAMA5	
LAMB1	
LAMP2	
LAPTM4A	
LDHA	
LGALS3BP	
LGALS9	
LMO4	
LOX	
LRP1	
LRP10	
LTBP1	
LTBP2	

LTBP3
LY6E
LYPLA2
MAN2B1
MAOA
MBNL1
MGAT1
MGP
MIF
MMP2
MRC2
MT2A
MX2
NDFIP2
NDUFA2
NFIB
NFKBIA
NOV
NT5E
OLR1
ORMDL3
OSMR
OST4
P4HA2
PALLD
PAM16
PARM1
PBX1
PDGFRA
PDRG1
PELI1
PFKL
PGRMC1
PGRMC2
PLA1A
PLBD2
PLEKHA3
PLOD2
PML
PNRC1
PPAP2A

PRELP
PRNP
PROS1
PRRX1
PSEN2
PSMF1
PTBP3
PTGIS
PTK7
PTX3
RAB32
RANBP9
RASL11B
RBP1
RBPMS
RDM1
RETSAT
RGS16
RGS2
RNASE4
RPP14
SDC4
SELT
SEMA3C
SERINC1
SERPINB9
SERPINE1
SERPING1
SHISA5
SLC2A1
SLC2A3
SLC35B1
SLC39A14
SLC7A2
SLIT3
SMAD7
SMIM14
SMIM7
SMOC1
SMPD1
SOD2

SOD3
SSR3
STAT2
SULF1
SUN2
TAGLN2
TEAD1
TF
TFPI2
TGM2
THBS2
TIMP1
TMEM167B
TMEM176A
TMEM179B
TMEM87A
TNC
TNPO2
TOMM6
TPI1
TPP1
TSPAN31
TSPAN6
TSPO
UAP1
UBALD2
UBC
UBR4
UGCG
UGDH
VCAN
VEGFC
VGLL3
VMP1
WDFY3
WFDC1
WIPI2
YPEL3
ZFAND5
ZFP36L1
ZNRF2

Supplementary Table 2. Fold enrichment of MBNL1 bound messages, refers to Figure 4

Transcript	Input (%)	Fold Enrichment ($2^{-\Delta\Delta Ct}$)
**TGFβR1**	**0.24**	**0.00**
stdev	0.07	0.00
**TGFβR2**	**0.19**	**0.66**
stdev	0.02	0.05
**CnAβ1**	**4.10**	**0.45**
stdev	1.89	0.05
**SRF**	**1.41**	**0.76**
stdev	0.42	0.21
**ROCK2**	**0.49**	**2.26**
stdev	0.15	1.13
**α1CATENIN**	**3.16**	**25.59**
stdev	2.01	12.60
**ARHGEF11**	**1.32**	**47.77**
stdev	0.20	23.70
**TWIST1**	**1.83**	**0.66**
stdev	1.74	0.22
**FOXO3**	**0.21**	**2.54**
stdev	0.05	1.28
**RUNX1**	**0.28**	**5.59**
stdev	0.08	2.60
**CXCL2**	**0.24**	**1.27**
stdev	0.08	0.68
**IL6ST**	**0.29**	**4.71**
stdev	0.04	2.36
**FnEDa**	**0.24**	**0.20**
stdev	0.10	0.10
