# Supplementary material for: MBNL1-mediated regulation of differentiation RNAs promotes myofibroblast transformation and the fibrotic response
Source: Nat Commun. 2015 Dec 16;6:10084. doi: 10.1038/ncomms10084 (PMC4703843; doi:10.1038/ncomms10084)
Supplement: Supplementary Data 1 — A list of MBNL1-bound RNAs (MBNL1 RIP-Seq) as categorized by whether they were alternative spliced, alternatively polyadenylated, or bound with undetermined regulation. Also listed here are RNAs that were alternatively spliced or polyadenylated secondarily in the AdMBNL1 input but not targeted by MBNL1. Transcripts validated by real-time PCR are represented by blue text. Refers to Figure 4. [file ncomms10084-s2.docx]

| **SupplementaryData 1. MBNL1-regulated RNAs Identified by RIP analysis represented by the Venn diagrams, related to Figure 4.   A list of MBNL1-bound RNAs (MBNL1 RIP-Seq) as categorized by whether they were alternative spliced, alternatively polyadenylated, or bound with undetermined regulation. Also listed here are RNAs that were alternatively spliced or polyadenylated secondarily in the AdMBNL1 input but not targeted by MBNL1. Transcripts validated by real-time PCR are represented by blue text. Refers to Figure 4** | | | | | | | |  |
| --- | --- | --- | --- | --- | --- | --- | --- | --- |
| **Bound, Spliced, & Upregulated RNAs** | **Bound & Spliced RNAs** | **Bound & Upregulated RNAs** | **Secondarily Spliced RNAs (unbound)** | **Bound & AltPolyA RNAs** | **Bound, AltPolyA & Upregulated RNAs** | **Secondary AltPolyA RNAs** | **Bound RNAs (undefined Regulation)** |  |
| Cd200 | Abcb1b | Adm | Aak1 | Actr2 | Dcn | Abracl | Aacs |  |
| Enpp2 | Abcf3 | Bnip3 | Abca9 | Akap2 | LOC100909555 | Acp1 | Aars |  |
| LOC100909555 | Abi1 | Bst1 | Abcb1a | Akt3 | Ptx3 | Ankrd1 | Abcc1 |  |
| **Mbnl1** | Abl1 | C1s | Abcd3 | Arpc4 | Rasl11b | Arf1 | Abhd17a |  |
| Pla2g2a | Acap2 | C2 | Abcg3l1 | Atp2a2 | Chic2 | Atf5 | Abhd17b |  |
| Slpi | Acin1 | C3 | Abi3bp | Atp5g3 | Cd55 | Atp5c1 | Abhd17c |  |
| Smoc1 | Acot7 | Ccl2 | Acaca | Atp6v0c | Ubc | Atp5i | Abhd2 |  |
| Sulf1 | Actn1 | Ccl7 | Acad8 | Atp6v0e1 | Jdp2 | Atp6v1e1 | Abhd4 |  |
| Timp1 | Acvr1 | Cd55 | Acly | Atxn2l | Sulf1 | Bcat1 | Abi2 |  |
| Uap1 | Adam10 | Cdkn1a | Acox1 | Bicc1 | Ccl2 | Capg | Abl2 |  |
| Ubc | Adam12 | Cebpb | Actb | Capza1 | Osmr | Cav1 | Abr |  |
|  | Adamts6 | Cebpd | Acvrl1 | Cd44 | Clu | Ccni | Acadl |  |
|  | Adipor1 | Chic2 | Adprm | Cd48 | C1s | Cd74 | Acadvl |  |
|  | Aebp2 | Clu | Agap1 | Cdh2 | **Mbnl1** | Chd4 | Acd |  |
|  | Aff1 | Cxcl1 | Ahctf1 | Cfl2 | Bnip3 | Chmp5 | Acp2 |  |
|  | Agap3 | Cxcl12 | Akap11 | Ckap4 |  | Churc1 | Acsl1 |  |
|  | Agfg1 | Cxcl16 | Aldoa | Cmpk1 |  | Col1a2 | Acsl4 |  |
|  | Agtr1a | Cxcl6 | Alkbh1 | Col5a1 |  | Col3a1 | Acta1 |  |
|  | Akip1 | Dcn | Anapc16 | Cp |  | Cox6c | Actc1 |  |
|  | Ankh | Des | Anapc7 | Csnk1a1 |  | Cr1l | Actg1 |  |
|  | Ankhd1 | F3 | Ankib1 | Cyr61 |  | Creb3 | Actn2 |  |
|  | Ankrd17 | Fbln1 | Ankrd11 | Dag1 |  | Cryab | Acvr1b |  |
|  | Ankrd28 | Fst | Ankrd12 | Dlst |  | Ctbp1 | Adam15 |  |
|  | Ankrd52 | Gadd45g | Anxa5 | Dpysl3 |  | Ctsa | Adam17 |  |
|  | Ano6 | Gda | Ap2m1 | Eif4a2 |  | Ctsb | Adam19 |  |
|  | App | Gfpt2 | Ap3d1 | Epas1 |  | Cttn | Adam33 |  |
|  | Arfip1 | Hmox1 | Arfrp1 | Ext1 |  | Dad1 | Adamts1 |  |
|  | Arfip2 | Hp | Arhgef25 | Fam120a |  | Dstn | Adamts2 |  |
|  | Arhgap10 | Hpx | Arhgef7 | Gls |  | Elovl1 | Adar |  |
|  | Arhgap17 | Hsd11b1 | Ascc2 | Gng12 |  | Eno1 | Adipor2 |  |
|  | Arhgap21 | Igfbp3 | Asic1 | Grk5 |  | Fdft1 | Adk |  |
|  | Arhgap23 | Irf7 | Asmtl | Grsf1 |  | Fdps | Adnp |  |
|  | Arhgef12 | Isg15 | Asnsd1 | Hand2 |  | Fxyd5 | Adsl |  |
|  | Arhgef17 | Jdp2 | Atf2 | Hibadh |  | Gnb2l1 | Aen |  |
|  | Arid1b | Lbp | Atxn7l4 | Hif1a |  | Hnrnpa2b1 | Aes |  |
|  | Arid2 | Lcn2 | Aup1 | Hmga1 |  | Hnrnpa3-ps1 | Aff4 |  |
|  | Arid5b | Lgals9 | Baiap2 | Hnrnph1 |  | Hnrnpm | Aga |  |
|  | Asap1 | LOC100362110 | Bcl2l11 | Ifitm3 |  | Kpna4 | Ago2 |  |
|  | Ash1l | LOC100911625 | Bcor | Ifrd1 |  | Lars | Agpat5 |  |
|  | Asxl1 | Lum | Brd3 | Insig1 |  | Lman1 | Agps |  |
|  | Atg9a | Maoa | Brd4 | Kcmf1 |  | LOC100359593 | Ahcyl1 |  |
|  | Atl3 | Mgat1 | Brms1l | Laptm4a |  | LOC100362640 | Ahr |  |
|  | Atp13a1 | Mgp | C1qtnf1 | Lman2 |  | LOC100909466 | Ahsa1 |  |
|  | Atp8b2 | Mmp2 | C1qtnf7 | LOC100911714 |  | LOC100910474 | AI314180 |  |
|  | Atxn2 | Mt1 | Cald1 | LOC100911865 |  | LOC100911597 | Aip |  |
|  | Atxn2l | Mt2A | Canx | LOC361985 |  | LOC257642 | Ak1 |  |
|  | Axl | Osmr | Cap1 | Maf1 |  | LOC685596 | Ak3 |  |
|  | Bach1 | Pdgfra | Capg | Mtpn |  | Map7d1 | Akap8 |  |
|  | Bag6 | Ptx3 | Cbll1 | Ndfip1 |  | Mcfd2 | Akap8l |  |
|  | Bak1 | Rasl11b | Cbx5 | Nras |  | Metap2 | Akirin2 |  |
|  | Bckdk | Reg3b | Cc2d1b | Nuak1 |  | Mprip | Akr1b8 |  |
|  | Bcl2l1 | Rgs2 | Ccar1 | Odc1 |  | mrpl9 | Akt1s1 |  |
|  | Bcr | Sdc4 | Ccdc107 | Palld |  | Nek7 | Alas1 |  |
|  | Bgn | Serping1 | Ccdc97 | Pcbp2 |  | Nrbp1 | Alcam |  |
|  | Birc6 | Slc2a1 | Ccnt2 | Pdlim5 |  | Ntan1 | Aldh2 |  |
|  | Btbd10 | Sod2 | Cdan1 | Pgrmc2 |  | Nucks1 | Aldh3a2 |  |
|  | Btrc | Sod3 | Cdc14b | Picalm |  | Paip2 | Aldh6a1 |  |
|  | C4a | **Srf** | Cdc37l1 | Ppp1r2 |  | Pfn1 | Alg3 |  |
|  | Cacnb3 | **Tgfbr2** | Cdc40 | Ptbp1 |  | Phgdh | Alkbh3 |  |
|  | Camk2d | Thbs2 | Cdc42ep4 | Ptpn9 |  | Prdx1 | Alkbh5 |  |
|  | Camsap1 | Txnip | Cdc5l | Rab10 |  | Psmc1 | Amd1 |  |
|  | Cbfb | AABR06000596.1 | Cep85 | Rab31 |  | Rad23b | Amfr |  |
|  | Cblb |  | Chd3 | Rbp1 |  | Rarres2 | Ammecr1l |  |
|  | Cbx6 |  | Chd4 | Rcn2 |  | Rbbp4 | Amotl2 |  |
|  | Ccnl1 |  | Ciao1 | Rgs16 |  | Rhoc | Anapc4 |  |
|  | Ccnl2 |  | Clasp1 | Rheb |  | Rock2 | Ang |  |
|  | Ccser2 |  | Clint1 | Rnf10 |  | Rpl7 | Angpt1 |  |
|  | Cdc27 |  | Cluap1 | Rnf13 |  | Rps10 | Ankfy1 |  |
|  | Cdk16 |  | Cnnm3 | Rragc |  | Rps21 | Ankrd13a |  |
|  | Cdkn2aip |  | Cnot1 | RT1-CE2 |  | Rps25 | Ankrd50 |  |
|  | Celf1 |  | Cpsf7 | Sf1 |  | Rps9 | Ankzf1 |  |
|  | CEP170 |  | Crtc1 | Shmt2 |  | Rsl24d1 | Antxr2 |  |
|  | Cfl2 |  | Cryz | Smim7 |  | S100a10 | Anxa11 |  |
|  | Cflar |  | Ctnnbip1 | Sqstm1 |  | Sdha | Ap1ar |  |
|  | Cherp |  | Ctsc | Srsf6 |  | Sfr1 | Ap1b1 |  |
|  | Chst11 |  | Cttn | Strap |  | Sptbn1 | Ap2a1 |  |
|  | Cic |  | Cxcr7 | Sypl1 |  | Ssr1 | Ap3s2 |  |
|  | Ciz1 |  | Cxxc1 | Tex261 |  | St13 | Apex1 |  |
|  | Clcn7 |  | Cyfip1 | Tgfb1i1 |  | Stat1 | Aph1b |  |
|  | Clk2 |  | Cyp4f17 | Thbs1 |  | Tagln | Api5 |  |
|  | Cluh |  | Dab2 | Tmed10 |  | Tax1bp1 | APLP1 |  |
|  | Cmip |  | Dact1 | Tmem30a |  | Tceal8 | Aplp2 |  |
|  | Cnot2 |  | Dbndd2 | Tmem50a |  | Tcf25 | Apoa1bp |  |
|  | Cog3 |  | Dbp | Tnc |  | Thoc7 | Aprt |  |
|  | Col12a1 |  | Dcun1d1 | Ube2h |  | Thy1 | Aqr |  |
|  | Col16a1 |  | Ddx19a | Zfand3 |  | Timm23 | Arap1 |  |
|  | Col18a1 |  | Ddx41 | AABR06056275.2 |  | Tmem176b | Arf3 |  |
|  | Col24a1 |  | Ddx50 | AABR06064176.1 |  | Tpm1 | Arf6 |  |
|  | Col4a3bp |  | Deaf1 | AABR06078903.1 |  | Tubb4b | Arfgap1 |  |
|  | Cops8 |  | Dennd4b |  |  | Tubb6 | Arfgap2 |  |
|  | Cpeb2 |  | Dennd5b |  |  | Txn1 | Arfgef1 |  |
|  | Crtc3 |  | Dfnb31 |  |  | Txn2 | Arfgef2 |  |
|  | Csnk1e |  | Dhrs7 |  |  | Uba3 | Arglu1 |  |
|  | Csnk1g1 |  | Dhx30 |  |  | Uqcrh | Arhgap35 |  |
|  | Csnk1g3 |  | Dido1 |  |  | Vimp | Arhgdia |  |
|  | Ctdsp2 |  | Dip2b |  |  | Vps41 | Arhgef1 |  |
|  | Ctif |  | Dnajc14 |  |  | Wdr1 | **Arhgef11** |  |
|  | Ctnnd1 |  | Dnajc18 |  |  | Zwint | Arhgef2 |  |
|  | Cul7 |  | Dnm2 |  |  | RGD1311703 | Arhgef40 |  |
|  | Cux1 |  | Dscr3 |  |  | AABR06042582.1 | Arhgef6 |  |
|  | Dazap1 |  | Dync1i2 |  |  | AABR06076168.1 | Arid1a |  |
|  | Dbn1 |  | Eea1 |  |  | AABR06078876.3 | Arid4b |  |
|  | Dcaf8 |  | Eef1d |  |  | AABR06078887.1 | Arih1 |  |
|  | Ddhd2 |  | Ehmt1 |  |  |  | Arih2 |  |
|  | Ddr1 |  | Eif5a |  |  |  | Arl2bp |  |
|  | Ddx58 |  | Ell2 |  |  |  | Arl6ip1 |  |
|  | Ddx60 |  | Enoph1 |  |  |  | Arl6ip5 |  |
|  | Dlc1 |  | Epb4.1 |  |  |  | Arl8b |  |
|  | Dlg1 |  | Epb41l2 |  |  |  | Arpc1b |  |
|  | Dlg4 |  | Epb41l3 |  |  |  | Arpc5l |  |
|  | Dnajb12 |  | Epn2 |  |  |  | Arsi |  |
|  | Dnajc25 |  | Eps15 |  |  |  | Asap2 |  |
|  | Dnase1l1 |  | Erbb2ip |  |  |  | Ass1 |  |
|  | Dock7 |  | Ergic2 |  |  |  | Atad1 |  |
|  | Dot1l |  | Etv4 |  |  |  | Atf1 |  |
|  | Dpf2 |  | Eva1b |  |  |  | Atf4 |  |
|  | Dpp8 |  | Evc2 |  |  |  | Atf6 |  |
|  | Dst |  | Evi5 |  |  |  | Atf6b |  |
|  | Dut |  | Exoc5 |  |  |  | Atf7 |  |
|  | Dync1h1 |  | Exoc7 |  |  |  | Atf7ip |  |
|  | Dyrk1a |  | Fam114a2 |  |  |  | Atic |  |
|  | E2f6 |  | Fam13a |  |  |  | Atn1 |  |
|  | Ece1 |  | Fam172a |  |  |  | Atp10a |  |
|  | Ecm1 |  | Fbxl20 |  |  |  | Atp11a |  |
|  | Efr3a |  | Fbxl3 |  |  |  | Atp13a3 |  |
|  | Efs |  | Fbxo31 |  |  |  | Atp1a1 |  |
|  | Eif4g1 |  | Fcgr2a |  |  |  | Atp1b1 |  |
|  | Elp3 |  | Fdps |  |  |  | Atp1b3 |  |
|  | Epc1 |  | Fibp |  |  |  | Atp2b1 |  |
|  | Eps15l1 |  | Fip1l1 |  |  |  | Atp5d |  |
|  | Evl |  | Fkbp15 |  |  |  | Atp5g2 |  |
|  | Fam13b |  | Flcn |  |  |  | Atp6ap1 |  |
|  | Fam193a |  | Flnc |  |  |  | Atp6ap2 |  |
|  | Fam91a1 |  | Flywch1 |  |  |  | Atp6v1a |  |
|  | Farp1 |  | Fmr1 |  |  |  | Atp6v1b2 |  |
|  | Fastk |  | Fnbp1 |  |  |  | Atp6v1f |  |
|  | Fgfr1 |  | Fnip1 |  |  |  | Atpaf2 |  |
|  | Fhl1 |  | Fopnl |  |  |  | Atrn |  |
|  | Figf |  | Fubp3 |  |  |  | Atxn7l3 |  |
|  | Flnb |  | G4 |  |  |  | Atxn7l3b |  |
|  | Flrt2 |  | Gapvd1 |  |  |  | Auts2 |  |
|  | **Fn1** |  | Gas2l1 |  |  |  | Axin1 |  |
|  | Fnbp4 |  | Gbf1 |  |  |  | Azin1 |  |
|  | Fndc3b |  | Gdi1 |  |  |  | B2m |  |
|  | Foxj3 |  | Ggnbp2 |  |  |  | B4galt1 |  |
|  | Foxp1 |  | Git2 |  |  |  | B4galt5 |  |
|  | Frmd6 |  | Gnl2 |  |  |  | B4galt7 |  |
|  | Gcn1l1 |  | Golga2 |  |  |  | Bace2 |  |
|  | Golga4 |  | Golgb1 |  |  |  | Bag3 |  |
|  | Gpr125 |  | Gpaa1 |  |  |  | Bap1 |  |
|  | Grk5 |  | Gpbp1 |  |  |  | Baz1b |  |
|  | Gtf2a1 |  | Gpr153 |  |  |  | Bcar1 |  |
|  | H2afy |  | Gpr56 |  |  |  | Bckdha |  |
|  | Hbp1 |  | Gpt2 |  |  |  | Bcl7b |  |
|  | Hdac7 |  | Gramd1b |  |  |  | Bcl9l |  |
|  | Hdgfrp3 |  | Grik5 |  |  |  | Becn1 |  |
|  | Hectd1 |  | Gripap1 |  |  |  | Bet1 |  |
|  | Heg1 |  | Grpel1 |  |  |  | Bet1l |  |
|  | Hgsnat |  | Gtf2i |  |  |  | Bhlhe40 |  |
|  | Hipk1 |  | Gtf3a |  |  |  | Blmh |  |
|  | Hnrnpc |  | Guk1 |  |  |  | Bmi1 |  |
|  | Hnrnpdl |  | H6pd |  |  |  | Bmp1 |  |
|  | Hnrnpf |  | Hax1 |  |  |  | Bmp6 |  |
|  | Hnrnph3 |  | Hcfc1 |  |  |  | Bmpr1a |  |
|  | HPS |  | Hcfc1r1 |  |  |  | Bmpr2 |  |
|  | Hs2st1 |  | Herc1 |  |  |  | Bnip3l |  |
|  | Hspg2 |  | Hmbox1 |  |  |  | Brap |  |
|  | Htra2 |  | Hmg20b |  |  |  | Brd1 |  |
|  | Huwe1 |  | Hnrnpa1 |  |  |  | Brd2 |  |
|  | Idua |  | Hnrnpa3-ps1 |  |  |  | Bri3 |  |
|  | Ifi44 |  | Hnrnpk |  |  |  | Bri3bp |  |
|  | Ifit3 |  | Hnrnpl |  |  |  | Brox |  |
|  | Igf2r |  | Hp1bp3 |  |  |  | Bsg |  |
|  | Il1r1 |  | Hps4 |  |  |  | Bst2 |  |
|  | Il4r |  | Hsd3b7 |  |  |  | Btbd19 |  |
|  | Ilf3 |  | Hspb2 |  |  |  | Btg1 |  |
|  | Ing4 |  | Iars |  |  |  | BUB3 |  |
|  | Inhba |  | Ibtk |  |  |  | Bzw1 |  |
|  | Ipo9 |  | Ide |  |  |  | C1qbp |  |
|  | Iqsec1 |  | Il11ra1 |  |  |  | C1qtnf6 |  |
|  | Irak1 |  | Immt |  |  |  | C1r |  |
|  | Itpr1 |  | Ino80e |  |  |  | Cab39 |  |
|  | Itsn1 |  | Ints9 |  |  |  | Cables2 |  |
|  | Jak2 |  | Ipo8 |  |  |  | Cacfd1 |  |
|  | Jmjd8 |  | Isy1 |  |  |  | Cacna1c |  |
|  | Kalrn |  | Itgb1bp1 |  |  |  | Cacna2d1 |  |
|  | Kansl1 |  | Itsn2 |  |  |  | Cacul1 |  |
|  | Kansl3 |  | Jmy |  |  |  | Cadm1 |  |
|  | Kdm5b |  | Kars |  |  |  | Cadm4 |  |
|  | Klhl13 |  | Kctd18 |  |  |  | Calcoco1 |  |
|  | Klhl24 |  | Kctd9 |  |  |  | Calm1 |  |
|  | Kmt2d |  | Kdm1a |  |  |  | Camk2n1 |  |
|  | Lama5 |  | Kdm5a |  |  |  | Cand1 |  |
|  | Lamb2 |  | Kdm5c |  |  |  | Caprin1 |  |
|  | Lamtor1 |  | Kif13a |  |  |  | Car3 |  |
|  | Lilrb3l |  | Klc1 |  |  |  | Carhsp1 |  |
|  | LOC100909455 |  | Klc2 |  |  |  | Carm1 |  |
|  | LOC100910121 |  | Krr1 |  |  |  | Ccdc19 |  |
|  | LOC100910212 |  | Ktn1 |  |  |  | Ccdc47 |  |
|  | LOC100910717 |  | Lamtor3 |  |  |  | Ccdc6 |  |
|  | LOC100910833 |  | Larp1b |  |  |  | Ccdc71 |  |
|  | LOC100911602 |  | Lcor |  |  |  | Ccl6 |  |
|  | LOC100911865 |  | Letmd1 |  |  |  | Ccnc |  |
|  | LOC100911881 |  | Lnp |  |  |  | Ccnd1 |  |
|  | LOC102546978 |  | LOC100233176 |  |  |  | Ccnd2 |  |
|  | LOC102547059 |  | LOC100909466 |  |  |  | Ccnd3 |  |
|  | LOC102552895 |  | LOC100909580 |  |  |  | Ccndbp1 |  |
|  | LOC102555086 |  | LOC100910272 |  |  |  | Ccne1 |  |
|  | LOC102556337 |  | LOC100911205 |  |  |  | Ccng1 |  |
|  | LOC498369 |  | LOC100911365 |  |  |  | Ccnt1 |  |
|  | LOC678893 |  | LOC100911440 |  |  |  | Ccny |  |
|  | LOC685067 |  | LOC100911495 |  |  |  | Ccnyl1 |  |
|  | LOC687295 |  | LOC100911674 |  |  |  | Ccs |  |
|  | Loxl2 |  | LOC100911721 |  |  |  | Cd151 |  |
|  | Lphn2 |  | LOC100911825 |  |  |  | Cd164 |  |
|  | Lpp |  | LOC100911841 |  |  |  | Cd2bp2 |  |
|  | Lrp10 |  | LOC100912115 |  |  |  | Cd47 |  |
|  | Lrrc47 |  | LOC100912456 |  |  |  | Cd59 |  |
|  | Macf1 |  | LOC102550124 |  |  |  | Cd81 |  |
|  | Mafg |  | LOC102553866 |  |  |  | Cdc123 |  |
|  | Map4k4 |  | LOC257642 |  |  |  | Cdc16 |  |
|  | Mapk1 |  | LOC681069 |  |  |  | Cdc37 |  |
|  | Mapkap1 |  | LOC682914 |  |  |  | Cdc42bpb |  |
|  | Mast4 |  | LOC686442 |  |  |  | Cdc42ep1 |  |
|  | Mcc |  | LOC690948 |  |  |  | Cdc42ep5 |  |
|  | Mdm4 |  | LOC691920 |  |  |  | Cdc42se1 |  |
|  | Med13l |  | Lonp2 |  |  |  | Cdc73 |  |
|  | Mef2a |  | Lrp11 |  |  |  | Cdca4 |  |
|  | Mff |  | Lrrc8a |  |  |  | Cdh13 |  |
|  | Mfhas1 |  | Lrrfip1 |  |  |  | Cdipt |  |
|  | Mgrn1 |  | Lrrfip2 |  |  |  | Cdk13 |  |
|  | Mical2 |  | Lsm14b |  |  |  | Cdk14 |  |
|  | Midn |  | Lsp1 |  |  |  | Cdk2ap1 |  |
|  | Mpnd |  | Mafk |  |  |  | Cdk6 |  |
|  | Mras |  | Man1a1 |  |  |  | Cdk7 |  |
|  | Mrc2 |  | Map4k3 |  |  |  | Cdk9 |  |
|  | Msrb3 |  | Map7d1 |  |  |  | Cdkn1b |  |
|  | Mtfr1l |  | Mbd3 |  |  |  | Cdkn2b |  |
|  | Mtmr2 |  | Mboat7 |  |  |  | Cdr2l |  |
|  | Myo10 |  | Mbtps2 |  |  |  | Cds2 |  |
|  | Myo1c |  | Mcm7 |  |  |  | Cdyl |  |
|  | Myo9b |  | Mcoln1 |  |  |  | Celf2 |  |
|  | Myrf |  | Med20 |  |  |  | Cenpb |  |
|  | Naa25 |  | Mettl17 |  |  |  | Cep170b |  |
|  | Naa50 |  | Mief1 |  |  |  | Cers2 |  |
|  | Nacc1 |  | Mllt6 |  |  |  | Cers5 |  |
|  | Nacc2 |  | Mocs1 |  |  |  | Cers6 |  |
|  | Nbas |  | Mocs2 |  |  |  | Cggbp1 |  |
|  | Ncor2 |  | Mospd1 |  |  |  | Chac1 |  |
|  | Ndrg2 |  | Mpa2l |  |  |  | Chi3l1 |  |
|  | Neo1 |  | Mpc2 |  |  |  | Chka |  |
|  | Nfat5 |  | Mprip |  |  |  | Chmp1a |  |
|  | Nfib |  | mrpl9 |  |  |  | Chp1 |  |
|  | Nfic |  | Mrps18c |  |  |  | Chpf |  |
|  | Nfix |  | Ms4a6bl |  |  |  | Chpt1 |  |
|  | Nfkb1 |  | Msh6 |  |  |  | Chsy1 |  |
|  | Nhsl1 |  | Msl3 |  |  |  | Chtop |  |
|  | Npepl1 |  | Mtmr3 |  |  |  | Cir1 |  |
|  | Npr2 |  | Mtus1 |  |  |  | Cirh1a |  |
|  | Nr1d1 |  | Mxra7 |  |  |  | Cit |  |
|  | Nr1h2 |  | Mybl1 |  |  |  | Cited2 |  |
|  | Nr2c2 |  | Mycbp2 |  |  |  | Clasrp |  |
|  | Nrp2 |  | Myl12a |  |  |  | Clec2dl1 |  |
|  | Odc1 |  | Myo1b |  |  |  | Clec2g |  |
|  | Orc4 |  | Naa15 |  |  |  | Clic4 |  |
|  | Ormdl3 |  | Nab2 |  |  |  | Clk1 |  |
|  | Otud5 |  | Nabp1 |  |  |  | Clk3 |  |
|  | Otud7b |  | Naglu |  |  |  | Clk4 |  |
|  | Pabpc4 |  | Nap1l1 |  |  |  | Clmp |  |
|  | Pacs2 |  | Ndufs1 |  |  |  | Clptm1 |  |
|  | Pan3 |  | Ndufv3 |  |  |  | Clptm1l |  |
|  | Pank2 |  | Necap1 |  |  |  | Clstn1 |  |
|  | Papola |  | Necap2 |  |  |  | Cmtm6 |  |
|  | Pappa |  | Nelfe |  |  |  | Cmtr1 |  |
|  | Patl1 |  | Neurl4 |  |  |  | Cnbd2 |  |
|  | Patz1 |  | Nexn |  |  |  | Cnbp |  |
|  | Pcbp2 |  | Nf2 |  |  |  | Cnep1r1 |  |
|  | Pde4dip |  | Ngfrap1 |  |  |  | Cnih1 |  |
|  | Pdlim5 |  | Nol3 |  |  |  | Cnksr3 |  |
|  | Pdpk1 |  | Nop2 |  |  |  | Cnn2 |  |
|  | Phc3 |  | Nr2c2ap |  |  |  | Cnot4 |  |
|  | Pias2 |  | Nrg1 |  |  |  | Cnot6 |  |
|  | Piezo1 |  | Nsd1 |  |  |  | Cnot6l |  |
|  | Pkd1 |  | Nsun2 |  |  |  | Cnot8 |  |
|  | Pkn1 |  | Ntan1 |  |  |  | Cnppd1 |  |
|  | Pkp1 |  | Nup205 |  |  |  | Cnpy3 |  |
|  | Pla1a |  | Nup54 |  |  |  | Coa5 |  |
|  | Plcb4 |  | Odf2 |  |  |  | Col11a1 |  |
|  | Plcg1 |  | Opa1 |  |  |  | Col4a1 |  |
|  | Pld1 |  | Opn3 |  |  |  | Col4a2 |  |
|  | Plec |  | Osbpl1a |  |  |  | Col4a5 |  |
|  | Plxna1 |  | Oxr1 |  |  |  | Col5a2 |  |
|  | Pml |  | Park7 |  |  |  | Col8a1 |  |
|  | Ppid |  | Paxip1 |  |  |  | Col9a3 |  |
|  | Ppp1r10 |  | Pbrm1 |  |  |  | Colgalt1 |  |
|  | Ppp2r5c |  | Pcnx |  |  |  | Commd3 |  |
|  | **Ppp3cb** |  | Pcnxl3 |  |  |  | Copa |  |
|  | Ppp4r1 |  | Pcsk6 |  |  |  | Cox5a |  |
|  | Prkab1 |  | Pdcd10 |  |  |  | Cox5b |  |
|  | Prpf39 |  | Pdcl |  |  |  | Cox6a2 |  |
|  | Prpsap1 |  | Pde4d |  |  |  | Cpd |  |
|  | Prrc2b |  | Pdhx |  |  |  | Cpe |  |
|  | Prrc2c |  | Pepd |  |  |  | Cpeb4 |  |
|  | Prtfdc1 |  | Pex5 |  |  |  | Cpne1 |  |
|  | Ptbp1 |  | Pfkfb3 |  |  |  | Cpne3 |  |
|  | Ptk2 |  | Phc2 |  |  |  | Crat |  |
|  | Ptpla |  | Phf1 |  |  |  | Creb3l1 |  |
|  | Puf60 |  | Phf14 |  |  |  | Creb3l2 |  |
|  | Pum1 |  | Phf21a |  |  |  | Crebbp |  |
|  | Pum2 |  | Phf6 |  |  |  | Crebrf |  |
|  | QK |  | Phldb2 |  |  |  | Crebzf |  |
|  | R3hdm1 |  | Pigq |  |  |  | Crim1 |  |
|  | R3hdm2 |  | Pik3r2 |  |  |  | Crip2 |  |
|  | Rab31 |  | Pikfyve |  |  |  | Crk |  |
|  | Ranbp9 |  | Pilra |  |  |  | Crlf1 |  |
|  | Rangap1 |  | Plin3 |  |  |  | Crtap |  |
|  | Rapgef1 |  | Plscr1 |  |  |  | Crtc2 |  |
|  | Raph1 |  | Ppan |  |  |  | Cs |  |
|  | Rassf8 |  | Ppfia1 |  |  |  | Csf1 |  |
|  | Rbm33 |  | Ppfibp1 |  |  |  | Csnk1d |  |
|  | Rbm5 |  | Ppil4 |  |  |  | Csnk1g2 |  |
|  | Rbms3 |  | Ppip5k1 |  |  |  | Csnk2a1 |  |
|  | Rbpms |  | Ppp1cc |  |  |  | Csnk2a2 |  |
|  | Rc3h1 |  | Ppp1r15b |  |  |  | Csrp2 |  |
|  | Rcan1 |  | Ppp2r5d |  |  |  | Cst3 |  |
|  | Rere |  | Ppp3cc |  |  |  | Ctdnep1 |  |
|  | Rev1 |  | Ppp5c |  |  |  | Ctdp1 |  |
|  | RGD1309621 |  | Prickle2 |  |  |  | Ctdsp1 |  |
|  | RGD1565536 |  | Prkci |  |  |  | Ctdspl |  |
|  | RGD1565775 |  | Prpf40a |  |  |  | Ctgf |  |
|  | Rgs3 |  | Psd3 |  |  |  | **Ctnna1** |  |
|  | Rin2 |  | Psmb9 |  |  |  | Ctnnb1 |  |
|  | Ripk2 |  | PTPRK |  |  |  | Ctsf |  |
|  | Rnf149 |  | Ptrh1 |  |  |  | Ctsh |  |
|  | Rnf216 |  | Pvrl2 |  |  |  | Cul3 |  |
|  | Rnf4 |  | Rab30 |  |  |  | Cul4a |  |
|  | Rnf40 |  | Rabep1 |  |  |  | Cul5 |  |
|  | Rnf41 |  | Rai14 |  |  |  | Cwc27 |  |
|  | Rnf44 |  | Ralgapb |  |  |  | Cxadr |  |
|  | Rnpepl1 |  | Rassf1 |  |  |  | Cyp1b1 |  |
|  | Rprd2 |  | Rbbp6 |  |  |  | Cyp20a1 |  |
|  | Rps6ka3 |  | Rbm10 |  |  |  | Cystm1 |  |
|  | Rrbp1 |  | Rbm34 |  |  |  | Dab2ip |  |
|  | Rsrp1 |  | RGD1304567 |  |  |  | Dap |  |
|  | RT1-A2 |  | RGD1305464 |  |  |  | Dap3 |  |
|  | RT1-CE2 |  | RGD1306487 |  |  |  | Dapk3 |  |
|  | RT1-CE3 |  | RGD1307500 |  |  |  | Dcakd |  |
|  | RT1-S3 |  | RGD1307830 |  |  |  | Dcbld2 |  |
|  | RT1-T24-4 |  | RGD1308134 |  |  |  | Dcp2 |  |
|  | Rtn4 |  | RGD1310712 |  |  |  | Dctn4 |  |
|  | Rwdd4 |  | RGD1311747 |  |  |  | Ddah1 |  |
|  | Samd4a |  | RGD1311783 |  |  |  | Ddit4 |  |
|  | Scamp4 |  | RGD1359310 |  |  |  | Ddost |  |
|  | Schip1 |  | RGD1559904 |  |  |  | Ddr2 |  |
|  | Sec16a |  | Rhog |  |  |  | Ddx17 |  |
|  | Sec24c |  | Rictor |  |  |  | Ddx39a |  |
|  | Sec31a |  | Rit1 |  |  |  | Ddx39b |  |
|  | Sesn2 |  | Rlf |  |  |  | Ddx3x |  |
|  | Sf1 |  | Rnf126 |  |  |  | Ddx49 |  |
|  | Sgpl1 |  | Rnf130 |  |  |  | Ddx6 |  |
|  | Siva1 |  | Rnf138 |  |  |  | Dedd |  |
|  | Skil |  | Rpl26 |  |  |  | Degs1 |  |
|  | Slain2 |  | Rps25 |  |  |  | Dennd5a |  |
|  | Slc25a22 |  | Rrp36 |  |  |  | Deptor |  |
|  | Slc25a25 |  | Rsrc2 |  |  |  | Derl1 |  |
|  | Slc38a1 |  | RT1-CE16 |  |  |  | Desi1 |  |
|  | Slc38a10 |  | Rundc1 |  |  |  | Dgat1 |  |
|  | Slc39a14 |  | Rxrb |  |  |  | Dhcr24 |  |
|  | Slfn3 |  | Sae1 |  |  |  | Dhx9 |  |
|  | Smek1 |  | Samd8 |  |  |  | Diablo |  |
|  | Smg1 |  | Sap130 |  |  |  | Diaph1 |  |
|  | Smg7 |  | Sash1 |  |  |  | Dis3l2 |  |
|  | Smurf2 |  | Sccpdh |  |  |  | Dlgap4 |  |
|  | Snrnp70 |  | Scyl2 |  |  |  | Dnaja1 |  |
|  | Snx13 |  | Sdhaf2 |  |  |  | Dnaja2 |  |
|  | Son |  | Sec23a |  |  |  | Dnaja3 |  |
|  | Sorbs1 |  | Selplg |  |  |  | Dnajb2 |  |
|  | Specc1 |  | Sema3f |  |  |  | Dnajb4 |  |
|  | Sppl2a |  | Sephs1 |  |  |  | Dnajb6 |  |
|  | Spry1 |  | Setd5 |  |  |  | Dnajc13 |  |
|  | Sptan1 |  | Sf3a2 |  |  |  | Dnajc5 |  |
|  | Srcap |  | Sh2b3 |  |  |  | Dock1 |  |
|  | Srek1 |  | Sirt1 |  |  |  | Dpagt1 |  |
|  | Srsf11 |  | Sirt2 |  |  |  | Dpp3 |  |
|  | Srsf4 |  | Slc11a2 |  |  |  | Dpp7 |  |
|  | Srsf5 |  | Slc27a4 |  |  |  | Dpp9 |  |
|  | Srsf7 |  | Slc35e3 |  |  |  | Dpy19l1 |  |
|  | St3gal4 |  | Slc7a6os |  |  |  | Dr1 |  |
|  | St6galnac4 |  | Smad3 |  |  |  | Drg1 |  |
|  | Stag2 |  | Smad5 |  |  |  | Drg2 |  |
|  | Stk24 |  | Smap1 |  |  |  | Dtx3 |  |
|  | Sun2 |  | Smarca2 |  |  |  | Dusp1 |  |
|  | Syncrip |  | Smarcb1 |  |  |  | Dusp4 |  |
|  | Synpo |  | Smtnl1 |  |  |  | Dvl1 |  |
|  | Taok2 |  | Snapc2 |  |  |  | Dvl3 |  |
|  | Tead1 |  | Snrpb |  |  |  | Dync1li1 |  |
|  | Tfpi |  | Soat1 |  |  |  | Dync1li2 |  |
|  | Tfpi2 |  | Soga1 |  |  |  | Dynll2 |  |
|  | Thbs1 |  | Sorbs2 |  |  |  | E2f4 |  |
|  | Tial1 |  | Sp1 |  |  |  | Echs1 |  |
|  | Tjp1 |  | Sp100 |  |  |  | Edem1 |  |
|  | Tle3 |  | Sp140 |  |  |  | Edem3 |  |
|  | Tlk2 |  | Spen |  |  |  | Edn1 |  |
|  | Tmem39a |  | Sphk1 |  |  |  | Efemp2 |  |
|  | Tmem87a |  | Spns1 |  |  |  | Efhd2 |  |
|  | Tnc |  | Sptbn1 |  |  |  | Efna5 |  |
|  | Tnip1 |  | Sqle |  |  |  | Efnb2 |  |
|  | Tnks2 |  | Srpk2 |  |  |  | Egln1 |  |
|  | Tnpo2 |  | Srrm1 |  |  |  | Egr1 |  |
|  | Tnpo3 |  | Srrt |  |  |  | Ehbp1l1 |  |
|  | Tnrc18 |  | St3gal5 |  |  |  | Ehd2 |  |
|  | Tnrc6a |  | Stat1 |  |  |  | Ehd4 |  |
|  | Tns1 |  | Strn4 |  |  |  | Ehmt2 |  |
|  | Tns3 |  | Stxbp2 |  |  |  | Eif1 |  |
|  | Tra2a |  | Sub1 |  |  |  | Eif1a |  |
|  | Tra2b |  | Sugp1 |  |  |  | Eif1b |  |
|  | Traf7 |  | Sun1 |  |  |  | Eif2ak1 |  |
|  | Trib3 |  | Supt6h |  |  |  | Eif2ak2 |  |
|  | Trim3 |  | Suv420h1 |  |  |  | Eif4e |  |
|  | Trim33 |  | Syngap1 |  |  |  | Eif4ebp2 |  |
|  | Trip6 |  | Szrd1 |  |  |  | Eif4enif1 |  |
|  | Trmt1 |  | Tacc1 |  |  |  | Eif4g3 |  |
|  | Ttc13 |  | Taf1c |  |  |  | Eif4h |  |
|  | Ttc7a |  | Tanc1 |  |  |  | Eif5 |  |
|  | Ube2e3 |  | Tanc2 |  |  |  | Eif6 |  |
|  | Ube2i |  | Tank |  |  |  | Elavl1 |  |
|  | Ube2j2 |  | Tbc1d1 |  |  |  | Elk3 |  |
|  | Ube3c |  | Tcerg1 |  |  |  | Elovl5 |  |
|  | Ube4b |  | Tcf4 |  |  |  | Emc10 |  |
|  | Ublcp1 |  | Tead4 |  |  |  | Emc3 |  |
|  | Ubp1 |  | Thbs3 |  |  |  | Emd |  |
|  | Ubqln1 |  | Thrap3 |  |  |  | Eme2 |  |
|  | Ubqln4 |  | Timm17b |  |  |  | Emilin1 |  |
|  | Ubr2 |  | Tmem131 |  |  |  | Enah |  |
|  | Ubr4 |  | Tmem14c |  |  |  | Eng |  |
|  | Ubr5 |  | Tmem159 |  |  |  | Eno2 |  |
|  | Unc50 |  | Tmem176b |  |  |  | Enpp1 |  |
|  | Usf1 |  | Tmem38b |  |  |  | Ep300 |  |
|  | Usp19 |  | Tmem5 |  |  |  | Epc2 |  |
|  | Usp24 |  | Tmem50b |  |  |  | Epdr1 |  |
|  | Usp33 |  | Tmem9b |  |  |  | Ephb4 |  |
|  | Usp36 |  | Tmsb10 |  |  |  | Eps8 |  |
|  | Usp4 |  | Tnnt2 |  |  |  | Erap1 |  |
|  | Vars |  | Tnrc6b |  |  |  | Ergic1 |  |
|  | Vav2 |  | Tomm34 |  |  |  | Eri3 |  |
|  | Vcan |  | Top3b |  |  |  | Ero1l |  |
|  | Vgll3 |  | Tpp2 |  |  |  | Esyt1 |  |
|  | Vwa5a |  | Tprkb |  |  |  | Esyt2 |  |
|  | Wac |  | Trim41 |  |  |  | Etnk1 |  |
|  | Wbscr17 |  | Tsc1 |  |  |  | Ets1 |  |
|  | Wdfy3 |  | Ubap2l |  |  |  | Ewsr1 |  |
|  | Wdtc1 |  | Ube2q2 |  |  |  | Exoc2 |  |
|  | Wisp1 |  | Ubr3 |  |  |  | Ext2 |  |
|  | Wsb1 |  | Ubxn2a |  |  |  | F2r |  |
|  | Xiap |  | Uhrf1bp1l |  |  |  | Faf2 |  |
|  | Xrn2 |  | Uhrf2 |  |  |  | Fam102b |  |
|  | Yap1 |  | Vangl1 |  |  |  | Fam129a |  |
|  | Yeats2 |  | Vasp |  |  |  | Fam129b |  |
|  | Yes1 |  | Vcpkmt |  |  |  | Fam160b1 |  |
|  | Zbtb44 |  | Vps16 |  |  |  | Fam168a |  |
|  | Zbtb47 |  | Vps41 |  |  |  | Fam168b |  |
|  | Zc3h4 |  | Vps54 |  |  |  | Fam171a1 |  |
|  | Zc3h7a |  | Vstm4 |  |  |  | Fam171a2 |  |
|  | Zcchc14 |  | Vwa8 |  |  |  | Fam206a |  |
|  | Zfc3h1 |  | Wdr54 |  |  |  | Fam20c |  |
|  | Zfp496 |  | Wdr7 |  |  |  | Fam46a |  |
|  | Zfp598 |  | Wfs1 |  |  |  | Fam49b |  |
|  | Znrf2 |  | Wt1 |  |  |  | Fam65a |  |
|  | Zswim8 |  | Xpnpep1 |  |  |  | Fam89b |  |
|  | March6 |  | Xpot |  |  |  | Fam8a1 |  |
|  | AABR06033633.1 |  | Zc3h11a |  |  |  | Fam96b |  |
|  | AABR06056275.2 |  | Zcchc17 |  |  |  | Farsa |  |
|  | AABR06077389.1 |  | Zcchc6 |  |  |  | Fasn |  |
|  |  |  | Zdhhc18 |  |  |  | Fat1 |  |
|  |  |  | Zfand2a |  |  |  | Fbln2 |  |
|  |  |  | Zfand2b |  |  |  | Fbln5 |  |
|  |  |  | Zfp207 |  |  |  | Fbn1 |  |
|  |  |  | Zfp266 |  |  |  | Fbrs |  |
|  |  |  | Zfp280d |  |  |  | Fbxl17 |  |
|  |  |  | Zfp295 |  |  |  | Fbxl5 |  |
|  |  |  | Zfp384 |  |  |  | Fbxl7 |  |
|  |  |  | Zfp638 |  |  |  | Fbxo11 |  |
|  |  |  | Zfp869 |  |  |  | Fbxo28 |  |
|  |  |  | Zmynd11 |  |  |  | Fbxo32 |  |
|  |  |  | Zmynd19 |  |  |  | Fbxw11 |  |
|  |  |  | Zmynd8 |  |  |  | Fbxw2 |  |
|  |  |  | Wiz |  |  |  | Fbxw4 |  |
|  |  |  | AABR06042582.1 |  |  |  | Fdx1 |  |
|  |  |  | AABR06042584.1 |  |  |  | Fem1b |  |
|  |  |  | AABR06064747.1 |  |  |  | Fermt2 |  |
|  |  |  | AABR06078876.3 |  |  |  | Fgfr1op |  |
|  |  |  | AABR06078887.1 |  |  |  | Fgfr2 |  |
|  |  |  |  |  |  |  | Fhl2 |  |
|  |  |  |  |  |  |  | Fhl3 |  |
|  |  |  |  |  |  |  | Fibin |  |
|  |  |  |  |  |  |  | Fkbp1a |  |
|  |  |  |  |  |  |  | Fkbp7 |  |
|  |  |  |  |  |  |  | Flii |  |
|  |  |  |  |  |  |  | Flot2 |  |
|  |  |  |  |  |  |  | Fmnl3 |  |
|  |  |  |  |  |  |  | Fmod |  |
|  |  |  |  |  |  |  | Fndc3a |  |
|  |  |  |  |  |  |  | Fosl2 |  |
|  |  |  |  |  |  |  | Foxk1 |  |
|  |  |  |  |  |  |  | Foxn2 |  |
|  |  |  |  |  |  |  | Foxn3 |  |
|  |  |  |  |  |  |  | Foxo3 |  |
|  |  |  |  |  |  |  | Frmd4a |  |
|  |  |  |  |  |  |  | Frrs1 |  |
|  |  |  |  |  |  |  | Fstl1 |  |
|  |  |  |  |  |  |  | Ftl1 |  |
|  |  |  |  |  |  |  | Fubp1 |  |
|  |  |  |  |  |  |  | Fuca1 |  |
|  |  |  |  |  |  |  | Furin |  |
|  |  |  |  |  |  |  | Fus |  |
|  |  |  |  |  |  |  | Fxr2 |  |
|  |  |  |  |  |  |  | Fyn |  |
|  |  |  |  |  |  |  | Fzd1 |  |
|  |  |  |  |  |  |  | Fzd2 |  |
|  |  |  |  |  |  |  | G3bp1 |  |
|  |  |  |  |  |  |  | G6pc3 |  |
|  |  |  |  |  |  |  | Gaa |  |
|  |  |  |  |  |  |  | Gab2 |  |
|  |  |  |  |  |  |  | Gabarapl1 |  |
|  |  |  |  |  |  |  | Gabarapl2 |  |
|  |  |  |  |  |  |  | Gabbr1 |  |
|  |  |  |  |  |  |  | Gabpa |  |
|  |  |  |  |  |  |  | Gadd45a |  |
|  |  |  |  |  |  |  | Gadd45b |  |
|  |  |  |  |  |  |  | Gak |  |
|  |  |  |  |  |  |  | Gale |  |
|  |  |  |  |  |  |  | Galk2 |  |
|  |  |  |  |  |  |  | Galnt1 |  |
|  |  |  |  |  |  |  | Galnt10 |  |
|  |  |  |  |  |  |  | Galnt2 |  |
|  |  |  |  |  |  |  | Gas6 |  |
|  |  |  |  |  |  |  | Gata4 |  |
|  |  |  |  |  |  |  | Gata6 |  |
|  |  |  |  |  |  |  | Gatad2b |  |
|  |  |  |  |  |  |  | Gbp2 |  |
|  |  |  |  |  |  |  | Gclm |  |
|  |  |  |  |  |  |  | Get4 |  |
|  |  |  |  |  |  |  | Gfpt1 |  |
|  |  |  |  |  |  |  | Gga2 |  |
|  |  |  |  |  |  |  | Ginm1 |  |
|  |  |  |  |  |  |  | Git1 |  |
|  |  |  |  |  |  |  | Gja1 |  |
|  |  |  |  |  |  |  | Gja5 |  |
|  |  |  |  |  |  |  | Glb1l |  |
|  |  |  |  |  |  |  | Glg1 |  |
|  |  |  |  |  |  |  | Gli3 |  |
|  |  |  |  |  |  |  | Glipr1 |  |
|  |  |  |  |  |  |  | Glis2 |  |
|  |  |  |  |  |  |  | Glud1 |  |
|  |  |  |  |  |  |  | Glul |  |
|  |  |  |  |  |  |  | Gm20721 |  |
|  |  |  |  |  |  |  | Gmfb |  |
|  |  |  |  |  |  |  | Gna11 |  |
|  |  |  |  |  |  |  | Gna12 |  |
|  |  |  |  |  |  |  | Gna13 |  |
|  |  |  |  |  |  |  | Gnai3 |  |
|  |  |  |  |  |  |  | Gnaq |  |
|  |  |  |  |  |  |  | Gnas |  |
|  |  |  |  |  |  |  | Gnb1 |  |
|  |  |  |  |  |  |  | Gng8 |  |
|  |  |  |  |  |  |  | Gnl1 |  |
|  |  |  |  |  |  |  | Gnptab |  |
|  |  |  |  |  |  |  | Gns |  |
|  |  |  |  |  |  |  | Golga7 |  |
|  |  |  |  |  |  |  | Golph3 |  |
|  |  |  |  |  |  |  | Golt1b |  |
|  |  |  |  |  |  |  | Gorasp2 |  |
|  |  |  |  |  |  |  | Gosr2 |  |
|  |  |  |  |  |  |  | Gpatch8 |  |
|  |  |  |  |  |  |  | Gpbp1l1 |  |
|  |  |  |  |  |  |  | Gpc1 |  |
|  |  |  |  |  |  |  | Gpc4 |  |
|  |  |  |  |  |  |  | Gpi |  |
|  |  |  |  |  |  |  | Gpm6b |  |
|  |  |  |  |  |  |  | Gpr107 |  |
|  |  |  |  |  |  |  | Gpr108 |  |
|  |  |  |  |  |  |  | Gpr124 |  |
|  |  |  |  |  |  |  | Gpr176 |  |
|  |  |  |  |  |  |  | Gpr89b |  |
|  |  |  |  |  |  |  | Gpx8 |  |
|  |  |  |  |  |  |  | Gramd1a |  |
|  |  |  |  |  |  |  | Gramd4 |  |
|  |  |  |  |  |  |  | Grb10 |  |
|  |  |  |  |  |  |  | Grb14 |  |
|  |  |  |  |  |  |  | Grem1 |  |
|  |  |  |  |  |  |  | Grina |  |
|  |  |  |  |  |  |  | Grk6 |  |
|  |  |  |  |  |  |  | Grn |  |
|  |  |  |  |  |  |  | Gsap |  |
|  |  |  |  |  |  |  | Gsdmd |  |
|  |  |  |  |  |  |  | Gsk3b |  |
|  |  |  |  |  |  |  | Gsn |  |
|  |  |  |  |  |  |  | Gsr |  |
|  |  |  |  |  |  |  | Gsta1 |  |
|  |  |  |  |  |  |  | Gstm5 |  |
|  |  |  |  |  |  |  | Gtf2h1 |  |
|  |  |  |  |  |  |  | Gtf2ird1 |  |
|  |  |  |  |  |  |  | Gulp1 |  |
|  |  |  |  |  |  |  | Gys1 |  |
|  |  |  |  |  |  |  | H3f3b |  |
|  |  |  |  |  |  |  | H3f3c |  |
|  |  |  |  |  |  |  | Haus7 |  |
|  |  |  |  |  |  |  | Hdac5 |  |
|  |  |  |  |  |  |  | Hdgfrp2 |  |
|  |  |  |  |  |  |  | Helz |  |
|  |  |  |  |  |  |  | Herpud2 |  |
|  |  |  |  |  |  |  | Hes1 |  |
|  |  |  |  |  |  |  | Hexa |  |
|  |  |  |  |  |  |  | Hgs |  |
|  |  |  |  |  |  |  | Hiat1 |  |
|  |  |  |  |  |  |  | Higd1a |  |
|  |  |  |  |  |  |  | Hip1 |  |
|  |  |  |  |  |  |  | Hipk2 |  |
|  |  |  |  |  |  |  | Hipk3 |  |
|  |  |  |  |  |  |  | Hira |  |
|  |  |  |  |  |  |  | Hist1h4b |  |
|  |  |  |  |  |  |  | Hk1 |  |
|  |  |  |  |  |  |  | Hm13 |  |
|  |  |  |  |  |  |  | Hnrnpa0 |  |
|  |  |  |  |  |  |  | Hnrnpll |  |
|  |  |  |  |  |  |  | Hnrnpr |  |
|  |  |  |  |  |  |  | Hnrnpu |  |
|  |  |  |  |  |  |  | Hnrnpul2 |  |
|  |  |  |  |  |  |  | Hnrpd |  |
|  |  |  |  |  |  |  | Homer3 |  |
|  |  |  |  |  |  |  | Hprt1 |  |
|  |  |  |  |  |  |  | Hs6st1 |  |
|  |  |  |  |  |  |  | Hs6st2 |  |
|  |  |  |  |  |  |  | Hsf2 |  |
|  |  |  |  |  |  |  | Hspa13 |  |
|  |  |  |  |  |  |  | Hspb6 |  |
|  |  |  |  |  |  |  | Hspb8 |  |
|  |  |  |  |  |  |  | Hspd1 |  |
|  |  |  |  |  |  |  | Hsph1 |  |
|  |  |  |  |  |  |  | Htra1 |  |
|  |  |  |  |  |  |  | Icam1 |  |
|  |  |  |  |  |  |  | Icmt |  |
|  |  |  |  |  |  |  | Ids |  |
|  |  |  |  |  |  |  | Ier5l |  |
|  |  |  |  |  |  |  | Ifi27l2b |  |
|  |  |  |  |  |  |  | Ifit2 |  |
|  |  |  |  |  |  |  | Ifitm1 |  |
|  |  |  |  |  |  |  | Ifitm2 |  |
|  |  |  |  |  |  |  | Ifngr1 |  |
|  |  |  |  |  |  |  | Igf1r |  |
|  |  |  |  |  |  |  | Igf2bp2 |  |
|  |  |  |  |  |  |  | Igfbp4 |  |
|  |  |  |  |  |  |  | Igfbp7 |  |
|  |  |  |  |  |  |  | Ikbkap |  |
|  |  |  |  |  |  |  | Il15 |  |
|  |  |  |  |  |  |  | Il17ra |  |
|  |  |  |  |  |  |  | Il1rl1 |  |
|  |  |  |  |  |  |  | Il1rl2 |  |
|  |  |  |  |  |  |  | Il33 |  |
|  |  |  |  |  |  |  | **Il6st** |  |
|  |  |  |  |  |  |  | Imp4 |  |
|  |  |  |  |  |  |  | Impad1 |  |
|  |  |  |  |  |  |  | Impdh1 |  |
|  |  |  |  |  |  |  | INF2 |  |
|  |  |  |  |  |  |  | Inpp5a |  |
|  |  |  |  |  |  |  | Inppl1 |  |
|  |  |  |  |  |  |  | Ipo4 |  |
|  |  |  |  |  |  |  | Irak3 |  |
|  |  |  |  |  |  |  | Ireb2 |  |
|  |  |  |  |  |  |  | Irf2 |  |
|  |  |  |  |  |  |  | Irf2bp2 |  |
|  |  |  |  |  |  |  | Irf9 |  |
|  |  |  |  |  |  |  | Ist1 |  |
|  |  |  |  |  |  |  | Itfg1 |  |
|  |  |  |  |  |  |  | Itga11 |  |
|  |  |  |  |  |  |  | Itga5 |  |
|  |  |  |  |  |  |  | Itgav |  |
|  |  |  |  |  |  |  | Itgb5 |  |
|  |  |  |  |  |  |  | Itm2b |  |
|  |  |  |  |  |  |  | Itm2c |  |
|  |  |  |  |  |  |  | Ivd |  |
|  |  |  |  |  |  |  | Ivns1abp |  |
|  |  |  |  |  |  |  | Jam3 |  |
|  |  |  |  |  |  |  | Jkamp |  |
|  |  |  |  |  |  |  | Jph2 |  |
|  |  |  |  |  |  |  | Jun |  |
|  |  |  |  |  |  |  | Junb |  |
|  |  |  |  |  |  |  | Jund |  |
|  |  |  |  |  |  |  | Jup |  |
|  |  |  |  |  |  |  | Kank2 |  |
|  |  |  |  |  |  |  | Kansl2 |  |
|  |  |  |  |  |  |  | Kat5 |  |
|  |  |  |  |  |  |  | Katnal1 |  |
|  |  |  |  |  |  |  | Kctd10 |  |
|  |  |  |  |  |  |  | Kctd15 |  |
|  |  |  |  |  |  |  | Kctd20 |  |
|  |  |  |  |  |  |  | Kdelr2 |  |
|  |  |  |  |  |  |  | Kdm2a |  |
|  |  |  |  |  |  |  | Kdm4a |  |
|  |  |  |  |  |  |  | Kdm6b |  |
|  |  |  |  |  |  |  | Kdsr |  |
|  |  |  |  |  |  |  | Khdrbs1 |  |
|  |  |  |  |  |  |  | Khsrp |  |
|  |  |  |  |  |  |  | Kif1c |  |
|  |  |  |  |  |  |  | Kif21b |  |
|  |  |  |  |  |  |  | Kif2a |  |
|  |  |  |  |  |  |  | Kif5c |  |
|  |  |  |  |  |  |  | Kifap3 |  |
|  |  |  |  |  |  |  | Kirrel |  |
|  |  |  |  |  |  |  | Klf12 |  |
|  |  |  |  |  |  |  | Klf4 |  |
|  |  |  |  |  |  |  | Klhdc10 |  |
|  |  |  |  |  |  |  | Klhdc3 |  |
|  |  |  |  |  |  |  | Klhl2 |  |
|  |  |  |  |  |  |  | Klhl22 |  |
|  |  |  |  |  |  |  | Klhl29 |  |
|  |  |  |  |  |  |  | Kmt2e |  |
|  |  |  |  |  |  |  | Kpna3 |  |
|  |  |  |  |  |  |  | Kpnb1 |  |
|  |  |  |  |  |  |  | Ksr1 |  |
|  |  |  |  |  |  |  | Kxd1 |  |
|  |  |  |  |  |  |  | Lage3 |  |
|  |  |  |  |  |  |  | Lamb1 |  |
|  |  |  |  |  |  |  | Lamc1 |  |
|  |  |  |  |  |  |  | Lamp1 |  |
|  |  |  |  |  |  |  | Lamp2 |  |
|  |  |  |  |  |  |  | Laptm4b |  |
|  |  |  |  |  |  |  | Large |  |
|  |  |  |  |  |  |  | Larp1 |  |
|  |  |  |  |  |  |  | Larp4b |  |
|  |  |  |  |  |  |  | Lats1 |  |
|  |  |  |  |  |  |  | Lats2 |  |
|  |  |  |  |  |  |  | Ldb1 |  |
|  |  |  |  |  |  |  | Ldha |  |
|  |  |  |  |  |  |  | Ldhb |  |
|  |  |  |  |  |  |  | Ldlrad3 |  |
|  |  |  |  |  |  |  | Leng8 |  |
|  |  |  |  |  |  |  | Lepre1 |  |
|  |  |  |  |  |  |  | Leprel1 |  |
|  |  |  |  |  |  |  | Leprel2 |  |
|  |  |  |  |  |  |  | Leprel4 |  |
|  |  |  |  |  |  |  | Lgals3bp |  |
|  |  |  |  |  |  |  | Lgalsl |  |
|  |  |  |  |  |  |  | Lhfp |  |
|  |  |  |  |  |  |  | **Lif** |  |
|  |  |  |  |  |  |  | Lims1 |  |
|  |  |  |  |  |  |  | Litaf |  |
|  |  |  |  |  |  |  | Lix1l |  |
|  |  |  |  |  |  |  | Lmcd1 |  |
|  |  |  |  |  |  |  | Lmnb1 |  |
|  |  |  |  |  |  |  | Lmo4 |  |
|  |  |  |  |  |  |  | Lnpep |  |
|  |  |  |  |  |  |  | LOC100359421 |  |
|  |  |  |  |  |  |  | LOC100360368 |  |
|  |  |  |  |  |  |  | LOC100361645 |  |
|  |  |  |  |  |  |  | LOC100361830 |  |
|  |  |  |  |  |  |  | LOC100362038 |  |
|  |  |  |  |  |  |  | LOC100362065 |  |
|  |  |  |  |  |  |  | LOC100363110 |  |
|  |  |  |  |  |  |  | LOC100363915 |  |
|  |  |  |  |  |  |  | LOC100364500 |  |
|  |  |  |  |  |  |  | LOC100365902 |  |
|  |  |  |  |  |  |  | LOC100909544 |  |
|  |  |  |  |  |  |  | LOC100909548 |  |
|  |  |  |  |  |  |  | LOC100909712 |  |
|  |  |  |  |  |  |  | LOC100909840 |  |
|  |  |  |  |  |  |  | LOC100910196 |  |
|  |  |  |  |  |  |  | LOC100910646 |  |
|  |  |  |  |  |  |  | LOC100910750 |  |
|  |  |  |  |  |  |  | LOC100910771 |  |
|  |  |  |  |  |  |  | LOC100910831 |  |
|  |  |  |  |  |  |  | LOC100910875 |  |
|  |  |  |  |  |  |  | LOC100910881 |  |
|  |  |  |  |  |  |  | LOC100910882 |  |
|  |  |  |  |  |  |  | LOC100910990 |  |
|  |  |  |  |  |  |  | LOC100911178 |  |
|  |  |  |  |  |  |  | LOC100911186 |  |
|  |  |  |  |  |  |  | LOC100911319 |  |
|  |  |  |  |  |  |  | LOC100911356 |  |
|  |  |  |  |  |  |  | LOC100911374 |  |
|  |  |  |  |  |  |  | LOC100911485 |  |
|  |  |  |  |  |  |  | LOC100911498 |  |
|  |  |  |  |  |  |  | LOC100911545 |  |
|  |  |  |  |  |  |  | LOC100911550 |  |
|  |  |  |  |  |  |  | LOC100911588 |  |
|  |  |  |  |  |  |  | LOC100911664 |  |
|  |  |  |  |  |  |  | LOC100911672 |  |
|  |  |  |  |  |  |  | LOC100911730 |  |
|  |  |  |  |  |  |  | LOC100911766 |  |
|  |  |  |  |  |  |  | LOC100911993 |  |
|  |  |  |  |  |  |  | LOC100912380 |  |
|  |  |  |  |  |  |  | LOC100912481 |  |
|  |  |  |  |  |  |  | LOC100912571 |  |
|  |  |  |  |  |  |  | LOC100912604 |  |
|  |  |  |  |  |  |  | LOC100913000 |  |
|  |  |  |  |  |  |  | LOC102550189 |  |
|  |  |  |  |  |  |  | LOC102554884 |  |
|  |  |  |  |  |  |  | LOC294154 |  |
|  |  |  |  |  |  |  | LOC306766 |  |
|  |  |  |  |  |  |  | LOC678813 |  |
|  |  |  |  |  |  |  | LOC680227 |  |
|  |  |  |  |  |  |  | LOC681193 |  |
|  |  |  |  |  |  |  | LOC683565 |  |
|  |  |  |  |  |  |  | LOC684934 |  |
|  |  |  |  |  |  |  | LOC686349 |  |
|  |  |  |  |  |  |  | LOC686736 |  |
|  |  |  |  |  |  |  | LOC687994 |  |
|  |  |  |  |  |  |  | LOC689574 |  |
|  |  |  |  |  |  |  | Lox |  |
|  |  |  |  |  |  |  | Loxl1 |  |
|  |  |  |  |  |  |  | Lpcat1 |  |
|  |  |  |  |  |  |  | Lpcat3 |  |
|  |  |  |  |  |  |  | Lpgat1 |  |
|  |  |  |  |  |  |  | Lphn1 |  |
|  |  |  |  |  |  |  | Lrch1 |  |
|  |  |  |  |  |  |  | Lrp1 |  |
|  |  |  |  |  |  |  | Lrp6 |  |
|  |  |  |  |  |  |  | Lrrc16a |  |
|  |  |  |  |  |  |  | Lrrc32 |  |
|  |  |  |  |  |  |  | Lrrc41 |  |
|  |  |  |  |  |  |  | Lrrc58 |  |
|  |  |  |  |  |  |  | Lsm12 |  |
|  |  |  |  |  |  |  | Lsm14a |  |
|  |  |  |  |  |  |  | Lss |  |
|  |  |  |  |  |  |  | Ltbp1 |  |
|  |  |  |  |  |  |  | Ltbp2 |  |
|  |  |  |  |  |  |  | Ltbp3 |  |
|  |  |  |  |  |  |  | Ltbr |  |
|  |  |  |  |  |  |  | Luc7l |  |
|  |  |  |  |  |  |  | Luc7l2 |  |
|  |  |  |  |  |  |  | Luc7l3 |  |
|  |  |  |  |  |  |  | Ly6e |  |
|  |  |  |  |  |  |  | Lypd1 |  |
|  |  |  |  |  |  |  | Lypla1 |  |
|  |  |  |  |  |  |  | Lypla2 |  |
|  |  |  |  |  |  |  | Maea |  |
|  |  |  |  |  |  |  | Maml2 |  |
|  |  |  |  |  |  |  | Man1b1 |  |
|  |  |  |  |  |  |  | Man2a1 |  |
|  |  |  |  |  |  |  | Man2b1 |  |
|  |  |  |  |  |  |  | Man2c1 |  |
|  |  |  |  |  |  |  | Map1lc3a |  |
|  |  |  |  |  |  |  | Map2k1 |  |
|  |  |  |  |  |  |  | Map2k4 |  |
|  |  |  |  |  |  |  | Map2k5 |  |
|  |  |  |  |  |  |  | Map2k7 |  |
|  |  |  |  |  |  |  | Map3k2 |  |
|  |  |  |  |  |  |  | Map3k3 |  |
|  |  |  |  |  |  |  | Map3k7 |  |
|  |  |  |  |  |  |  | Map4 |  |
|  |  |  |  |  |  |  | Map4k5 |  |
|  |  |  |  |  |  |  | Mapk14 |  |
|  |  |  |  |  |  |  | Mapk1ip1l |  |
|  |  |  |  |  |  |  | Mapk3 |  |
|  |  |  |  |  |  |  | Mapk6 |  |
|  |  |  |  |  |  |  | Mapk8ip3 |  |
|  |  |  |  |  |  |  | Mapkapk2 |  |
|  |  |  |  |  |  |  | Mapkapk5 |  |
|  |  |  |  |  |  |  | March2 |  |
|  |  |  |  |  |  |  | March3 |  |
|  |  |  |  |  |  |  | March5 |  |
|  |  |  |  |  |  |  | March7 |  |
|  |  |  |  |  |  |  | Marcksl1 |  |
|  |  |  |  |  |  |  | Marf1 |  |
|  |  |  |  |  |  |  | Mark1 |  |
|  |  |  |  |  |  |  | Mark4 |  |
|  |  |  |  |  |  |  | Marveld1 |  |
|  |  |  |  |  |  |  | Mast2 |  |
|  |  |  |  |  |  |  | Mat2b |  |
|  |  |  |  |  |  |  | Mau2 |  |
|  |  |  |  |  |  |  | Max |  |
|  |  |  |  |  |  |  | Maz |  |
|  |  |  |  |  |  |  | Mbd1 |  |
|  |  |  |  |  |  |  | Mbd6 |  |
|  |  |  |  |  |  |  | Mbnl2 |  |
|  |  |  |  |  |  |  | Mcart1 |  |
|  |  |  |  |  |  |  | Mcm2 |  |
|  |  |  |  |  |  |  | Mcm4 |  |
|  |  |  |  |  |  |  | Mcm6 |  |
|  |  |  |  |  |  |  | Mcu |  |
|  |  |  |  |  |  |  | Mcur1 |  |
|  |  |  |  |  |  |  | Mdm2 |  |
|  |  |  |  |  |  |  | Me1 |  |
|  |  |  |  |  |  |  | Meaf6 |  |
|  |  |  |  |  |  |  | Med13 |  |
|  |  |  |  |  |  |  | Med14 |  |
|  |  |  |  |  |  |  | Med15 |  |
|  |  |  |  |  |  |  | Med21 |  |
|  |  |  |  |  |  |  | Med25 |  |
|  |  |  |  |  |  |  | Mef2d |  |
|  |  |  |  |  |  |  | Meis2 |  |
|  |  |  |  |  |  |  | Memo1 |  |
|  |  |  |  |  |  |  | Metrnl |  |
|  |  |  |  |  |  |  | Mex3c |  |
|  |  |  |  |  |  |  | Mex3d |  |
|  |  |  |  |  |  |  | Mfge8 |  |
|  |  |  |  |  |  |  | Mfn1 |  |
|  |  |  |  |  |  |  | Mfsd1 |  |
|  |  |  |  |  |  |  | Mfsd5 |  |
|  |  |  |  |  |  |  | Mgat4b |  |
|  |  |  |  |  |  |  | MGC108823 |  |
|  |  |  |  |  |  |  | MGC112830 |  |
|  |  |  |  |  |  |  | MGC94335 |  |
|  |  |  |  |  |  |  | Mgea5 |  |
|  |  |  |  |  |  |  | Mib1 |  |
|  |  |  |  |  |  |  | Mib2 |  |
|  |  |  |  |  |  |  | Micu2 |  |
|  |  |  |  |  |  |  | Mif |  |
|  |  |  |  |  |  |  | Mink1 |  |
|  |  |  |  |  |  |  | Minpp1 |  |
|  |  |  |  |  |  |  | Mitd1 |  |
|  |  |  |  |  |  |  | Mkln1 |  |
|  |  |  |  |  |  |  | Mknk2 |  |
|  |  |  |  |  |  |  | Mlec |  |
|  |  |  |  |  |  |  | Mllt4 |  |
|  |  |  |  |  |  |  | Mnda |  |
|  |  |  |  |  |  |  | Mob1a |  |
|  |  |  |  |  |  |  | Mob2 |  |
|  |  |  |  |  |  |  | Mob3a |  |
|  |  |  |  |  |  |  | Mon2 |  |
|  |  |  |  |  |  |  | Mpv17 |  |
|  |  |  |  |  |  |  | Mpv17l2 |  |
|  |  |  |  |  |  |  | Mroh1 |  |
|  |  |  |  |  |  |  | Mrpl1 |  |
|  |  |  |  |  |  |  | Mrpl17 |  |
|  |  |  |  |  |  |  | Mrpl43 |  |
|  |  |  |  |  |  |  | Msi2 |  |
|  |  |  |  |  |  |  | Msl1 |  |
|  |  |  |  |  |  |  | Msl2 |  |
|  |  |  |  |  |  |  | Msmp |  |
|  |  |  |  |  |  |  | Mta1 |  |
|  |  |  |  |  |  |  | Mtap |  |
|  |  |  |  |  |  |  | Mtfr1 |  |
|  |  |  |  |  |  |  | Mthfd2 |  |
|  |  |  |  |  |  |  | Mtss1l |  |
|  |  |  |  |  |  |  | Mvb12b |  |
|  |  |  |  |  |  |  | Mvp |  |
|  |  |  |  |  |  |  | Mx1 |  |
|  |  |  |  |  |  |  | Mx2 |  |
|  |  |  |  |  |  |  | Mxd4 |  |
|  |  |  |  |  |  |  | Mxra8 |  |
|  |  |  |  |  |  |  | Myadm |  |
|  |  |  |  |  |  |  | Myc |  |
|  |  |  |  |  |  |  | Myh6 |  |
|  |  |  |  |  |  |  | Myh7 |  |
|  |  |  |  |  |  |  | Myl12b |  |
|  |  |  |  |  |  |  | Myo1d |  |
|  |  |  |  |  |  |  | Myof |  |
|  |  |  |  |  |  |  | Myzap |  |
|  |  |  |  |  |  |  | N4bp1 |  |
|  |  |  |  |  |  |  | Naa60 |  |
|  |  |  |  |  |  |  | Nadk |  |
|  |  |  |  |  |  |  | Nampt |  |
|  |  |  |  |  |  |  | Nanp |  |
|  |  |  |  |  |  |  | Nap1l4 |  |
|  |  |  |  |  |  |  | Napa |  |
|  |  |  |  |  |  |  | Ncam1 |  |
|  |  |  |  |  |  |  | Ncbp1 |  |
|  |  |  |  |  |  |  | Nckap1 |  |
|  |  |  |  |  |  |  | Ncln |  |
|  |  |  |  |  |  |  | Ncoa4 |  |
|  |  |  |  |  |  |  | Ncstn |  |
|  |  |  |  |  |  |  | Ndel1 |  |
|  |  |  |  |  |  |  | Ndfip2 |  |
|  |  |  |  |  |  |  | Ndufa10 |  |
|  |  |  |  |  |  |  | Ndufa2 |  |
|  |  |  |  |  |  |  | Ndufa7 |  |
|  |  |  |  |  |  |  | Nedd4 |  |
|  |  |  |  |  |  |  | Nedd9 |  |
|  |  |  |  |  |  |  | Nek6 |  |
|  |  |  |  |  |  |  | Nek9 |  |
|  |  |  |  |  |  |  | Neu1 |  |
|  |  |  |  |  |  |  | Nf1 |  |
|  |  |  |  |  |  |  | Nfatc3 |  |
|  |  |  |  |  |  |  | Nfe2l1 |  |
|  |  |  |  |  |  |  | Nfe2l2 |  |
|  |  |  |  |  |  |  | Nfia |  |
|  |  |  |  |  |  |  | Nfkbia |  |
|  |  |  |  |  |  |  | Nfx1 |  |
|  |  |  |  |  |  |  | Nhlrc3 |  |
|  |  |  |  |  |  |  | Nhp2l1 |  |
|  |  |  |  |  |  |  | Nhs |  |
|  |  |  |  |  |  |  | Nid2 |  |
|  |  |  |  |  |  |  | Nifk |  |
|  |  |  |  |  |  |  | Ninj1 |  |
|  |  |  |  |  |  |  | Nisch |  |
|  |  |  |  |  |  |  | Nkain1 |  |
|  |  |  |  |  |  |  | Nkd2 |  |
|  |  |  |  |  |  |  | Nktr |  |
|  |  |  |  |  |  |  | Nnt |  |
|  |  |  |  |  |  |  | Noc2l |  |
|  |  |  |  |  |  |  | Nolc1 |  |
|  |  |  |  |  |  |  | Nomo1 |  |
|  |  |  |  |  |  |  | Notch2 |  |
|  |  |  |  |  |  |  | Nov |  |
|  |  |  |  |  |  |  | Npdc1 |  |
|  |  |  |  |  |  |  | Npepps |  |
|  |  |  |  |  |  |  | Nploc4 |  |
|  |  |  |  |  |  |  | Nppb |  |
|  |  |  |  |  |  |  | Nptn |  |
|  |  |  |  |  |  |  | Nr1d2 |  |
|  |  |  |  |  |  |  | Nr2f2 |  |
|  |  |  |  |  |  |  | Nr3c1 |  |
|  |  |  |  |  |  |  | Nradd |  |
|  |  |  |  |  |  |  | Nrep |  |
|  |  |  |  |  |  |  | Nsmaf |  |
|  |  |  |  |  |  |  | Nt5c2 |  |
|  |  |  |  |  |  |  | Nt5dc2 |  |
|  |  |  |  |  |  |  | Nt5dc3 |  |
|  |  |  |  |  |  |  | Nt5e |  |
|  |  |  |  |  |  |  | Ntm |  |
|  |  |  |  |  |  |  | Nuak2 |  |
|  |  |  |  |  |  |  | Nucb1 |  |
|  |  |  |  |  |  |  | Nudt3 |  |
|  |  |  |  |  |  |  | Nudt4 |  |
|  |  |  |  |  |  |  | Numa1 |  |
|  |  |  |  |  |  |  | Numb |  |
|  |  |  |  |  |  |  | Numbl |  |
|  |  |  |  |  |  |  | Nup153 |  |
|  |  |  |  |  |  |  | Nup62 |  |
|  |  |  |  |  |  |  | Nup98 |  |
|  |  |  |  |  |  |  | Nutf2 |  |
|  |  |  |  |  |  |  | Nxf1 |  |
|  |  |  |  |  |  |  | Oasl |  |
|  |  |  |  |  |  |  | Oasl2 |  |
|  |  |  |  |  |  |  | Oaz1 |  |
|  |  |  |  |  |  |  | Oaz2 |  |
|  |  |  |  |  |  |  | Ociad1 |  |
|  |  |  |  |  |  |  | Ogt |  |
|  |  |  |  |  |  |  | Olr1 |  |
|  |  |  |  |  |  |  | Orc2 |  |
|  |  |  |  |  |  |  | Oser1 |  |
|  |  |  |  |  |  |  | Ost4 |  |
|  |  |  |  |  |  |  | Otud1 |  |
|  |  |  |  |  |  |  | Otud4 |  |
|  |  |  |  |  |  |  | Oxa1l |  |
|  |  |  |  |  |  |  | Oxct1 |  |
|  |  |  |  |  |  |  | P4ha2 |  |
|  |  |  |  |  |  |  | P4ha3 |  |
|  |  |  |  |  |  |  | P4hb |  |
|  |  |  |  |  |  |  | Pabpc1 |  |
|  |  |  |  |  |  |  | Pafah1b2 |  |
|  |  |  |  |  |  |  | Paip2b |  |
|  |  |  |  |  |  |  | Pak1 |  |
|  |  |  |  |  |  |  | Pak2 |  |
|  |  |  |  |  |  |  | Pam16 |  |
|  |  |  |  |  |  |  | Papss1 |  |
|  |  |  |  |  |  |  | Papss2 |  |
|  |  |  |  |  |  |  | Pard3 |  |
|  |  |  |  |  |  |  | Parm1 |  |
|  |  |  |  |  |  |  | Parp12 |  |
|  |  |  |  |  |  |  | Parp4 |  |
|  |  |  |  |  |  |  | Parp6 |  |
|  |  |  |  |  |  |  | Parva |  |
|  |  |  |  |  |  |  | Paxbp1 |  |
|  |  |  |  |  |  |  | Pbx1 |  |
|  |  |  |  |  |  |  | Pbx3 |  |
|  |  |  |  |  |  |  | Pbxip1 |  |
|  |  |  |  |  |  |  | Pcbp4 |  |
|  |  |  |  |  |  |  | Pcdhga9 |  |
|  |  |  |  |  |  |  | Pcdhgb7 |  |
|  |  |  |  |  |  |  | Pcgf5 |  |
|  |  |  |  |  |  |  | Pck2 |  |
|  |  |  |  |  |  |  | Pcmt1 |  |
|  |  |  |  |  |  |  | Pcmtd1 |  |
|  |  |  |  |  |  |  | Pcolce |  |
|  |  |  |  |  |  |  | Pdcd6ip |  |
|  |  |  |  |  |  |  | Pde3b |  |
|  |  |  |  |  |  |  | Pde4b |  |
|  |  |  |  |  |  |  | Pde7a |  |
|  |  |  |  |  |  |  | Pdgfa |  |
|  |  |  |  |  |  |  | Pdgfc |  |
|  |  |  |  |  |  |  | Pdgfrb |  |
|  |  |  |  |  |  |  | Pdhb |  |
|  |  |  |  |  |  |  | Pdlim1 |  |
|  |  |  |  |  |  |  | Pdlim3 |  |
|  |  |  |  |  |  |  | Pdlim4 |  |
|  |  |  |  |  |  |  | Pdrg1 |  |
|  |  |  |  |  |  |  | Pds5a |  |
|  |  |  |  |  |  |  | Pdzrn3 |  |
|  |  |  |  |  |  |  | Peak1 |  |
|  |  |  |  |  |  |  | Peli1 |  |
|  |  |  |  |  |  |  | Pelo |  |
|  |  |  |  |  |  |  | Pfdn2 |  |
|  |  |  |  |  |  |  | Pfkl |  |
|  |  |  |  |  |  |  | Pfkp |  |
|  |  |  |  |  |  |  | Pfn2 |  |
|  |  |  |  |  |  |  | Pgam1 |  |
|  |  |  |  |  |  |  | Pgk1 |  |
|  |  |  |  |  |  |  | Pgls |  |
|  |  |  |  |  |  |  | Pgpep1 |  |
|  |  |  |  |  |  |  | Pgrmc1 |  |
|  |  |  |  |  |  |  | Phf12 |  |
|  |  |  |  |  |  |  | Phf2 |  |
|  |  |  |  |  |  |  | Phf20l1 |  |
|  |  |  |  |  |  |  | Phlda1 |  |
|  |  |  |  |  |  |  | Phlda3 |  |
|  |  |  |  |  |  |  | Phldb1 |  |
|  |  |  |  |  |  |  | Phldb3 |  |
|  |  |  |  |  |  |  | Phtf1 |  |
|  |  |  |  |  |  |  | Phyhipl |  |
|  |  |  |  |  |  |  | Pi4k2b |  |
|  |  |  |  |  |  |  | Pid1 |  |
|  |  |  |  |  |  |  | Piezo2 |  |
|  |  |  |  |  |  |  | Pigf |  |
|  |  |  |  |  |  |  | Pigk |  |
|  |  |  |  |  |  |  | Pigt |  |
|  |  |  |  |  |  |  | Pik3c2a |  |
|  |  |  |  |  |  |  | Pim1 |  |
|  |  |  |  |  |  |  | Pim3 |  |
|  |  |  |  |  |  |  | Pink1 |  |
|  |  |  |  |  |  |  | Pip5k1a |  |
|  |  |  |  |  |  |  | Pir |  |
|  |  |  |  |  |  |  | Pithd1 |  |
|  |  |  |  |  |  |  | Pitrm1 |  |
|  |  |  |  |  |  |  | Pja2 |  |
|  |  |  |  |  |  |  | Pkd2 |  |
|  |  |  |  |  |  |  | Pkm |  |
|  |  |  |  |  |  |  | Plaa |  |
|  |  |  |  |  |  |  | Plaur |  |
|  |  |  |  |  |  |  | Plbd2 |  |
|  |  |  |  |  |  |  | Plekha3 |  |
|  |  |  |  |  |  |  | Plekha7 |  |
|  |  |  |  |  |  |  | Plekhb2 |  |
|  |  |  |  |  |  |  | Plekhg2 |  |
|  |  |  |  |  |  |  | Plk2 |  |
|  |  |  |  |  |  |  | Plk3 |  |
|  |  |  |  |  |  |  | Plod1 |  |
|  |  |  |  |  |  |  | Plod2 |  |
|  |  |  |  |  |  |  | Plod3 |  |
|  |  |  |  |  |  |  | Pls3 |  |
|  |  |  |  |  |  |  | Plscr3 |  |
|  |  |  |  |  |  |  | Plxnb2 |  |
|  |  |  |  |  |  |  | Pmepa1 |  |
|  |  |  |  |  |  |  | Pmm1 |  |
|  |  |  |  |  |  |  | Pmp22 |  |
|  |  |  |  |  |  |  | Pmpca |  |
|  |  |  |  |  |  |  | Pmpcb |  |
|  |  |  |  |  |  |  | Pnisr |  |
|  |  |  |  |  |  |  | Pnn |  |
|  |  |  |  |  |  |  | Pnpla2 |  |
|  |  |  |  |  |  |  | Pnpt1 |  |
|  |  |  |  |  |  |  | Pnrc1 |  |
|  |  |  |  |  |  |  | Pofut2 |  |
|  |  |  |  |  |  |  | Poglut1 |  |
|  |  |  |  |  |  |  | Poldip2 |  |
|  |  |  |  |  |  |  | Polr3h |  |
|  |  |  |  |  |  |  | Pom121 |  |
|  |  |  |  |  |  |  | Postn |  |
|  |  |  |  |  |  |  | Ppap2a |  |
|  |  |  |  |  |  |  | Ppard |  |
|  |  |  |  |  |  |  | Pphln1 |  |
|  |  |  |  |  |  |  | Ppm1a |  |
|  |  |  |  |  |  |  | Ppm1b |  |
|  |  |  |  |  |  |  | Ppp1cb |  |
|  |  |  |  |  |  |  | Ppp1r12c |  |
|  |  |  |  |  |  |  | Ppp1r13l |  |
|  |  |  |  |  |  |  | Ppp1r15a |  |
|  |  |  |  |  |  |  | Ppp1r18 |  |
|  |  |  |  |  |  |  | Ppp2ca |  |
|  |  |  |  |  |  |  | Ppp2cb |  |
|  |  |  |  |  |  |  | Ppp2r2a |  |
|  |  |  |  |  |  |  | Ppp2r4 |  |
|  |  |  |  |  |  |  | Ppp2r5a |  |
|  |  |  |  |  |  |  | Ppp3ca |  |
|  |  |  |  |  |  |  | Ppp3r1 |  |
|  |  |  |  |  |  |  | Ppp6c |  |
|  |  |  |  |  |  |  | Ppp6r1 |  |
|  |  |  |  |  |  |  | Prcc |  |
|  |  |  |  |  |  |  | Prdx5 |  |
|  |  |  |  |  |  |  | Prdx6 |  |
|  |  |  |  |  |  |  | Prelp |  |
|  |  |  |  |  |  |  | Prep |  |
|  |  |  |  |  |  |  | Prickle1 |  |
|  |  |  |  |  |  |  | Prim1 |  |
|  |  |  |  |  |  |  | Prkaa1 |  |
|  |  |  |  |  |  |  | Prkacb |  |
|  |  |  |  |  |  |  | Prkag1 |  |
|  |  |  |  |  |  |  | Prkar1a |  |
|  |  |  |  |  |  |  | Prkar2a |  |
|  |  |  |  |  |  |  | Prkrip1 |  |
|  |  |  |  |  |  |  | Prnp |  |
|  |  |  |  |  |  |  | Pros1 |  |
|  |  |  |  |  |  |  | Prosc |  |
|  |  |  |  |  |  |  | Prpf18 |  |
|  |  |  |  |  |  |  | Prpf38b |  |
|  |  |  |  |  |  |  | Prpf4b |  |
|  |  |  |  |  |  |  | Prpf8 |  |
|  |  |  |  |  |  |  | Prrc1 |  |
|  |  |  |  |  |  |  | Prrc2a |  |
|  |  |  |  |  |  |  | Prrg4 |  |
|  |  |  |  |  |  |  | Prrx1 |  |
|  |  |  |  |  |  |  | Prss23 |  |
|  |  |  |  |  |  |  | Psap |  |
|  |  |  |  |  |  |  | Psen1 |  |
|  |  |  |  |  |  |  | Psen2 |  |
|  |  |  |  |  |  |  | Psmd11 |  |
|  |  |  |  |  |  |  | Psmd3 |  |
|  |  |  |  |  |  |  | Psmd9 |  |
|  |  |  |  |  |  |  | Psme4 |  |
|  |  |  |  |  |  |  | Psmf1 |  |
|  |  |  |  |  |  |  | Pspc1 |  |
|  |  |  |  |  |  |  | Ptbp2 |  |
|  |  |  |  |  |  |  | Ptbp3 |  |
|  |  |  |  |  |  |  | Ptdss1 |  |
|  |  |  |  |  |  |  | Pten |  |
|  |  |  |  |  |  |  | Ptger1 |  |
|  |  |  |  |  |  |  | Ptges3 |  |
|  |  |  |  |  |  |  | Ptgfrn |  |
|  |  |  |  |  |  |  | Ptgis |  |
|  |  |  |  |  |  |  | Ptk7 |  |
|  |  |  |  |  |  |  | Ptov1 |  |
|  |  |  |  |  |  |  | Ptp4a1 |  |
|  |  |  |  |  |  |  | Ptp4a2 |  |
|  |  |  |  |  |  |  | Ptpn1 |  |
|  |  |  |  |  |  |  | Ptpn11 |  |
|  |  |  |  |  |  |  | Ptpn12 |  |
|  |  |  |  |  |  |  | Ptpn14 |  |
|  |  |  |  |  |  |  | Ptpn23 |  |
|  |  |  |  |  |  |  | Ptpn6 |  |
|  |  |  |  |  |  |  | Ptprm |  |
|  |  |  |  |  |  |  | Ptprs |  |
|  |  |  |  |  |  |  | Ptrf |  |
|  |  |  |  |  |  |  | Purb |  |
|  |  |  |  |  |  |  | Pxdn |  |
|  |  |  |  |  |  |  | Pxk |  |
|  |  |  |  |  |  |  | Pxn |  |
|  |  |  |  |  |  |  | Qrich1 |  |
|  |  |  |  |  |  |  | Qsox1 |  |
|  |  |  |  |  |  |  | R3hdm4 |  |
|  |  |  |  |  |  |  | Rab1 |  |
|  |  |  |  |  |  |  | Rab11b |  |
|  |  |  |  |  |  |  | Rab11fip3 |  |
|  |  |  |  |  |  |  | Rab11fip5 |  |
|  |  |  |  |  |  |  | Rab14 |  |
|  |  |  |  |  |  |  | Rab18 |  |
|  |  |  |  |  |  |  | Rab22a |  |
|  |  |  |  |  |  |  | Rab23 |  |
|  |  |  |  |  |  |  | Rab24 |  |
|  |  |  |  |  |  |  | Rab2a |  |
|  |  |  |  |  |  |  | Rab2b |  |
|  |  |  |  |  |  |  | Rab32 |  |
|  |  |  |  |  |  |  | Rab35 |  |
|  |  |  |  |  |  |  | Rab3gap2 |  |
|  |  |  |  |  |  |  | Rab5al1 |  |
|  |  |  |  |  |  |  | Rab5b |  |
|  |  |  |  |  |  |  | Rab6a |  |
|  |  |  |  |  |  |  | Rab7a |  |
|  |  |  |  |  |  |  | Rac1 |  |
|  |  |  |  |  |  |  | Rae1 |  |
|  |  |  |  |  |  |  | Raf1 |  |
|  |  |  |  |  |  |  | Ranbp3 |  |
|  |  |  |  |  |  |  | Rap1a |  |
|  |  |  |  |  |  |  | Rap1b |  |
|  |  |  |  |  |  |  | Rap1gds1 |  |
|  |  |  |  |  |  |  | Rarg |  |
|  |  |  |  |  |  |  | Rasa1 |  |
|  |  |  |  |  |  |  | Rasl12 |  |
|  |  |  |  |  |  |  | Raver1 |  |
|  |  |  |  |  |  |  | Rb1cc1 |  |
|  |  |  |  |  |  |  | Rbck1 |  |
|  |  |  |  |  |  |  | Rbfox2 |  |
|  |  |  |  |  |  |  | Rbm26 |  |
|  |  |  |  |  |  |  | Rbm39 |  |
|  |  |  |  |  |  |  | Rbm42 |  |
|  |  |  |  |  |  |  | Rbms1 |  |
|  |  |  |  |  |  |  | Rbms2 |  |
|  |  |  |  |  |  |  | Rbpj |  |
|  |  |  |  |  |  |  | Rbx1 |  |
|  |  |  |  |  |  |  | Rc3h2 |  |
|  |  |  |  |  |  |  | Rcc2 |  |
|  |  |  |  |  |  |  | Rcn3 |  |
|  |  |  |  |  |  |  | Rcor3 |  |
|  |  |  |  |  |  |  | Rdm1 |  |
|  |  |  |  |  |  |  | Reep3 |  |
|  |  |  |  |  |  |  | Rela |  |
|  |  |  |  |  |  |  | Relb |  |
|  |  |  |  |  |  |  | Rest |  |
|  |  |  |  |  |  |  | Retsat |  |
|  |  |  |  |  |  |  | Rftn1 |  |
|  |  |  |  |  |  |  | Rfwd2 |  |
|  |  |  |  |  |  |  | Rgcc |  |
|  |  |  |  |  |  |  | RGD1303003 |  |
|  |  |  |  |  |  |  | RGD1303130 |  |
|  |  |  |  |  |  |  | RGD1308428 |  |
|  |  |  |  |  |  |  | RGD1309995 |  |
|  |  |  |  |  |  |  | RGD1310016 |  |
|  |  |  |  |  |  |  | RGD1310352 |  |
|  |  |  |  |  |  |  | RGD1311739 |  |
|  |  |  |  |  |  |  | RGD1311805 |  |
|  |  |  |  |  |  |  | RGD1559896 |  |
|  |  |  |  |  |  |  | RGD1559909 |  |
|  |  |  |  |  |  |  | RGD1561897 |  |
|  |  |  |  |  |  |  | RGD1562114 |  |
|  |  |  |  |  |  |  | RGD1565616 |  |
|  |  |  |  |  |  |  | RGD735065 |  |
|  |  |  |  |  |  |  | Rgp1 |  |
|  |  |  |  |  |  |  | Rhbdf1 |  |
|  |  |  |  |  |  |  | Rhob |  |
|  |  |  |  |  |  |  | Rhoj |  |
|  |  |  |  |  |  |  | Rhoq |  |
|  |  |  |  |  |  |  | Ric8a |  |
|  |  |  |  |  |  |  | Rims1 |  |
|  |  |  |  |  |  |  | Rin3 |  |
|  |  |  |  |  |  |  | Rlim |  |
|  |  |  |  |  |  |  | Rmnd5a |  |
|  |  |  |  |  |  |  | Rn50_14_0846.1 |  |
|  |  |  |  |  |  |  | Rn50_X_0746.3 |  |
|  |  |  |  |  |  |  | Rnase4 |  |
|  |  |  |  |  |  |  | Rnd1 |  |
|  |  |  |  |  |  |  | Rnd3 |  |
|  |  |  |  |  |  |  | Rnf111 |  |
|  |  |  |  |  |  |  | Rnf114 |  |
|  |  |  |  |  |  |  | Rnf115 |  |
|  |  |  |  |  |  |  | Rnf123 |  |
|  |  |  |  |  |  |  | Rnf14 |  |
|  |  |  |  |  |  |  | Rnf145 |  |
|  |  |  |  |  |  |  | Rnf181 |  |
|  |  |  |  |  |  |  | Rnf187 |  |
|  |  |  |  |  |  |  | Rnf19b |  |
|  |  |  |  |  |  |  | Rnf2 |  |
|  |  |  |  |  |  |  | Rnf38 |  |
|  |  |  |  |  |  |  | rno-mir-3064 |  |
|  |  |  |  |  |  |  | Rora |  |
|  |  |  |  |  |  |  | Rpl7l1 |  |
|  |  |  |  |  |  |  | Rpp14 |  |
|  |  |  |  |  |  |  | Rps6kb1 |  |
|  |  |  |  |  |  |  | Rps6kc1 |  |
|  |  |  |  |  |  |  | Rraga |  |
|  |  |  |  |  |  |  | Rras2 |  |
|  |  |  |  |  |  |  | Rreb1 |  |
|  |  |  |  |  |  |  | RT1-A1 |  |
|  |  |  |  |  |  |  | RT1-CE5 |  |
|  |  |  |  |  |  |  | RT1-CE7 |  |
|  |  |  |  |  |  |  | Rtfdc1 |  |
|  |  |  |  |  |  |  | Rtn3 |  |
|  |  |  |  |  |  |  | **Runx1** |  |
|  |  |  |  |  |  |  | Runx2 |  |
|  |  |  |  |  |  |  | Rxra |  |
|  |  |  |  |  |  |  | Rybp |  |
|  |  |  |  |  |  |  | Ryk |  |
|  |  |  |  |  |  |  | Sacm1l |  |
|  |  |  |  |  |  |  | Safb |  |
|  |  |  |  |  |  |  | Samd4b |  |
|  |  |  |  |  |  |  | Sap30l |  |
|  |  |  |  |  |  |  | Sar1a |  |
|  |  |  |  |  |  |  | Saraf |  |
|  |  |  |  |  |  |  | Sbf1 |  |
|  |  |  |  |  |  |  | Sbno1 |  |
|  |  |  |  |  |  |  | Sbno2 |  |
|  |  |  |  |  |  |  | Scaf1 |  |
|  |  |  |  |  |  |  | Scaf4 |  |
|  |  |  |  |  |  |  | Scaf8 |  |
|  |  |  |  |  |  |  | Scamp3 |  |
|  |  |  |  |  |  |  | Scamp5 |  |
|  |  |  |  |  |  |  | Scarb2 |  |
|  |  |  |  |  |  |  | Scn1b |  |
|  |  |  |  |  |  |  | Scoc |  |
|  |  |  |  |  |  |  | Scp2 |  |
|  |  |  |  |  |  |  | Scpep1 |  |
|  |  |  |  |  |  |  | Scrib |  |
|  |  |  |  |  |  |  | Sdc1 |  |
|  |  |  |  |  |  |  | Sdc3 |  |
|  |  |  |  |  |  |  | Sdcbp |  |
|  |  |  |  |  |  |  | Sdhc |  |
|  |  |  |  |  |  |  | Sec14l1 |  |
|  |  |  |  |  |  |  | Sec22b |  |
|  |  |  |  |  |  |  | Sec24a |  |
|  |  |  |  |  |  |  | Sec24b |  |
|  |  |  |  |  |  |  | Secisbp2l |  |
|  |  |  |  |  |  |  | Selt |  |
|  |  |  |  |  |  |  | Sema3c |  |
|  |  |  |  |  |  |  | Sepn1 |  |
|  |  |  |  |  |  |  | Sept11 |  |
|  |  |  |  |  |  |  | Sept8 |  |
|  |  |  |  |  |  |  | Serinc1 |  |
|  |  |  |  |  |  |  | Serinc3 |  |
|  |  |  |  |  |  |  | Serpina3n |  |
|  |  |  |  |  |  |  | Serpinb6 |  |
|  |  |  |  |  |  |  | Serpinb9 |  |
|  |  |  |  |  |  |  | Serpine1 |  |
|  |  |  |  |  |  |  | Sertad2 |  |
|  |  |  |  |  |  |  | Sertad4 |  |
|  |  |  |  |  |  |  | Sestd1 |  |
|  |  |  |  |  |  |  | SETD1B |  |
|  |  |  |  |  |  |  | Setd7 |  |
|  |  |  |  |  |  |  | Sf3b1 |  |
|  |  |  |  |  |  |  | Sf3b3 |  |
|  |  |  |  |  |  |  | Sf3b4 |  |
|  |  |  |  |  |  |  | Sfpq |  |
|  |  |  |  |  |  |  | Sfrp1 |  |
|  |  |  |  |  |  |  | Sft2d1 |  |
|  |  |  |  |  |  |  | Sfxn3 |  |
|  |  |  |  |  |  |  | Sgms1 |  |
|  |  |  |  |  |  |  | Sh3gl1 |  |
|  |  |  |  |  |  |  | Sh3pxd2a |  |
|  |  |  |  |  |  |  | Sh3pxd2b |  |
|  |  |  |  |  |  |  | Sharpin |  |
|  |  |  |  |  |  |  | Shb |  |
|  |  |  |  |  |  |  | Shc1 |  |
|  |  |  |  |  |  |  | Shisa5 |  |
|  |  |  |  |  |  |  | Shoc2 |  |
|  |  |  |  |  |  |  | Shroom4 |  |
|  |  |  |  |  |  |  | Sidt2 |  |
|  |  |  |  |  |  |  | Sik3 |  |
|  |  |  |  |  |  |  | Ski |  |
|  |  |  |  |  |  |  | Slbp |  |
|  |  |  |  |  |  |  | Slc12a4 |  |
|  |  |  |  |  |  |  | Slc1a4 |  |
|  |  |  |  |  |  |  | Slc22a17 |  |
|  |  |  |  |  |  |  | Slc25a36l1 |  |
|  |  |  |  |  |  |  | Slc25a37 |  |
|  |  |  |  |  |  |  | Slc29a1 |  |
|  |  |  |  |  |  |  | Slc2a3 |  |
|  |  |  |  |  |  |  | Slc30a5 |  |
|  |  |  |  |  |  |  | Slc30a9 |  |
|  |  |  |  |  |  |  | Slc35a4 |  |
|  |  |  |  |  |  |  | Slc35b1 |  |
|  |  |  |  |  |  |  | Slc35b4 |  |
|  |  |  |  |  |  |  | Slc35f5 |  |
|  |  |  |  |  |  |  | Slc37a3 |  |
|  |  |  |  |  |  |  | Slc38a2 |  |
|  |  |  |  |  |  |  | Slc39a13 |  |
|  |  |  |  |  |  |  | Slc39a6 |  |
|  |  |  |  |  |  |  | Slc39a7 |  |
|  |  |  |  |  |  |  | Slc44a1 |  |
|  |  |  |  |  |  |  | Slc44a2 |  |
|  |  |  |  |  |  |  | Slc6a6 |  |
|  |  |  |  |  |  |  | Slc6a8 |  |
|  |  |  |  |  |  |  | Slc7a2 |  |
|  |  |  |  |  |  |  | Slc7a5 |  |
|  |  |  |  |  |  |  | Slit3 |  |
|  |  |  |  |  |  |  | Slpil2 |  |
|  |  |  |  |  |  |  | Smad1 |  |
|  |  |  |  |  |  |  | Smad2 |  |
|  |  |  |  |  |  |  | Smad4 |  |
|  |  |  |  |  |  |  | Smad6 |  |
|  |  |  |  |  |  |  | Smad7 |  |
|  |  |  |  |  |  |  | Smarcd2 |  |
|  |  |  |  |  |  |  | Smg5 |  |
|  |  |  |  |  |  |  | Smim1 |  |
|  |  |  |  |  |  |  | Smim14 |  |
|  |  |  |  |  |  |  | Smim19 |  |
|  |  |  |  |  |  |  | Smpd1 |  |
|  |  |  |  |  |  |  | Sms |  |
|  |  |  |  |  |  |  | Smurf1 |  |
|  |  |  |  |  |  |  | Smyd2 |  |
|  |  |  |  |  |  |  | Snai1 |  |
|  |  |  |  |  |  |  | Snapc3 |  |
|  |  |  |  |  |  |  | Snd1 |  |
|  |  |  |  |  |  |  | Snrnp48 |  |
|  |  |  |  |  |  |  | Snrpa |  |
|  |  |  |  |  |  |  | Snrpn |  |
|  |  |  |  |  |  |  | Snx12 |  |
|  |  |  |  |  |  |  | Snx14 |  |
|  |  |  |  |  |  |  | Snx17 |  |
|  |  |  |  |  |  |  | Snx18 |  |
|  |  |  |  |  |  |  | Snx24 |  |
|  |  |  |  |  |  |  | Snx29 |  |
|  |  |  |  |  |  |  | Snx3 |  |
|  |  |  |  |  |  |  | Snx4 |  |
|  |  |  |  |  |  |  | Snx5 |  |
|  |  |  |  |  |  |  | Socs3 |  |
|  |  |  |  |  |  |  | Socs5 |  |
|  |  |  |  |  |  |  | Sord |  |
|  |  |  |  |  |  |  | Sort1 |  |
|  |  |  |  |  |  |  | Sox9 |  |
|  |  |  |  |  |  |  | Sp3 |  |
|  |  |  |  |  |  |  | Spata5 |  |
|  |  |  |  |  |  |  | Spcs2 |  |
|  |  |  |  |  |  |  | Spcs3 |  |
|  |  |  |  |  |  |  | Spef1 |  |
|  |  |  |  |  |  |  | Spg21 |  |
|  |  |  |  |  |  |  | Spg7 |  |
|  |  |  |  |  |  |  | Spin1 |  |
|  |  |  |  |  |  |  | Spint2 |  |
|  |  |  |  |  |  |  | Spopl |  |
|  |  |  |  |  |  |  | Sppl3 |  |
|  |  |  |  |  |  |  | Spred1 |  |
|  |  |  |  |  |  |  | Spry2 |  |
|  |  |  |  |  |  |  | Src |  |
|  |  |  |  |  |  |  | Srd5a3 |  |
|  |  |  |  |  |  |  | Srgap2 |  |
|  |  |  |  |  |  |  | Srm |  |
|  |  |  |  |  |  |  | Srpr |  |
|  |  |  |  |  |  |  | Srpx2 |  |
|  |  |  |  |  |  |  | Srr |  |
|  |  |  |  |  |  |  | Srrm2 |  |
|  |  |  |  |  |  |  | Srsf1 |  |
|  |  |  |  |  |  |  | Srsf2 |  |
|  |  |  |  |  |  |  | Ss18 |  |
|  |  |  |  |  |  |  | Ssbp2 |  |
|  |  |  |  |  |  |  | Ssbp3 |  |
|  |  |  |  |  |  |  | Ssbp4 |  |
|  |  |  |  |  |  |  | Ssh3 |  |
|  |  |  |  |  |  |  | Ssr3 |  |
|  |  |  |  |  |  |  | Sssca1 |  |
|  |  |  |  |  |  |  | St3gal1 |  |
|  |  |  |  |  |  |  | St3gal2 |  |
|  |  |  |  |  |  |  | St5 |  |
|  |  |  |  |  |  |  | Stam |  |
|  |  |  |  |  |  |  | Stard7 |  |
|  |  |  |  |  |  |  | Stat2 |  |
|  |  |  |  |  |  |  | Stat3 |  |
|  |  |  |  |  |  |  | Stat5b |  |
|  |  |  |  |  |  |  | Stat6 |  |
|  |  |  |  |  |  |  | Stau1 |  |
|  |  |  |  |  |  |  | Stc2 |  |
|  |  |  |  |  |  |  | Stk11 |  |
|  |  |  |  |  |  |  | Stom |  |
|  |  |  |  |  |  |  | Strn |  |
|  |  |  |  |  |  |  | Strn3 |  |
|  |  |  |  |  |  |  | Stt3a |  |
|  |  |  |  |  |  |  | Stt3b |  |
|  |  |  |  |  |  |  | Stub1 |  |
|  |  |  |  |  |  |  | Stx3 |  |
|  |  |  |  |  |  |  | Stx5 |  |
|  |  |  |  |  |  |  | Stx6 |  |
|  |  |  |  |  |  |  | Stxbp5 |  |
|  |  |  |  |  |  |  | Styx |  |
|  |  |  |  |  |  |  | Sulf2 |  |
|  |  |  |  |  |  |  | Sumo3 |  |
|  |  |  |  |  |  |  | Supt20 |  |
|  |  |  |  |  |  |  | Surf1 |  |
|  |  |  |  |  |  |  | Surf4 |  |
|  |  |  |  |  |  |  | Svep1 |  |
|  |  |  |  |  |  |  | Syde1 |  |
|  |  |  |  |  |  |  | Sympk |  |
|  |  |  |  |  |  |  | Syngr2 |  |
|  |  |  |  |  |  |  | Tab2 |  |
|  |  |  |  |  |  |  | Taf10 |  |
|  |  |  |  |  |  |  | Taf15 |  |
|  |  |  |  |  |  |  | Tagln2 |  |
|  |  |  |  |  |  |  | Taok1 |  |
|  |  |  |  |  |  |  | Tap1 |  |
|  |  |  |  |  |  |  | Tapbp |  |
|  |  |  |  |  |  |  | Tardbp |  |
|  |  |  |  |  |  |  | Tatdn2 |  |
|  |  |  |  |  |  |  | Tax1bp3 |  |
|  |  |  |  |  |  |  | Tbc1d10a |  |
|  |  |  |  |  |  |  | Tbc1d13 |  |
|  |  |  |  |  |  |  | Tbc1d20 |  |
|  |  |  |  |  |  |  | Tbc1d2b |  |
|  |  |  |  |  |  |  | Tbcd |  |
|  |  |  |  |  |  |  | Tbl1x |  |
|  |  |  |  |  |  |  | Tbl1xr1 |  |
|  |  |  |  |  |  |  | Tbrg4 |  |
|  |  |  |  |  |  |  | Tbx20 |  |
|  |  |  |  |  |  |  | Tcf12 |  |
|  |  |  |  |  |  |  | Tcf3 |  |
|  |  |  |  |  |  |  | Tdg |  |
|  |  |  |  |  |  |  | Tead3 |  |
|  |  |  |  |  |  |  | Tenc1 |  |
|  |  |  |  |  |  |  | Tet3 |  |
|  |  |  |  |  |  |  | Tex264 |  |
|  |  |  |  |  |  |  | Tf |  |
|  |  |  |  |  |  |  | Tfe3 |  |
|  |  |  |  |  |  |  | Tgfb1 |  |
|  |  |  |  |  |  |  | Tgfb2 |  |
|  |  |  |  |  |  |  | Tgfbi |  |
|  |  |  |  |  |  |  | Tgif1 |  |
|  |  |  |  |  |  |  | Tgm2 |  |
|  |  |  |  |  |  |  | Tgoln2 |  |
|  |  |  |  |  |  |  | Thra |  |
|  |  |  |  |  |  |  | Thumpd1 |  |
|  |  |  |  |  |  |  | Thyn1 |  |
|  |  |  |  |  |  |  | Tia1 |  |
|  |  |  |  |  |  |  | Tjap1 |  |
|  |  |  |  |  |  |  | Tlk1 |  |
|  |  |  |  |  |  |  | Tln1 |  |
|  |  |  |  |  |  |  | Tm2d2 |  |
|  |  |  |  |  |  |  | Tm9sf2 |  |
|  |  |  |  |  |  |  | Tmbim4 |  |
|  |  |  |  |  |  |  | Tmbim6 |  |
|  |  |  |  |  |  |  | Tmed1 |  |
|  |  |  |  |  |  |  | Tmed3 |  |
|  |  |  |  |  |  |  | Tmed7 |  |
|  |  |  |  |  |  |  | Tmem106b |  |
|  |  |  |  |  |  |  | Tmem110 |  |
|  |  |  |  |  |  |  | Tmem120a |  |
|  |  |  |  |  |  |  | Tmem123 |  |
|  |  |  |  |  |  |  | Tmem132a |  |
|  |  |  |  |  |  |  | Tmem150a |  |
|  |  |  |  |  |  |  | Tmem167a |  |
|  |  |  |  |  |  |  | Tmem167b |  |
|  |  |  |  |  |  |  | Tmem175 |  |
|  |  |  |  |  |  |  | Tmem176a |  |
|  |  |  |  |  |  |  | Tmem179b |  |
|  |  |  |  |  |  |  | Tmem181 |  |
|  |  |  |  |  |  |  | Tmem183a |  |
|  |  |  |  |  |  |  | Tmem184b |  |
|  |  |  |  |  |  |  | Tmem214 |  |
|  |  |  |  |  |  |  | Tmem245 |  |
|  |  |  |  |  |  |  | Tmem248 |  |
|  |  |  |  |  |  |  | Tmem259 |  |
|  |  |  |  |  |  |  | Tmem263 |  |
|  |  |  |  |  |  |  | Tmem33 |  |
|  |  |  |  |  |  |  | Tmem43 |  |
|  |  |  |  |  |  |  | Tmem47 |  |
|  |  |  |  |  |  |  | Tmem55b |  |
|  |  |  |  |  |  |  | Tmem63b |  |
|  |  |  |  |  |  |  | Tmem98 |  |
|  |  |  |  |  |  |  | Tmpo |  |
|  |  |  |  |  |  |  | Tmtc1 |  |
|  |  |  |  |  |  |  | Tmx3 |  |
|  |  |  |  |  |  |  | Tmx4 |  |
|  |  |  |  |  |  |  | Tnfaip1 |  |
|  |  |  |  |  |  |  | Tnfaip2 |  |
|  |  |  |  |  |  |  | Tnfrsf11b |  |
|  |  |  |  |  |  |  | Tnfrsf12a |  |
|  |  |  |  |  |  |  | Tnfrsf22 |  |
|  |  |  |  |  |  |  | Tnk2 |  |
|  |  |  |  |  |  |  | Tnks |  |
|  |  |  |  |  |  |  | Tom1 |  |
|  |  |  |  |  |  |  | Tom1l2 |  |
|  |  |  |  |  |  |  | Tomm20 |  |
|  |  |  |  |  |  |  | Tomm6 |  |
|  |  |  |  |  |  |  | Tomm70a |  |
|  |  |  |  |  |  |  | Tor1aip1 |  |
|  |  |  |  |  |  |  | Tor1aip2 |  |
|  |  |  |  |  |  |  | Tox4 |  |
|  |  |  |  |  |  |  | Tp53 |  |
|  |  |  |  |  |  |  | Tp53inp1 |  |
|  |  |  |  |  |  |  | Tp53inp2 |  |
|  |  |  |  |  |  |  | Tpd52l2 |  |
|  |  |  |  |  |  |  | Tpi1 |  |
|  |  |  |  |  |  |  | Tpm4 |  |
|  |  |  |  |  |  |  | Tpp1 |  |
|  |  |  |  |  |  |  | Tpra1 |  |
|  |  |  |  |  |  |  | Tprg1l |  |
|  |  |  |  |  |  |  | Tpst2 |  |
|  |  |  |  |  |  |  | Trabd2b |  |
|  |  |  |  |  |  |  | Traf2 |  |
|  |  |  |  |  |  |  | Traf4 |  |
|  |  |  |  |  |  |  | Trafd1 |  |
|  |  |  |  |  |  |  | Trak1 |  |
|  |  |  |  |  |  |  | Trak2 |  |
|  |  |  |  |  |  |  | Tram1 |  |
|  |  |  |  |  |  |  | Trim25 |  |
|  |  |  |  |  |  |  | Trim26 |  |
|  |  |  |  |  |  |  | Trim28 |  |
|  |  |  |  |  |  |  | Trim44 |  |
|  |  |  |  |  |  |  | Trim47 |  |
|  |  |  |  |  |  |  | Trim8 |  |
|  |  |  |  |  |  |  | Trip10 |  |
|  |  |  |  |  |  |  | Trpc4ap |  |
|  |  |  |  |  |  |  | Trpm7 |  |
|  |  |  |  |  |  |  | Trub2 |  |
|  |  |  |  |  |  |  | Tsc22d1 |  |
|  |  |  |  |  |  |  | Tsc22d2 |  |
|  |  |  |  |  |  |  | Tsen34 |  |
|  |  |  |  |  |  |  | Tsn |  |
|  |  |  |  |  |  |  | Tsnax |  |
|  |  |  |  |  |  |  | Tspan14 |  |
|  |  |  |  |  |  |  | Tspan3 |  |
|  |  |  |  |  |  |  | Tspan31 |  |
|  |  |  |  |  |  |  | Tspan4 |  |
|  |  |  |  |  |  |  | Tspan6 |  |
|  |  |  |  |  |  |  | Tspo |  |
|  |  |  |  |  |  |  | Tsr1 |  |
|  |  |  |  |  |  |  | Ttc37 |  |
|  |  |  |  |  |  |  | Ttc39b |  |
|  |  |  |  |  |  |  | Ttyh3 |  |
|  |  |  |  |  |  |  | Tuba4a |  |
|  |  |  |  |  |  |  | Tulp4 |  |
|  |  |  |  |  |  |  | Tusc2 |  |
|  |  |  |  |  |  |  | Tusc3 |  |
|  |  |  |  |  |  |  | **Twist1** |  |
|  |  |  |  |  |  |  | Txndc5 |  |
|  |  |  |  |  |  |  | Uap1l1 |  |
|  |  |  |  |  |  |  | Uba1 |  |
|  |  |  |  |  |  |  | Ubald1 |  |
|  |  |  |  |  |  |  | Ubald2 |  |
|  |  |  |  |  |  |  | Ubap2 |  |
|  |  |  |  |  |  |  | Ube2b |  |
|  |  |  |  |  |  |  | Ube2d2 |  |
|  |  |  |  |  |  |  | Ube2e1 |  |
|  |  |  |  |  |  |  | Ube2f |  |
|  |  |  |  |  |  |  | Ube2g1 |  |
|  |  |  |  |  |  |  | Ube2g2 |  |
|  |  |  |  |  |  |  | Ube2j1 |  |
|  |  |  |  |  |  |  | Ube2k |  |
|  |  |  |  |  |  |  | Ube2l3 |  |
|  |  |  |  |  |  |  | Ube2o |  |
|  |  |  |  |  |  |  | Ube2v1 |  |
|  |  |  |  |  |  |  | Ubfd1 |  |
|  |  |  |  |  |  |  | Ubl3 |  |
|  |  |  |  |  |  |  | Ubl5 |  |
|  |  |  |  |  |  |  | Ubn1 |  |
|  |  |  |  |  |  |  | Ubqln2 |  |
|  |  |  |  |  |  |  | Ubtd1 |  |
|  |  |  |  |  |  |  | Uchl1 |  |
|  |  |  |  |  |  |  | Uck2 |  |
|  |  |  |  |  |  |  | Ucp2 |  |
|  |  |  |  |  |  |  | Ugcg |  |
|  |  |  |  |  |  |  | Ugdh |  |
|  |  |  |  |  |  |  | Upf1 |  |
|  |  |  |  |  |  |  | Usf2 |  |
|  |  |  |  |  |  |  | Usmg5 |  |
|  |  |  |  |  |  |  | Usp12 |  |
|  |  |  |  |  |  |  | Usp14 |  |
|  |  |  |  |  |  |  | Usp18 |  |
|  |  |  |  |  |  |  | Usp22 |  |
|  |  |  |  |  |  |  | Usp25 |  |
|  |  |  |  |  |  |  | Usp3 |  |
|  |  |  |  |  |  |  | Usp31 |  |
|  |  |  |  |  |  |  | Usp34 |  |
|  |  |  |  |  |  |  | Usp7 |  |
|  |  |  |  |  |  |  | Usp9x |  |
|  |  |  |  |  |  |  | Uvrag |  |
|  |  |  |  |  |  |  | Uxt |  |
|  |  |  |  |  |  |  | Vamp2 |  |
|  |  |  |  |  |  |  | Vamp3 |  |
|  |  |  |  |  |  |  | Vamp5 |  |
|  |  |  |  |  |  |  | Vamp7 |  |
|  |  |  |  |  |  |  | Vash1 |  |
|  |  |  |  |  |  |  | Vat1 |  |
|  |  |  |  |  |  |  | Vcam1 |  |
|  |  |  |  |  |  |  | Vcl |  |
|  |  |  |  |  |  |  | Vdac1 |  |
|  |  |  |  |  |  |  | Vegfa |  |
|  |  |  |  |  |  |  | Vegfb |  |
|  |  |  |  |  |  |  | Vegfc |  |
|  |  |  |  |  |  |  | Vezf1 |  |
|  |  |  |  |  |  |  | Vgll4 |  |
|  |  |  |  |  |  |  | Vim |  |
|  |  |  |  |  |  |  | Vmp1 |  |
|  |  |  |  |  |  |  | Vom2r52 |  |
|  |  |  |  |  |  |  | Vps11 |  |
|  |  |  |  |  |  |  | Vps26b |  |
|  |  |  |  |  |  |  | Vps52 |  |
|  |  |  |  |  |  |  | Vta1 |  |
|  |  |  |  |  |  |  | Wapal |  |
|  |  |  |  |  |  |  | Wars |  |
|  |  |  |  |  |  |  | Wasl |  |
|  |  |  |  |  |  |  | Wbp1l |  |
|  |  |  |  |  |  |  | Wbp2 |  |
|  |  |  |  |  |  |  | Wdfy1 |  |
|  |  |  |  |  |  |  | Wdfy2 |  |
|  |  |  |  |  |  |  | Wdr26 |  |
|  |  |  |  |  |  |  | Wdr43 |  |
|  |  |  |  |  |  |  | Wdr77 |  |
|  |  |  |  |  |  |  | Wee1 |  |
|  |  |  |  |  |  |  | Wfdc1 |  |
|  |  |  |  |  |  |  | Wipi2 |  |
|  |  |  |  |  |  |  | Wnk1 |  |
|  |  |  |  |  |  |  | Wtip |  |
|  |  |  |  |  |  |  | Wwc2 |  |
|  |  |  |  |  |  |  | Wwtr1 |  |
|  |  |  |  |  |  |  | Xbp1 |  |
|  |  |  |  |  |  |  | Xpo1 |  |
|  |  |  |  |  |  |  | Xpo6 |  |
|  |  |  |  |  |  |  | Xpo7 |  |
|  |  |  |  |  |  |  | Xpr1 |  |
|  |  |  |  |  |  |  | Yif1b |  |
|  |  |  |  |  |  |  | Yipf2 |  |
|  |  |  |  |  |  |  | Yipf3 |  |
|  |  |  |  |  |  |  | Yipf4 |  |
|  |  |  |  |  |  |  | Ylpm1 |  |
|  |  |  |  |  |  |  | Ypel3 |  |
|  |  |  |  |  |  |  | Ypel5 |  |
|  |  |  |  |  |  |  | Ythdf1 |  |
|  |  |  |  |  |  |  | Ythdf2 |  |
|  |  |  |  |  |  |  | Ythdf3 |  |
|  |  |  |  |  |  |  | Ywhag |  |
|  |  |  |  |  |  |  | Ywhah |  |
|  |  |  |  |  |  |  | Ywhaz |  |
|  |  |  |  |  |  |  | Yy1 |  |
|  |  |  |  |  |  |  | Zak |  |
|  |  |  |  |  |  |  | Zc2hc1a |  |
|  |  |  |  |  |  |  | Zc3hav1 |  |
|  |  |  |  |  |  |  | Zcchc24 |  |
|  |  |  |  |  |  |  | Zdhhc16 |  |
|  |  |  |  |  |  |  | Zdhhc5 |  |
|  |  |  |  |  |  |  | Zfand5 |  |
|  |  |  |  |  |  |  | Zfand6 |  |
|  |  |  |  |  |  |  | Zfp106 |  |
|  |  |  |  |  |  |  | Zfp219 |  |
|  |  |  |  |  |  |  | Zfp36 |  |
|  |  |  |  |  |  |  | Zfp36l1 |  |
|  |  |  |  |  |  |  | Zfp385a |  |
|  |  |  |  |  |  |  | Zfp407 |  |
|  |  |  |  |  |  |  | Zfp521 |  |
|  |  |  |  |  |  |  | Zfp664 |  |
|  |  |  |  |  |  |  | Zfp703 |  |
|  |  |  |  |  |  |  | Zfp706 |  |
|  |  |  |  |  |  |  | Zfpm1 |  |
|  |  |  |  |  |  |  | Zfr |  |
|  |  |  |  |  |  |  | Zmiz1 |  |
|  |  |  |  |  |  |  | Zmym2 |  |
|  |  |  |  |  |  |  | Zranb2 |  |
|  |  |  |  |  |  |  | Zyx |  |
|  |  |  |  |  |  |  | Zzz3 |  |
|  |  |  |  |  |  |  | **Rock2** |  |
|  |  |  |  |  |  |  | AABR06004278.1 |  |
|  |  |  |  |  |  |  | AABR06004393.1 |  |
|  |  |  |  |  |  |  | AABR06004819.1 |  |
|  |  |  |  |  |  |  | AABR06007110.1 |  |
|  |  |  |  |  |  |  | AABR06008886.1 |  |
|  |  |  |  |  |  |  | AABR06010677.1 |  |
|  |  |  |  |  |  |  | AABR06011545.1 |  |
|  |  |  |  |  |  |  | AABR06018690.1 |  |
|  |  |  |  |  |  |  | AABR06022945.1 |  |
|  |  |  |  |  |  |  | AABR06025204.1 |  |
|  |  |  |  |  |  |  | AABR06028169.1 |  |
|  |  |  |  |  |  |  | AABR06032175.1 |  |
|  |  |  |  |  |  |  | AABR06034751.1 |  |
|  |  |  |  |  |  |  | AABR06040671.1 |  |
|  |  |  |  |  |  |  | AABR06041411.1 |  |
|  |  |  |  |  |  |  | AABR06042581.1 |  |
|  |  |  |  |  |  |  | AABR06046187.1 |  |
|  |  |  |  |  |  |  | AABR06047970.1 |  |
|  |  |  |  |  |  |  | AABR06053360.1 |  |
|  |  |  |  |  |  |  | AABR06054457.1 |  |
|  |  |  |  |  |  |  | AABR06054504.1 |  |
|  |  |  |  |  |  |  | AABR06055536.1 |  |
|  |  |  |  |  |  |  | AABR06055554.1 |  |
|  |  |  |  |  |  |  | AABR06055555.1 |  |
|  |  |  |  |  |  |  | AABR06056275.1 |  |
|  |  |  |  |  |  |  | AABR06061012.1 |  |
|  |  |  |  |  |  |  | AABR06065806.1 |  |
|  |  |  |  |  |  |  | AABR06066181.1 |  |
|  |  |  |  |  |  |  | AABR06066437.1 |  |
|  |  |  |  |  |  |  | AABR06068952.1 |  |
|  |  |  |  |  |  |  | AABR06069329.2 |  |
|  |  |  |  |  |  |  | AABR06070446.1 |  |
|  |  |  |  |  |  |  | AABR06076212.1 |  |
|  |  |  |  |  |  |  | AABR06077351.1 |  |
|  |  |  |  |  |  |  | AABR06078351.1 |  |
|  |  |  |  |  |  |  | AABR06078903.1 |  |
|  |  |  |  |  |  |  | AABR06083238.2 |  |
|  |  |  |  |  |  |  | AABR06083238.3 |  |
|  |  |  |  |  |  |  | AABR06086857.1 |  |
|  |  |  |  |  |  |  | AABR06088579.1 |  |
|  |  |  |  |  |  |  | AABR06089031.1 |  |
|  |  |  |  |  |  |  | AABR06089464.1 |  |
|  |  |  |  |  |  |  | AABR06091411.1 |  |
|  |  |  |  |  |  |  | AABR06091621.1 |  |
|  |  |  |  |  |  |  | AABR06099253.1 |  |
|  |  |  |  |  |  |  | AABR06103011.2 |  |
|  |  |  |  |  |  |  | AABR06109214.1 |  |
